# Regioselective Fluorohydrin Synthesis from Allylsilanes and Evidence for a Silicon–Fluorine Gauche Effect

**DOI:** 10.1021/acs.joc.3c02163

**Published:** 2024-03-08

**Authors:** Alexie W. Clover, Adam P. Jones, Robert F. Berger, Werner Kaminsky, Gregory. W. O’Neil

**Affiliations:** † Department of Chemistry, Western Washington University, Bellingham, Washington 98229, United States; ‡ Department of Chemistry, University of Washington, Seattle, Washington 98195, United States

## Abstract

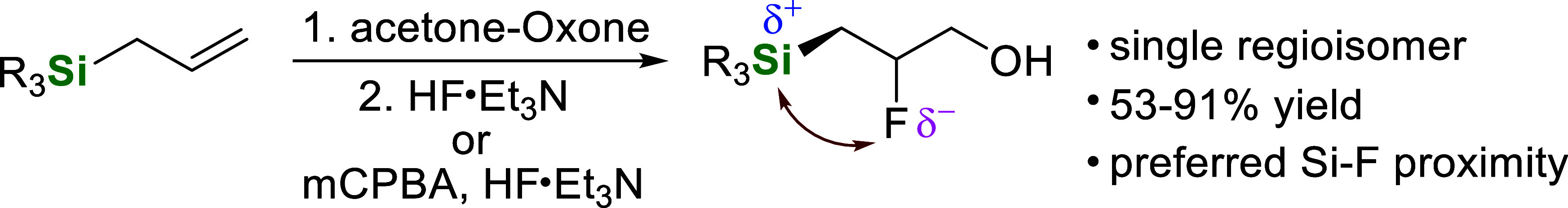

Allylsilanes can be regioselectively transformed into the corresponding 3-silylfluorohydrin in good yield using a sequence of epoxidation followed by treatment with HF·Et_3_N with or without isolation of the intermediate epoxide. Various silicon-substitutions are tolerated, resulting in a range of 2-fluoro-3-silylpropan-1-ol products from this method. Whereas other fluorohydrin syntheses by epoxide opening using HF·Et_3_N generally require more forcing conditions (e.g., higher reaction temperature), opening of allylsilane-derived epoxides with this reagent occurs at room temperature. We attribute this rate acceleration along with the observed regioselectivity to a β-silyl effect that stabilizes a proposed cationic intermediate. The use of enantioenriched epoxides indicates that both S_N_1- and S_N_2-type mechanisms may be operable depending on substitution at silicon. Conformational analysis by NMR and theory along with a crystal structure obtained by X-ray diffraction points to a preference for silicon and fluorine to be proximal to one another in the products, perhaps favored due to electrostatic interactions.

## Introduction

The introduction of fluorine atoms is an established tool for modulating the physicochemical properties of organic molecules, used widely in the pharmaceutical industry to improve selectivity, potency, and pharmacokinetic properties of active ingredients.^1−7^ New methods continue to emerge for the preparation of organofluorine compounds, including both catalytic and enantioselective systems.^8^ The ongoing need of drug discovery programs for fluorine-containing building blocks makes important the development of reliable, scalable, and selective strategies to generate organofluorine chemicals capable of further functionalization.^9,10^ Herein, we report the synthesis of 2-fluoro-3-silylpropan-1-ols from allylsilanes by a sequence of epoxidation and epoxide opening with HF·Et_3_N. Epoxide opening occurs with complete regioselectivity and appears to proceed via a blend of S_N_1 and S_N_2 mechanisms depending on substitution at silicon. Analysis of the 2-fluoro-3-silylpropan-1-ol products revealed a conformational preference for silicon and fluorine to be in close proximity. Whereas preferred conformations of other fluorine-containing molecules is generally considered to be the result of hyperconjugation,^11−13^ we hypothesize that electrostatic interactions contribute to the observed conformational bias of 2-fluoro-3-silylpropan-1-ol systems.

## Results and Discussion

As part of an ongoing program investigating new reactions of allylsilanes,^14−16^ we observed that epoxysilanes, prepared by epoxidation of the corresponding allylsilane, are cleanly converted to the corresponding fluorohydrin upon treatment with triethylamine trihydrofluoride (HF·Et_3_N; Table 1).^17^ Other HF sources such as Olah’s reagent (HF·Py) resulted in significant decomposition. However, the addition of commercially available HF·Et_3_N (ca. 37% HF) to a solution of the epoxysilane in dichloromethane (DCM) at room temperature produced the 2-fluoro-3-silylpropan-1-ols in uniformly high yield and with complete regioselectivity. In a typical experiment, the allylsilane was epoxidized using in situ-prepared dimethyldioxirane.^18^ This generally gave a sufficiently pure epoxide that could be taken directly into the epoxide opening with HF·Et_3_N (“conditions B”, Table 1). An exception was allyltrimethylsilane, where the resulting epoxide proved somewhat volatile, making its isolation challenging. Instead, a one-pot epoxidation/epoxide opening adapted from the procedure of Sedgwick et al. was performed by the treatment of allyltrimethylsilane with mCPBA and HF·Et_3_N in DCM (“conditions A”, Table 1).^19^ Under these conditions, the corresponding fluorohydrin **1** was isolated in 65% yield (entry 1) containing small amounts (∼10%) of 1-hydroxy-3-(trimethylsilyl)propan-2-yl 3-chlorobenzoate, resulting from opening of the epoxide by mCPBA-derived 3-chlorobenzoic acid.^20,21^ The reaction was notably slower with phenyl-substituted epoxysilanes (entries 2, 4–7), where greater phenyl substitution resulted in longer required reaction times to achieve good yields (e.g., 72 h. for Ph_3_ (**2**, entry 2), 10 h. for (allyl)Ph_2_ (**5**, entry 6), and 4 h. for Me_2_Ph (**4**, entry 4); see the Experimental Section), which may reflect a change in the mechanism (vide infra). The highest yield was obtained from allyltriisopropylsilane (entry 3), which gave the corresponding fluorohydrin **3** in 92% yield using the two-step procedure. Yields for the two-step and one-pot procedures were generally comparable (e.g., entries 4 and 5). Low isolated yields of fluorohydrin products **5** and **6** from diallylsilanes (entries 6–8) were the result of reactions (e.g., epoxidation and/or opening/elimination) occurring at the other allyl group. The two-step procedure (conditions “A”) proved optimal for producing fluorohydrin **7** from allyl(bromomethyl)dimethylsilane (60% yield, entry 9), where multiple byproducts were formed from the one-pot process.

**Table 1:**
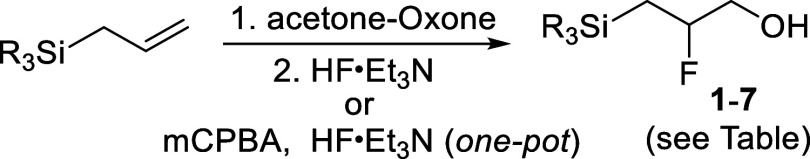
Fluorohydrin Synthesis from Allylsilanes

entry	cond.^a^	time (h)^c^	R_3_Si	yield (%)^b^
1	B	1	Me_3_Si (**1**)	65
2	A	24	Ph_3_Si (**2**)	65
3	A	1.5	*i*-Pr_3_Si (**3**)	92
4	A	4	PhMe_2_Si (**4**)	72
5	B	4	PhMe_2_Si (**4**)	60
6	A	16	(allyl)Ph_2_Si (**5**)	41
7	B	16	(allyl)Ph_2_Si (**5**)	53
8	B	1	(allyl)Me_2_Si (**6**)	56
9	A	2	(CH_2_Br)Me_2_Si (**7**)	60
10	B	2	(CH_2_Br)Me_2_Si (**7**)	37

a Notes for table: cond. A: 1. Allylsilane (1.0 mmol), tetrabutyl ammonium hydrogen sulfate (TBAHS) (4 mol %), acetone (30 mmol), K_2_CO_3_ (0.1 M) (0.2 mmol), dimethoxymethane (DMM)/MeCN (2:1) (8 mL), oxone (3 mmol), and K_2_CO_3_ (13.3 mmol), room temperature; 2. Epoxysilane (1 equiv), HF·Et_3_N (5 equiv),^22^ DCM (0.05–0.1 M), room temperature. Cond. B: allylsilane (1 equiv), mCPBA (1.3 equiv), HF·Et_3_N (5–7 equiv), DCM (0.05–0.1 M).

b Isolated yields of fluorohydrin from the starting allylsilane.

c Refers only to time of the HF·Et_3_N step for conditions A.

**Scheme 1:**
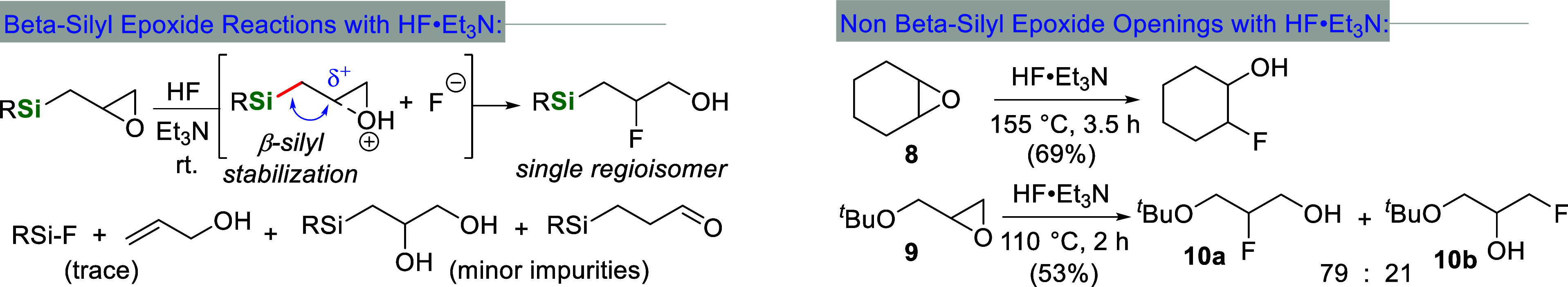
Comparison of HF·Et_3_N Openings of Epoxides with (Left) and without (Right) a β-Silicon Group

There are a few noteworthy aspects of this transformation. First, at the outset, we were concerned about competing formation of allyl alcohol and a corresponding fluorosilane, driven by the formation of a stable Si–F bond (Scheme 1).^23^ By ^1^H NMR analysis of the crude product mixtures, however, very little allyl alcohol was produced from any of the silanes contained in Table 1. Other minor byproducts observed were small amounts of the corresponding diol and aldehyde, the latter presumably via a Meinwald-type rearrangement.^24,25^ Second, this fluoride opening of epoxysilanes occurs at room temperature. Other reports of fluorohydrin synthesis by epoxide opening with HF·Et_3_N generally require heating in order to achieve high conversion.^26,27^ For instance, conversion of cyclohexene oxide (**8**) to the corresponding fluorohydrin with HF·Et_3_N required 155 °C for 5 h.^28^ Similarly, Adam and co-workers reported that the opening of glycidyl ether epoxide **9** with HF·Et_3_N needed a high temperature (110 °C), which gave a 53% yield of the corresponding fluorohydrins **10** as a 4:1 mixture of regioisomers.^29^

**Figure 1:**
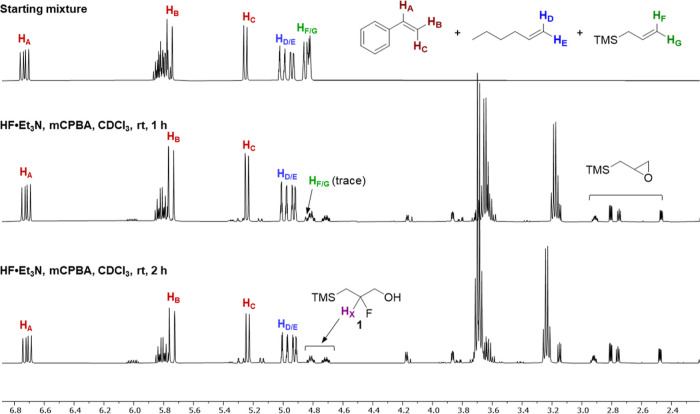
Results from the treatment of a ∼1:1:1 mixture of styrene, 1-hexene, and allyltrimethylsilane (top spectrum) with HF·Et_3_N and mCPBA in CDCl_3_. By ^1^H NMR, allyltrimethylsilane is clearly more reactive to these conditions, being nearly completely consumed after 1 h, whereas signals belonging to styrene (H_A–C_) and 1-hexene (H_D,E_) persist.

We attribute the rate acceleration for epoxysilane openings with HF·Et_3_N, along with complete regioselectivity, to the β-silicon effect,^30^ a well-established phenomenon underpinning rate enhancements observed for other reactions involving cationic intermediates with a silicon group at the β-position.^31^ To benchmark the β-silicon effect in these reactions, a ∼1:1:1 mixture of allyltrimethylsilane, 1-hexene, and styrene was treated with HF·Et_3_N and mCPBA in CDCl_3_, and progress was monitored by ^1^H NMR. As shown in Figure 1, after 1 h at room temperature, allyltrimethylsilane has been essentially completely consumed, converted to fluorohydrin **1** and epoxide precursor. After 2 h, no signals belonging to allyltrimethylsilane are detectable by NMR, yet significant quantities of unreacted 1-hexene and styrene remain.

**Scheme 2:**
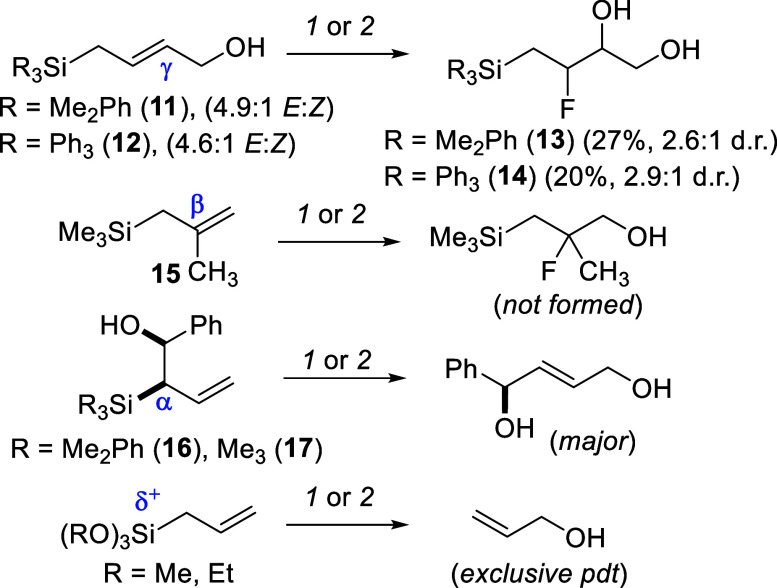
Attempted Fluorohydrin Synthesis from Allylsilanes Substituted at the α-, β-, and γ-Positions as Well as Alkoxy-Substituted Allylsilanes Reagents and conditions: (1) mCPBA, HF·Et_3_N, DCM, rt. (2) (a) TBAHS, K_2_CO_3_, oxone, DMM-MeCN. (b) HF·Et_3_N, DCM, rt.

In the interest of expanding to enantioenriched products by Sharpless epoxidation,^32^ we also prepared and tested conversion of allylic alcohols **11** and **12**(33) (Scheme 2). Use of either of these compounds resulted in low isolated yields of the corresponding fluorohydrins **13** and **14** whether by a two-step or a one-pot procedure (max. 27 and 20%, respectively) due to competing elimination and formation of an Si–F species (evidenced by a singlet at ∼170 ppm in the ^19^F NMR spectrum).^34^ It is worth noting that the d.r. of the products did not match the E/Z ratio of the starting allylsilanes, which has mechanistic implications (vide infra). Substitution of the other alkene carbon (β) or the allylic (α) position was similarly detrimental to fluorohydrin formation. The reaction of methallyltrimethylsilane (**15**) gave mostly unreacted starting material along with smaller amounts of unidentified byproducts. α-Hydroxy allylsilanes **16**(35) and **17**(36) as well as allyltrimethoxy- and allyltriethoxysilane produced exclusively elimination products. While the exact reason for the failure of these substrates is not yet known, it could be that additional substitution sterically hinders epoxide opening, thereby directing fluoride instead to attack silicon (potentially forming the fluorosilicate complex^37,38^) and promoting elimination. Along these lines, the use of more electrophilic-at-silicon (δ^+^) allylalkoxysilanes might similarly favor Si–F rather than C–F bond formation, leading to elimination over fluorohydrin formation.

**Scheme 3:**
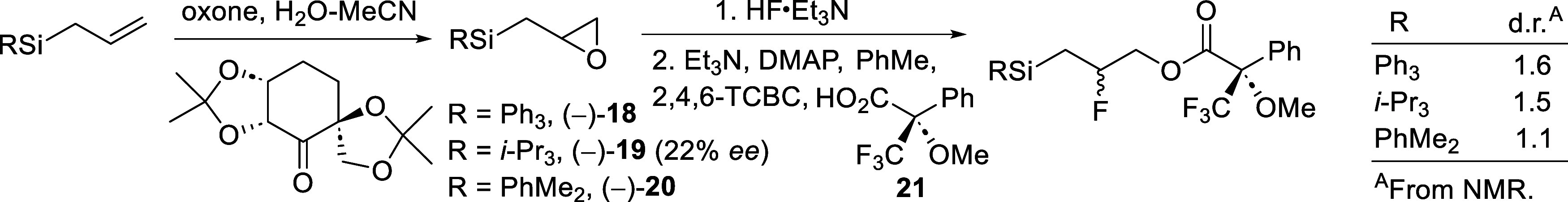
Preparation of Enantioenriched Epoxysilanes and Their Corresponding Fluorohydrin with Assessment of Fluorohydrin Enantiopurity by Conversion to the Mosher Ester Derivative

To better understand the mechanism of this transformation, we attempted to prepare enantioenriched epoxysilanes **18**–**20** featuring differing silicon substitution by Shi epoxidation^39^ of the corresponding allylsilanes (Scheme 3). Like we observed for the racemic sequence, when using allyltriphenylsilane, the Shi epoxidation was slower than the other differently substituted allylsilanes. Nonetheless, good yields of triphenylsilyl epoxide **18** could be achieved using a slightly more concentrated reaction mixture and extended reaction times. A comparison of the measured optical activity for triisopropyl silyl epoxide **19** with that previously reported^39^ indicated that **18** was obtained as a 61:39 mixture of enantiomers (22% ee), in line with previously obtained values for the same transformation.^39^ Treatment of **18**–**20** with HF·Et_3_N followed by esterification with (*S*)-methoxy-α-(trifluoromethyl)phenylacetic acid (**21**) allowed for an assessment of fluorohydrin enantiopurity by ^1^H NMR analysis. The enantiopurity of the resulting triisipropylsilyl fluorohydrin was determined to be 1.5:1, consistent with the ee value measured for the starting epoxide and epoxide opening via an S_N_2-type process. Enantiopurities of the triphenylsilyl and dimethylphenylsilyl fluorohydrins, however, were found to be different (1.6:1 and 1.1:1). Unfortunately, allyltriphenyl- and allyldimethylphenylsilane were not included in Shi’s report,^39^ nor was optical rotation data available elsewhere from which the ee of the starting epoxides could be determined. Assuming a similarly low ee for **18** and **20** as that obtained for **19** (22%) by Shi’s method, we were concerned that detecting minor differences between the compounds in their conversion to the corresponding fluorohydrins could be challenging. We therefore set out to examine alternative methods for the preparation of enantioenriched epoxysilanes to be used for understanding the mechanism of fluorohydrin synthesis.

**Scheme 4:**
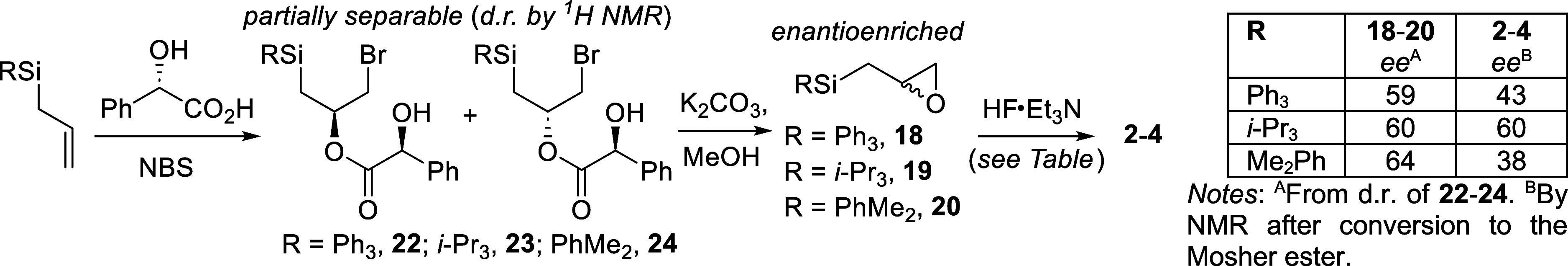
Synthesis of Enantioenriched Epoxides **18–20** and Fluorohydrins **2–4** and a Comparison of Their Enantiopurity (ee)

After screening several methods (e.g., Jacobsen resolution^40^ and Sharpless dihydroxylation^41^), ultimately, we settled on Taber’s alkene bromomandelation chemistry^42^ for generating epoxysilanes **18**–**20** with an appreciable amount of enantioenrichment (Scheme 4). This protocol involves formation of diastereomeric bromomandelate adducts (**22–24**) that are (partially) separable by chromatography on silica. The diastereomeric purity (d.r.) of isolated fractions could be determined by NMR, which translated directly to the enantiopurity of the resulting epoxysilanes **18–20** formed upon treatment with potassium carbonate in methanol.^42^ Comparing the enantiopurity of the starting epoxysilane **18–20** (via the d.r. of the corresponding bromomandelate starting material **22–24** from NMR) to the enantiopurity of the corresponding fluorohydrin **2–4** (via the d.r. of the Mosher ester derivative by NMR), again differences were observed depending on substitution at silicon.

According to our analysis, the ee of the triisopropylsilyl epoxide **19** was retained in the fluorohydrin product, consistent with our previous results using **19** prepared by Shi’s method^39^ and suggestive of an S_N_2-type epoxide opening. However, an erosion of ee was observed for the other two epoxysilanes tested (from 59 to 43% ee for Ph_3_ and 64% to 38% for Me_2_Ph). The fact that complete loss of enantiopurity, which has been reported for similar transformations of styrenyl systems,^19^ did not occur indicates some S_N_2-type behavior. However, loss in ee suggests contribution of an S_N_1 mechanism for epoxide opening involving a silyl-stabilized cation, which is consistent with the incomplete stereospecificity observed when using predominantly *trans* Me_2_Ph and Ph_2_ allylsilanes **11** and **12** (ref. Scheme 2). It is worth noting that the rates of epoxide opening for the three epoxysilanes tested were different, with the triphenyl- and dimethylphenyl substrates being slower than triisopropyl, perhaps reflective of different mechanisms along the S_N_1/S_N_2-spectrum.^43−48^

**Figure 2:**
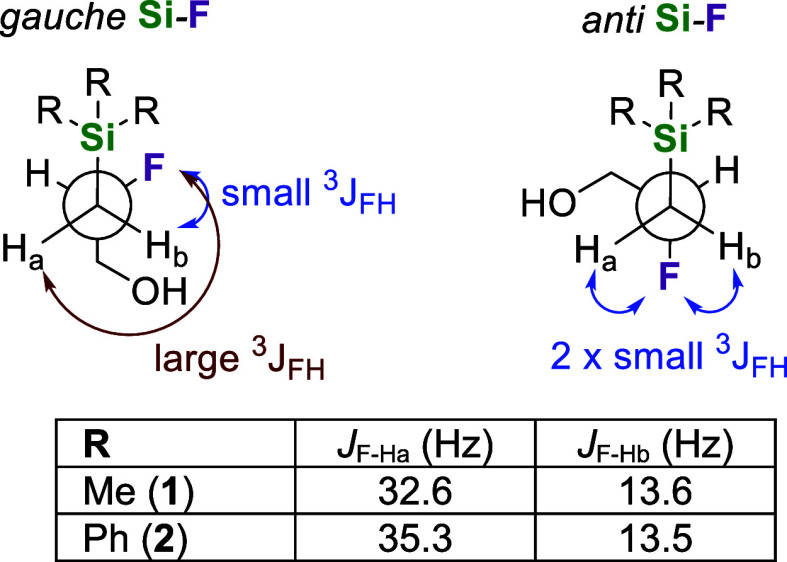
^1^H NMR data for fluorohydrins **1** and **2** showing ^3^*J*_FH_ values consistent with a gauche Si–F conformation.

The fluorohydrin products obtained from this sequence are unique in that the presence of silicon adds an additional possible element of conformational control to the constraints provided by the fluorine gauche effect^49^ associated with the fluorohydrin segment.^50^ Hyperconjugation considerations would suggest a preferred antiperiplanar C–Si and C–F arrangement, with stabilization afforded by overlap between the high energy σ_C–Si_ and the low energy σ*_C_–_F_.^51^ Interestingly, NMR analysis of the different 2-fluoro-3-silylpropan-1-ol products suggested a conserved preference for a gauche C–Si/C–F arrangement (Figure 2). For instance, 3-silyl fluorohydrins **1** and **2** each displayed one large (33–35 Hz) and one small (13.5–13.6 Hz) ^3^J_HF_ coupling constant, consistent with a gauche rather than anti Si–F conformation.

**Figure 3:**
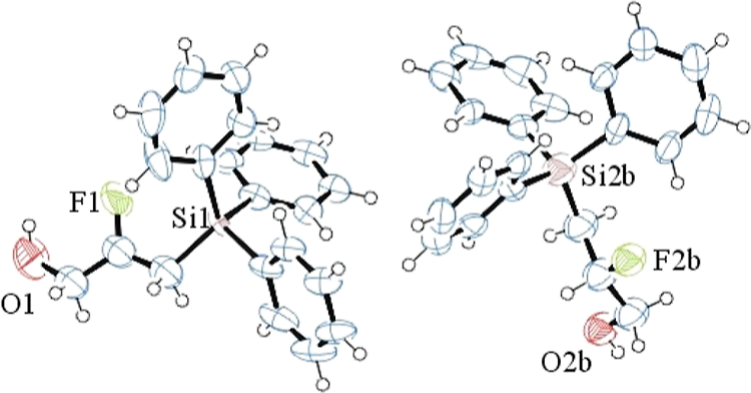
Crystal structure ORTEP images of triphenylsilyl fluorohydrin **2** with thermal ellipsoids at the 50% probability level. Both structures detected showed proximity between Si and F; however, the OH group was found to be either gauche (left structure; *Ø*_F–OH_ = 18°) or anti (right structure; *Ø*_F–OH_ = 179°).

The triphenylsilyl fluorohydrin **2** proved to be crystalline, and suitable crystals were able to be grown for analysis by X-ray diffraction. Two independent structures were observed, both triple-disordered referring to uncertainty in the *x*,*y*,*z* planes as to where the crystal resides within the unit cell (Figure 3). In one of the solved structures, the F and OH groups are oriented gauche (dihedral angle (*Ø*_F–OH_) = 18°), consistent with other fluorohydrin compounds.^50^ The other, however, shows these two groups oriented anti (*Ø*_F–OH_ = 179°), perhaps influenced by the sterically large SiPh_3_. Both structures display proximity between Si and F (*Ø*_Si–F_ = 6 and 38°). The dominant conformational bias in this system, therefore, appears to be a silicon–fluorine “gauche” effect.

**Figure 4:**
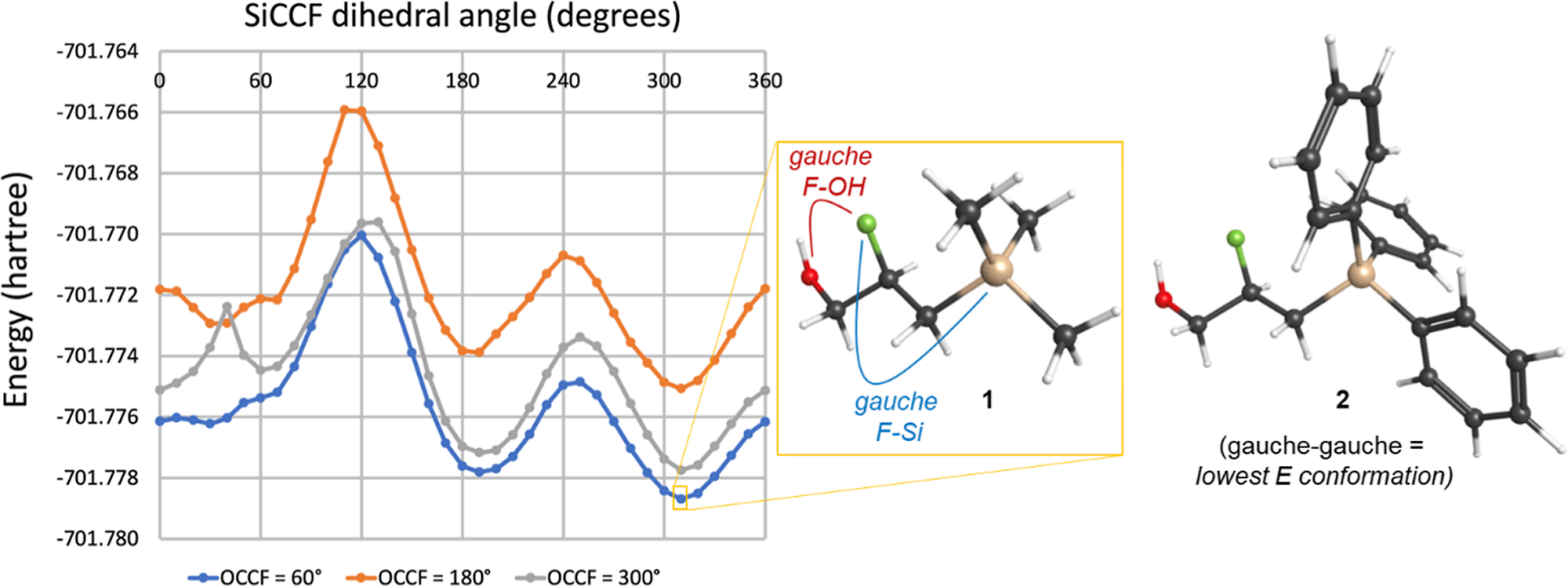
Results from DFT-PBE conformational analysis of 2-fluoro-3-(trimethylsilyl)propan-1-ol (**4**). For three different F–OH orientations (*Ø*_F–OH_ = 60, 180, or 300°), the lowest energy conformer contained a gauche Si–F arrangement (*Ø*_Si–F_ ∼ 310°).

To better understand this apparent silicon–fluorine gauche effect and its connection to the F–OH orientation in these molecules, conformational analysis by density functional theory (DFT) was performed (Figure 4). Using both PBE and B3LYP methods, the lowest energy conformation calculated for trimethylsilyl fluorohydrin **1** had the Si and F groups approximately gauche (Si–C–C–F *Ø* ∼ 310°) irrespective of the F–OH orientation (*Ø*_F–OH_ = 60, 180, or 300°). The absolute energy minimum had both Si–F and F–OH groups gauche. This was also true for the triphenylsilyl fluorohydrin **2** (calculated energy minimum at *Ø*_Si–F_ ∼ 50° for *Ø*_F–OH_ = 60°), which is in fairly good agreement with results from X-ray analysis (e.g., *Ø*_Si–F_ = 38°) when accounting for potential crystal forces^52,53^ and the small energy differences calculated between the different conformations (Δ*E* ∼ 2 kJ/mol for *Ø*_Si–F_ 50° vs *Ø*_Si–F_ 60°). Our working hypothesis is that the gauche–gauche conformation has the lowest energy due to a combination of hyperconjugation (e.g., σ_C–H_ → σ_C–F_*)^54^ and electrostatics (e.g., Si^δ+^ → F^δ−^).^55^

**Scheme 5:**
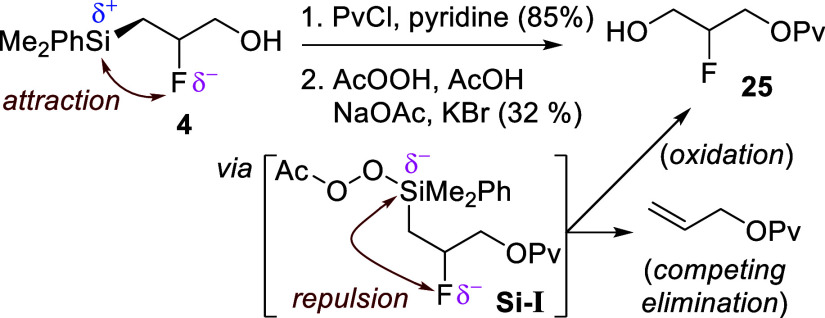
Synthesis of an End-Group-Differentiated Fluoroglycerol Analogue **25**

Given not only the value of fluorine-containing compounds for drug discovery^1−7^ but also an emerging interest in organosilanes for this purpose,^56^ silicon-substituted fluorohydrins could present a novel platform for designing conformationally restricted biologically active structures. Alternatively, oxidative desilylation would generate 2-fluoro-1,3-propanediols, which have proven to be useful for studying enzymatic reactions involving glycerol^57,58^ as well as starting points to access fluorinated carbohydrate analogues of medicinal value.^59^ To that end, Tamao–Fleming oxidation^60^ of the 3-silyl fluorohydrin products was investigated. Ultimately it was found that after acylation of dimethylphenylsilyl fluorihydrin **4** with pivaloyl chloride (PvCl) and treatment of the resulting pivaloyl ester (**4-piv**) with peracetic acid (AcOOH) in the presence of sodium acetate (NaOAc) and potassium bromide,^61^ the differentiated 2-fluoro-1,3-propanediol **25** could be obtained (Scheme 5). The low yield in this case (32%), and observed in other attempted Tamao–Fleming oxidations of 2-fluoro-3-silylpropan-1-ols, was caused by competing elimination, presumably via a mechanism involving intermediate **Si–I**. If electrostatics (i.e., attraction between Si^δ+^ and F^δ−^) is what controls the observed Si–F gauche effect in the neutral compound, upon activation of silicon to form the corresponding silicate (Si^δ−^) during Tamao–Fleming oxidation, this attraction would then become a repulsion. As a result, **Si–I** would adopt an antiperiplanar Si–F conformation, facilitating elimination and causing low yields of the oxidatively cleaved products. While extensive optimization of this reaction has yet to be performed, the value of 2-fluoro-3-silylpropan-1-ols as synthetic intermediates might therefore be maximized by retaining silicon and targeting functional organosilanes.

## Conclusions

In summary, various allylsilanes can be converted to the corresponding 2-fluoro-3-silylpropan-1-ols in good yield and excellent regioselectivity by epoxidation followed by epoxide opening with HF·Et_3_N. Compared with other fluorohydrin syntheses by epoxide opening with HF·Et_3_N, formation of these silicon-substituted fluorohydrins occurs more readily (e.g., at room temperature) and with higher regioselectivity that we attribute to a β-silyl effect. Reactions tended to be slower with phenyl-substituted silanes, which could be due to differences in the mechanism, which is supported by data from reactions using enantioenriched epoxysilanes. The volatility of some intermediate epoxysilanes prompted us to investigate a one-pot epoxidation/epoxide opening reaction using a combination of mCPBA and HF·Et_3_N. Yields for this one-pot procedure were generally in the same range as the overall yield from a two-step process involving epoxidation with in situ-generated oxone followed by treatment with HF·Et_3_N. However, the use of mCPBA generally gave small amounts of the 3-chlorobenzoate adduct resulting from 3-chlorobenzoic acid epoxide opening. For this reason, our preferred method for substrates with suitably low volatility remains the two-step sequence. Analysis of the 3-silylfluorohydrin products by NMR, X-ray diffraction, and theory points to a preferred conformation wherein Si and F are proximal. Contrary to other fluorine gauche effects based on hyperconjugative interactions, we hypothesize that the conformational bias of 3-silylfluorohydrins is driven by an electrostatic attraction between Si^δ+^ and F^δ−^. Efforts are currently focused on further transformations of these compounds to access valuable fluorine- and/or silicon-containing target structures.

## Experimental Section

### General Information

All reactions were carried out in vessels open to air at ambient conditions unless otherwise specified. Dry solvents used were prepared by passing the solvent through a column of activated alumina under nitrogen immediately prior to use. All reagents were purchased and used as received unless mentioned otherwise. Thin-layer chromatography (TLC) analysis used 0.25 mm silica layer fluorescence UV_254_ plates. Column chromatography: silica gel (230–400 mesh). IR: FT-IR with single-bounce diamond ATR. NMR: spectra were recorded on a 500 MHz spectrometer in CDCl_3_; chemical shifts (δ) are given in ppm, coupling constants (*J*) in Hz. Solvent signals were used as references (CDCl_3_: δ_c_ ≡ 77.0 ppm; residual CHCl_3_ in CDCl_3_: δ_H_ ≡ 7.26 ppm). HRMS: quadrupole time-of-flight LC–MS with electrospray ionization (ESI positive and negative). X-ray crystallography: samples were prepared by slow diffusion (pentane into MTBE) at 0 °C. A colorless needle, measuring 0.375 × 0.100 × 0.080 mm^3^, was mounted on a loop with oil. Data were collected at −173 °C on a single-crystal X-ray diffractometer (Mo-radiation) equipped with an X-ray optical collimator.

### General Experimental Procedures

#### General Procedure A1: Oxone Epoxidation

Adapted from Frohn et al.:^18^ To a vigorously stirred mixture of allylsilane (1.0 mmol) and *tert*-butyl ammonium hydrogen sulfate (0.014 g, 0.04 mmol) in acetonitrile–dimethoxymethane (2:1, 8 mL), acetone (2.2 mL, 30 mmol), and aq K_2_CO_3_ (0.1 M, 2 mL) were added oxone (3 mmol, in 8 mL of 4 × 10^–4^ M EDTA solution) and aq K_2_CO_3_ (1.66 M, 8 mL) simultaneously via a syringe pump over the indicated time. The reaction was extracted with hexanes (3 × 20 mL), and the combined extracts were washed with brine and dried over MgSO_4_ before removing the solvent on a rotary evaporator. The crude epoxide was then used directly in the next reaction without further purification.

#### General Procedure A2: HF·Et_3_N Epoxide Opening

A Teflon vial was charged with epoxysilane (1 equiv) and DCM (to make a 0.05–0.1 M solution). The solution was stirred, and HF·Et_3_N (5 equiv) was added dropwise via a syringe. The reaction vessel was sealed with a Teflon screw cap, and the mixture was stirred over the indicated time at room temperature. The reaction was then quenched by pouring into a beaker containing satd. aq NaHCO_3_ (75 mL) and allowed to stir until no evolution of CO_2_ was observed. The mixture was transferred to a separatory funnel and extracted with DCM (3 × 25 mL). The combined organic extracts were dried over MgSO_4_, filtered, and concentrated on a rotary evaporator. The crude product was then purified by column chromatography on silica.

#### General Procedure B: One-Pot Epoxidation/Epoxide Opening

Adapted from Sedgwick et al.:^19^ A Teflon vial was charged with mCPBA (1.3 equiv) and DCM (to make a 0.0625–0.1 M solution) and stirred until the mCPBA had dissolved. With stirring, HF·Et_3_N (5–7 equiv) was then added followed immediately by the allylsilane (1 equiv) via a syringe. The reaction vessel was sealed with a Teflon screw cap, and the mixture was stirred for the indicated time at room temperature. The reaction was quenched by pouring into a beaker containing satd. aq NaHCO_3_ (75 mL) and allowed to stir until no evolution of CO_2_ was observed. The mixture was transferred to a separatory funnel and extracted with CH_2_Cl_2_ (3 × 25 mL). The combined organic extracts were dried over MgSO_4_, filtered, and concentrated on a rotary evaporator. The crude product was then purified by column chromatography on silica.

#### General Procedure C: Shi Epoxidation/Epoxide Opening

Adapted from Wang et al.:^39^ To a mixture of the allylsilane (1.0 mmol) and *tert*-butyl ammonium hydrogen sulfate (0.014 g, 0.04 mmol) in acetonitrile (15 mL), the Shi catalyst (78 mg, 0.3 mmol) was added as a buffered solution (10 mL, 0.05 M Na_2_B_4_O_7_·10H_2_O in 4 × 10^–4^ M aq Na_2_ (EDTA)). With vigorous stirring, a solution of oxone (6.5 mL, 0.2 M in 4 × 10^–4^ M Na_2_ (EDTA)) and aq K_2_CO_3_ (6.5 mL, 0.9 M) were added simultaneously via syringe pump over the indicated time. Upon completion, the reaction mixture was extracted with hexanes (3 × 20 mL). The combined organic extracts were washed with brine, dried over MgSO_4_, and filtered before removing the solvent on a rotary evaporator. The resulting epoxide product was converted to the corresponding fluorohydrin without further purification using procedure A2.

#### General Procedure D: Bromomandelation/Epoxide Formation/Epoxide Opening

Adapted from Taber and Liang:^42^ To a solution of (*S*)-mandelic acid (2.3 equiv) and 2,6-lutidine (2.6 equiv) in dry DCM (to make a 0.25 M solution) under N_2_, allylsilane was added and the flask was placed in a room temperature water bath before adding NBS (1.5 equiv). The mixture was stirred for 4–18 h before being quenched with sat. NaHCO_3_ (15 mL) and extracted with DCM (2 × 15 mL). The combined organic extracts were dried over MgSO_4_, filtered, and concentrated on a rotary evaporator. The crude product was purified by chromatography on silica (10:1 to 4:1 to 1:1 Hex/EtoAc) to yield diastereomerically enriched fractions of the bromomandelate adduct that were treated separately with K_2_CO_3_ (5.0 equiv) in MeOH (to make a 0.1 M solution). The reaction was stirred until completion by TLC (∼20–30 min). MeOH was then removed using a rotary evaporator, and the resulting residue was dissolved in MTBE (25 mL) and washed with aq NH_4_Cl (15 mL) and brine (15 mL). The organic phase was dried over MgSO_4_, filtered, and concentrated on a rotary evaporator. The resulting epoxide product was converted to the corresponding fluorohydrin without further purification using procedure A2.

##### 2-Fluoro-3-(trimethylsilyl)propan-1-ol (**1**)

The product was obtained from allyltrimethylsilane (114 mg, 1 mmol) following procedure B using HF·Et_3_N (0.27 mL, 5 mmol, 5 equiv) and DCM (16 mL) and stirring for 1 h. The crude product was purified by column chromatography on silica (4:1 hexanes/ethyl acetate, *R*_f_ ∼ 0.34) to yield silyl fluorohydrin **4** as a colorless liquid (99 mg, 0.65 mmol, 65%).

IR (ATR): 3295, 3054, 2987, 2873, 1515, 1128, 1058, 703, 697 cm^–1^. ^1^H NMR (500 MHz, CDCl_3_): δ 4.79 (ddtd, *J* = 50.1, 8.9, 6.4, 2.7 Hz, 1H), 3.74–3.59 (m, 2H), 1.09 (ddd, *J* = 14.5, 13.6, 8.7 Hz, 1H), 0.89 (ddd, *J* = 35.2, 14.5, 6.3 Hz, 1H), 0.09 (d, *J* = 0.8 Hz, 8H). ^13^C{^1^H} NMR (126 MHz, CDCl_3_): δ 93.7 (d, *J* = 166.2 Hz), 67.3 (d, *J* = 22.8 Hz), 19.8 (d, *J* = 22.6 Hz), −1.0. ^19^F NMR (470 MHz, CDCl_3_): δ −176.05 (ddddd, *J* = 49.3, 34.6, 28.7, 20.4, 13.7 Hz). HRMS (ESI+) calcd for C_6_H_15_FNaOSi (M + Na), 173.0774; found, 173.0770.

##### 2-Fluoro-3-(triphenylsilyl)propan-1-ol (**2**)

The product was obtained from allyltriphenylsilane (301 mg, 1 mmol) following procedure A1 and stirring for 16 h. The crude epoxide was taken directly into procedure A2 without purification using HF·Et_3_N (0.06 mL, 1 mmol, 5 equiv) and DCM (2.5 mL) and stirring for 72 h. The crude product was purified by column chromatography on silica (4:1 hexanes/ethyl acetate, *R*_f_ ∼ 0.2) to yield silyl fluorohydrin **2** as a white solid (54 mg, 0.16 mmol, 65%).

IR (ATR): 3295, 3063, 2919, 2848, 1425, 1108, 1058, 703, 697 cm^–1^. ^1^H NMR (500 MHz, CDCl_3_): δ 7.56–7.53 (m, 5H), 7.44–7.36 (m, 10H), 4.92–4.76 (ddtd, *J* = 49.2, 8.4, 6.4, 2.9 Hz, 1H), 3.67–3.51 (m, 2H), 2.01 (ddd, *J* = 15.1, 13.6, 8.2 Hz, 1H), 1.74 (ddd, *J* = 32.6, 14.8, 6.4 Hz, 1H). ^13^C{^1^H} NMR (126 MHz, CDCl_3_): δ 135.6, 134.0, 129.8, 128.1, 92.9 (d, *J* = 168.3 Hz), 66.9 (d, *J* = 22.6 Hz), 17.0 (d, *J* = 22.3 Hz). ^19^F NMR (470 MHz, CDCl_3_): δ −173.21 to −173.55 (m). HRMS (ESI+) calcd for C_21_H_21_FNaOSi (M + Na), 359.1243; found, 359.1244.

##### 2-Fluoro-3-(triisopropylsilyl)propan-1-ol (**3**)

The product was obtained from allyltriisopropylsilane (198 mg, 1 mmol) following procedure A1 and stirring for 4 h. This was then taken directly into procedure A2 without purification using HF·Et_3_N (0.08 mL, 1.4 mmol, 5 equiv) and DCM (5.6 mL) and stirring for 1.5 h. The crude product was purified by column chromatography on silica (4:1 hexanes/ethyl acetate, *R*_f_ ∼ 0.4) to yield silyl fluorohydrin **3** as a colorless liquid (64 mg, 0.27 mmol, 92%).

IR (ATR): 3285, 3024, 2954, 2895, 1614, 1318, 1123, 1020, 763 cm^–1^. ^1^H NMR (500 MHz, CDCl_3_): δ 4.82 (ddddd, *J* = 49.7, 9.8, 7.2, 4.9, 2.7 Hz, 1H), 3.76–3.58 (m, 2H), 1.15–1.01 (m, 23H), 0.86 (ddd, *J* = 41.6, 14.9, 4.8 Hz, 1H). ^13^C{^1^H} NMR (126 MHz, CDCl_3_): δ 93.3 (d, *J* = 166.2 Hz), 67.8 (d, *J* = 23.5 Hz), 18.72, 18.69, 12.2 (d, *J* = 25.1 Hz), 11.3. ^19^F NMR (470 MHz, CDCl_3_): δ −175.25 to −175.63 (m). HRMS (ESI+) calcd for C_12_H_27_FNaOSi (M + Na), 257.1713; found, 257.1709.

##### 3-(Dimethyl(phenyl)silyl)-2-fluoropropan-1-ol (**4**)

The product was obtained from allyldimethyl(phenyl)silane (176 mg, 1 mmol) following procedure A1 using HF·Et_3_N (0.27 mL, 5 mmol 5 equiv), DCM (20 mL)and stirring for 4 h. This was then taken directly into procedure A2 without purification. The crude product was purified by column chromatography on silica (4:1 hexanes/ethyl acetate, *R*_f_ ∼ 0.2) to yield **4** as a colorless liquid (154 mg, 0.724 mmol, 72%).

IR (ATR): 3250, 3015, 2987, 2867, 1610, 1574, 1435, 1218, 1090, 785, 767 cm^–1^. cm^–1^. ^1^H NMR (500 MHz, CDCl_3_): δ 7.56–7.48 (m, 2H), 7.42–7.32 (m, 3H), 4.81–4.65 (ddtd, *J* = 50.0, 9.0, 6.4, 2.8 Hz, 1H), 3.67–3.51 (m, 2H), 1.81 (s, 1H), 1.32 (ddd, *J* = 14.6, 13.3, 8.7 Hz, 1H), 1.12 (ddd, *J* = 35.1, 14.6, 6.1 Hz, 1H), 0.38 (s, 3H), 0.36 (s, 3H). ^13^C{^1^H} NMR (126 MHz, CDCl_3_): δ 138.0, 133.5, 129.3, 128.0, 93.4 (d, *J* = 166.6 Hz), 67.1 (d, *J* = 22.8 Hz), 19.3 (d, *J* = 22.7 Hz), −2.1, −2.6. ^19^F NMR (470 MHz, CDCl_3_): δ −175.73 (ddddd, *J* = 49.0, 34.7, 28.0, 21.1, 13.3 Hz). HRMS (ESI+) calcd for C_11_H_17_FNaOSi (M + Na), 235.0930; found, 235.0924.

##### 3-(Allyldiphenylsilyl)-2-fluoropropan-1-ol (**5**)

The product was obtained from diallyldiphenylsilane (238 mg, 0.9 mmol) following procedure B using HF·Et_3_N (0.27 mL, 4.5 mmol, 5 equiv) and DCM (9 mL) and stirring for 10 h. The crude product was purified by column chromatography on silica (4:1 hexanes/ethyl acetate, *R*_f_ ∼ 0.17) to yield silyl fluorohydrin **5** as a colorless liquid (143 mg, 0.48 mmol, 53%).

IR (ATR): 3324, 3070, 3047, 2927, 2869, 1629, 1427, 1106, 900, 844, 696 cm^–1^. ^1^H NMR (500 MHz, CDCl_3_): δ 7.54 (ddt, *J* = 12.4, 7.9, 1.5 Hz, 4H), 7.39 (dddd, *J* = 13.5, 8.0, 6.8, 4.7 Hz, 6H), 5.79 (dddd, *J* = 18.2, 15.8, 7.9, 1.2 Hz, 1H), 4.95 (dt, *J* = 16.9, 1.6 Hz, 1H), 4.91 (ddd, *J* = 10.0, 2.2, 1.0 Hz, 1H), 4.85–4.69 (ddtd, *J* = 49.2, 9.0, 6.0, 3.0 Hz, 1H), 3.66–3.52 (m, 2H), 2.20 (d, *J* = 7.9 Hz, 2H), 1.72 (ddd, *J* = 14.9, 12.5, 8.8 Hz, 1H), 1.44 (ddd, *J* = 35.5, 14.8, 5.9 Hz, 1H). ^13^C{^1^H} NMR (126 MHz, CDCl_3_): δ 134.94, 134.90, 134.4, 134.2, 133.5, 129.77, 129.7, 128.1, 128.0, 115.1, 92.9 (d, *J* = 167.2 Hz), 67.0 (d, *J* = 22.9 Hz), 21.1, 15.8 (d, *J* = 23.2 Hz). ^19^F NMR (470 MHz, CDCl_3_): δ −174.96 to −175.39 (m). HRMS (ESI+) calcd for C_18_H_21_FNaOSi (M + Na), 323.1243; found, 323.1243.

##### 3-(Allyldimethylsilyl)-2-fluoropropan-1-ol (**6**)

The product was obtained from diallyldimethylsilane (70.2 mg, 0.5 mmol) following procedure B using HF·Et_3_N (0.14 mL, 2.5 mmol, 5 equiv) and DCM (8 mL) and stirring for 1 h. The crude product was purified by column chromatography on silica (4:1 hexanes/ethyl acetate, *R*_f_ ∼ 0.34) to yield silyl fluorohydrin **6** as a colorless liquid (50 mg, 0.28 mmol, 56%).

IR (ATR): 3325, 3047, 2928, 2873, 1629, 1457, 1130, 901, 845, 697 cm^–1^. ^1^H NMR (500 MHz, CDCl_3_): δ 5.77 (ddt, *J* = 16.6, 10.3, 8.1 Hz, 1H), 4.91–4.68 (m, 3H), 3.74–3.56 (m, 2H), 1.58 (dt, *J* = 8.1, 1.3 Hz, 2H), 1.09 (ddd, *J* = 14.6, 12.7, 9.1 Hz, 1H), 0.88 (ddd, *J* = 37.2, 14.6, 5.9 Hz, 1H), 0.08 (d, *J* = 0.9 Hz, 6H). ^13^C{^1^H} NMR (126 MHz, CDCl_3_): δ 134.4, 113.5, 93.5 (d, *J* = 165.9 Hz), 67.3 (d, *J* = 23.1 Hz), 23.6, 17.9 (d, *J* = 23.6 Hz), −3.0, −3.1. ^19^F NMR (470 MHz, CDCl_3_): δ −176.55 (ddddd, *J* = 49.5, 37.3, 28.5, 20.2, 12.6 Hz). HRMS (ESI+) calcd for C_8_H_17_FNaOSi (M + Na), 199.0925; found, 199.0922.

##### 3-((Bromomethyl)dimethylsilyl)-2-fluoropropan-1-ol (**7**)

The product was obtained from allyl(bromomethyl) dimethylsilane (193 mg, 1 mmol) following procedure A1 and stirring for 4 h. This was then taken directly into procedure A2 without purification using HF·Et_3_N (0.13 mL, 2.5 mmol, 5 equiv) and DCM (10 mL) and stirring for 2 h. The crude product was purified by column chromatography on silica (4:1 hexanes/ethyl acetate, *R*_f_ ∼ 0.4) to yield silyl fluorohydrin **7** as a colorless liquid (69 mg, 0.30 mmol, 60%).

IR (ATR): 3334, 3057, 2978, 2865, 1615, 1511, 1425, 1038, 930, 854, 698 cm^–1^. ^1^H NMR (500 MHz, CDCl_3_): δ 4.89–4.72 (ddddd, *J* = 49.5, 9.6, 6.7, 5.0, 2.7 Hz, 1H), 3.75–3.58 (m, 2H), 2.52 (d, *J* = 2.5 Hz, 2H), 1.22 (ddd, *J* = 14.8, 11.1, 9.8 Hz, 1H), 1.02 (ddd, *J* = 40.6, 14.9, 5.0 Hz, 1H), 0.22 (s, 6H). ^13^C{^1^H} NMR (126 MHz, CDCl_3_): δ 93.2 (d, *J* = 166.2 Hz), 67.4 (d, *J* = 24.4 Hz), 17.5 (d, *J* = 25.1 Hz), 17.1, −3.21, −3.23. ^19^F NMR (470 MHz, CDCl_3_): δ −177.82 (dtdd, *J* = 49.8, 40.3, 20.8, 11.2 Hz). HRMS (ESI+) calcd for C_6_H_14_BrFNaOSi (M + Na), 250.9879; found, 250.9884.

##### 4-(Dimethyl(phenyl)silyl)-3-fluorobutane-1,2-diol (**13**)

The product was obtained from 4-(dimethyl(phenyl)silyl)but-2-en-1-ol (**11**)^32^ (128 mg, 0.62 mmol) following procedure B using HF·Et_3_N (0.23 mL, 4.3 mmol, 7 equiv) and DCM (6 mL) and stirring for 4 h. The crude product was purified by column chromatography on silica (1:1 hexanes/ethyl acetate, *R*_f_ ∼ 0.1) to yield silyl fluorohydrin **13** as a colorless liquid (41 mg, 0.16 mmol, 27%).

##### Spectral Data for the Mixture of Diastereomers

IR (ATR): 3465, 3279, 3070, 2956, 2926, 2866, 1612, 1510, 1486, 1388, 1362, 1236, 1185, 992, 765 cm^–1^. ^1^H NMR (500 MHz, CDCl_3_): δ 7.57–7.50 (m, 4H), 7.37 (dtd, *J* = 5.1, 3.8, 1.4 Hz, 6H), 4.75–4.58 (m, 2H), 3.77–3.55 (m, 6H), 1.35 (tt, *J* = 16.3, 10.9 Hz, 2H), 1.25–1.10 (m, 2H), 0.39–0.36 (m, 12H). ^19^F NMR (470 MHz, CDCl_3_): δ −180.18 (tt, *J* = 46.1, 12.3 Hz), −183.53 (tdd, *J* = 46.2, 18.6, 10.3 Hz). Spectral data for the major diastereomer: ^13^C NMR (126 MHz, CDCl_3_): δ 133.5, 129.8, 129.3, 128.0, 93.4 (d, *J* = 166.7 Hz), 75.8 (d, *J* = 20.5 Hz), 63.3 (d, *J* = 6.2 Hz), 19.3 (d, *J* = 25.7 Hz), −2.1, −2.6. HRMS (ESI+) calcd for C_12_H_20_FO_2_Si (M + H), 243.1217; found, 243.1221.

##### 4-((Triphenyl)silyl)-3-fluorobutane-1,2-diol (**14**)

The product was obtained from 4-((triphenyl)silyl)but-2-en-1-ol (**12**)^33^ (225 mg, 0.68 mmol) following procedure B using HF·Et_3_N (0.37 mL, 6.8 mmol, 10 equiv) and DCM (7 mL) and stirring for 16 h. The crude product was purified by column chromatography on silica (1:1 hexanes/ethyl acetate, *R*_f_ ∼ 0.1) to yield silyl fluorohydrin **14** as a colorless liquid (50 mg, 0.14 mmol, 20%).

##### Spectral Data for the Mixture of Diastereomers

IR (ATR): 3239, 3228, 3019, 2867, 1429, 1131, 1121, 907, 840 cm^–1^. ^1^H NMR (500 MHz, CDCl_3_): δ 7.58–7.52 (m, 10H), 7.46–7.34 (m, 19H), 4.85–4.67 (m, 2H), 3.77–3.55 (m, 6H), 2.12–1.96 (m, 2H), 1.91–1.73 (m, 2H). ^19^F NMR (470 MHz, CDCl_3_): δ −177.33 (t, *J* = 45.6 Hz), −181.42 to −181.85 (m). Spectral data for the major diastereomer: ^13^C NMR (126 MHz, CDCl_3_): δ 135.7, 134.1, 129.8, 128.0, 92.8 (d, *J* = 168.3 Hz), 74.8 (d, *J* = 20.0 Hz), 63.5 (d, *J* = 5.5 Hz), 17.1 (d, *J* = 24.9 Hz). HRMS (ESI+) calcd for C_22_H_24_FO_2_Si (M + H), 367.1530; found, 367.1537.

##### Triphenyl(oxiran-2-ylmethyl)silane (**18**)

The product was prepared according to general procedure C, and epoxide **18** (0.205 g, 65%) was isolated as a colorless oil. [α]_D_^25^ −2.35 (*c* 1.0, CH_2_Cl_2_).

Spectral data matched that previously reported:^62^^1^H NMR (500 MHz, CDCl_3_): δ 7.58–7.53 (m, 5H), 7.47–7.36 (m, 10H), 3.17–3.11 (m, 1H), 2.67 (t, *J* = 4.4 Hz, 1H), 2.35 (dd, *J* = 5.0, 2.7 Hz, 1H), 2.12 (dd, *J* = 14.5, 4.5 Hz, 1H), 1.41 (ddd, *J* = 14.5, 8.5, 0.9 Hz, 1H). ^13^C{^1^H} NMR (126 MHz, CDCl_3_): δ 135.6, 134.1, 129.8, 128.1, 50.1, 49.2, 18.4.

##### Triisopropyl(oxiran-2-ylmethyl)silane (**19**)

The product was prepared according to general procedure C, and epoxide **19** (0.176 g, 82%) was isolated as a colorless oil. [α]_D_^25^ −5.66 (*c* 1.45, CH_2_Cl_2_).

Spectral data matched that previously reported:^38^^1^H NMR (500 MHz, CDCl_3_): δ 3.03 (dtd, *J* = 8.7, 4.1, 2.7 Hz, 1H), 2.81 (ddd, *J* = 4.9, 3.8, 1.1 Hz, 1H), 2.47 (dd, *J* = 5.0, 2.8 Hz, 1H), 1.29 (dd, *J* = 14.4, 4.3 Hz, 1H), 1.10–1.04 (m, 21H), 0.63 (dd, *J* = 14.3, 9.0 Hz, 1H). ^13^C{^1^H} NMR (126 MHz, CDCl_3_): δ 50.7, 49.7, 18.7, 14.2, 11.0.

##### Dimethyl(oxiran-2-ylmethyl)(phenyl)silane (**20**)

The product was prepared according to general procedure C, and epoxide **20** (0.187 g, 97%) was isolated as a colorless oil. [α]_D_^25^ −1.08 (*c* 1.0, CH_2_Cl_2_).

IR (ATR): 2987, 2954, 2876, 1545, 1330, 1210, 1097, 765, 698 cm^–1^. ^1^H NMR (500 MHz, CDCl_3_): δ 7.55–7.50 (m, 2H), 7.39–7.35 (m, 3H), 2.98 (dddd, *J* = 8.0, 5.2, 3.9, 2.7 Hz, 1H), 2.73 (ddd, *J* = 4.9, 3.9, 0.9 Hz, 1H), 2.37 (dd, *J* = 5.0, 2.8 Hz, 1H), 1.40 (ddd, *J* = 14.3, 4.9, 0.8 Hz, 1H), 0.85 (dd, *J* = 14.2, 8.2 Hz, 1H), 0.37 (s, 3H), 0.37 (s, 3H). ^13^C{^1^H} NMR (126 MHz, CDCl_3_): δ 138.2, 133.5, 129.3, 127.9, 50.3, 48.7, 20.4, −2.6, −2.6. HRMS (ESI+) calcd for C_11_H_16_NaOSi (M + Na), 215.0868; found, 215.0872.

##### 1-Bromo-3-(triphenylsilyl)propan-2-yl (2*S*)-2-hydroxy-2-phenylacetate (**22**)

The product was obtained from allyltriphenylsilane (1.43 g, 4.8 mmol) following procedure D and stirring for 4 h. Purification by chromatography on silica (10:1 to 4:1 to 1:1 hexanes/ethyl acetate) gave **22** (0.71 g, 28%) as a colorless oil and a partially separable mixture of diastereomers (*R*_f_ diastereomer α = 0.44, *R*_f_ diastereomer β = 0.43 in 4:1 hexanes/ethyl acetate).

IR (ATR): 3234, 3043, 2972, 2846, 1733, 1634, 1525, 1478, 1265, 1038, 872, 738 cm^–1^. ^1^H NMR (500 MHz, CDCl_3_): δ 7.65–7.60 (m, 6H), 7.57–7.53 (m, 4H), 7.53–7.29 (m, 30H), 5.38–5.30 (m, 1H), 5.22 (dddd, *J* = 7.9, 6.6, 5.0, 3.8 Hz, 1H), 5.04 (d, *J* = 5.5 Hz, 1H), 4.50 (d, *J* = 5.1 Hz, 1H), 3.51 (dd, *J* = 11.1, 3.8 Hz, 1H), 3.29 (dd, *J* = 10.8, 4.8 Hz, 1H), 3.27 (dd, *J* = 11.1, 5.0 Hz, 1H), 3.22 (dd, *J* = 10.8, 4.7 Hz, 1H), 3.16 (d, *J* = 5.4 Hz, 1H), 3.07 (d, *J* = 5.8 Hz, 1H), 2.12 (dd, *J* = 15.1, 9.3 Hz, 1H), 2.05 (dd, *J* = 15.0, 6.6 Hz, 1H), 1.95 (dd, *J* = 15.0, 5.1 Hz, 1H), 1.87 (dd, *J* = 15.0, 7.9 Hz, 1H). ^13^C{^1^H} NMR (126 MHz, CDCl_3_): δ 172.8, 172.4, 137.9, 135.7, 135.6, 133.9, 133.6, 130.1, 130.0, 128.6, 128.52, 128.46, 128.27, 128.2, 126.8, 126.7, 73.30, 73.28, 72.5, 72.4, 36.2, 36.1, 18.2, 18.1. HRMS (ESI+) calcd for C_29_H_28_BrO_3_Si (M + H), 531.0991; found, 531.0993.

##### 1-Bromo-3-(triisopropylsilyl)propan-2-yl (2*S*)-2-hydroxy-2-phenylacetate (**23**)

The product was obtained from allyltriisopropylsilane (1.2 mL, 5.0 mmol) following procedure D and stirring for 4 h. Purification by chromatography on silica (10:1 to 4:1 to 1:1 hexanes/ethyl acetate) gave **23** (1.04 g, 48%) as a colorless oil and a partially separable mixture of diastereomers (*R*_f_ diastereomer α = 0.44, *R*_f_ diastereomer β = 0.38 in 4:1 hexanes/ethyl acetate).

IR (ATR): 3240, 3057, 2978, 2826, 1735, 1623, 1515, 1407, 1130, 1062, 864, 729 cm^–1^. ^1^H NMR (500 MHz, CDCl_3_): δ 7.48–7.32 (m, 10H), 5.31–5.20 (m, 2H), 5.16 (d, *J* = 5.5 Hz, 2H), 3.65 (dd, *J* = 10.7, 4.3 Hz, 1H), 3.49 (dd, *J* = 10.8, 5.0 Hz, 1H), 3.44 (d, *J* = 5.5 Hz, 2H), 3.34 (dd, *J* = 10.8, 4.7 Hz, 1H), 3.29 (dd, *J* = 10.7, 4.9 Hz, 1H), 1.16–1.04 (m, 3H), 0.97 (dd, *J* = 15.1, 6.0 Hz, 1H), 0.92 (d, *J* = 9.8 Hz, 18H), 0.91 (d, *J* = 9.8 Hz, 18H), 0.81–0.71 (m, 6H). ^13^C{^1^H} NMR (126 MHz, CDCl_3_): δ 173.2, 137.7, 128.8, 128.62, 128.59, 128.55, 127.1, 126.7, 73.9, 73.5, 73.3, 73.1, 37.0, 36.3, 18.8, 18.72, 18.67, 18.6, 13.9, 11.4, 11.1. HRMS (ESI+) calcd for C_20_H_33_BrNaO_3_Si (M + Na), 451.1280; found, 451.1274.

##### 1-Bromo-3-(dimethylphenylsilyl)propan-2-yl (2*S*)-2-hydroxy-2-phenylacetate (**24**)

The product was obtained from allyldimethylphenylsilane (0.85 g, 4.8 mmol) following procedure D and stirring for 4 h. Purification by chromatography on silica (10:1 to 4:1 to 1:1 hexanes/ethyl acetate) gave **24** (0.409 g, 21%) as a colorless oil and partially separable mixture of diastereomers (*R*_f_ diastereomer α = 0.33, *R*_f_ diastereomer β = 0.32 in 4:1 hexanes/ethyl acetate).

IR (ATR): 3237, 3034, 2998, 2852, 1735, 1642, 1510, 1465, 1220, 1062, 864, 729 cm^–1^. ^1^H NMR (500 MHz, CDCl_3_): δ 7.65–7.61 (m, 2H), 7.56–7.51 (m, 4H), 7.48–7.29 (m, 14H), 5.19 (dtd, *J* = 8.6, 5.6, 4.5 Hz, 1H), 5.16–5.10 (m, 1H), 4.75 (d, *J* = 4.5 Hz, 2H), 3.48 (dd, *J* = 11.0, 3.9 Hz, 1H), 3.33 (dd, *J* = 11.0, 5.5 Hz, 1H), 3.24 (dd, *J* = 10.9, 4.5 Hz, 1H), 3.17 (dd, *J* = 10.9, 5.4 Hz, 1H), 1.41 (dd, *J* = 14.8, 8.6 Hz, 1H), 1.31 (dd, *J* = 14.8, 5.7 Hz, 1H), 1.26 (dd, *J* = 15.0, 7.5 Hz, 1H), 1.21 (dd, *J* = 14.9, 6.9 Hz, 1H), 0.40 (s, 3H), 0.37 (s, 3H), 0.17 (s, 3H), 0.13 (s, 3H). ^13^C{^1^H} NMR (126 MHz, CDCl_3_): δ 172.9, 172.8, 139.2, 137.88, 137.85, 137.7, 133.5, 133.4, 133.1, 133.0, 129.7, 129.51, 129.48, 128.7, 128.6, 128.5, 128.4, 128.11, 128.07, 127.9, 127.8, 126.9, 126.7, 73.5, 73.2, 73.0, 72.8, 36.3, 35.9, 20.9, 20.7, 0.7, 0.5, 0.0, −2.3, −2.7, −2.8, −3.0. HRMS (ESI+) calcd for C_19_H_23_BrNaO_3_Si (M + Na), 429.0498; found, 429.0498.

##### 3-(Dimethyl(phenyl)silyl)-2-fluoropropyl Pivalate (**4-piv**)

To a solution of **4** (0.202 g, 0.95 mmol) in dry DCM (4.8 mL) under N_2_ at 0 °C were added pyridine (0.23 mL, 2.9 mmol) and pivaloyl chloride (0.28 mL, 2.3 mmol). The mixture was allowed to slowly warm to room temperature while stirring for 16 h. The reaction was quenched with aq NaHCO_3_ (30 mL) and extracted with DCM (3 × 20 mL). The combined organic extracts were dried with MgSO_4_ and filtered before removing the solvent on a rotary evaporator. The crude product was purified by column chromatography on silica (4:1 hexanes/ethyl acetate, *R*_f_ ∼ 0.7) to yield the pivaloyl ester **4-piv** as a colorless liquid (0.24 g, 0.82 mmol, 85%).

IR (ATR): 3254, 3065, 2988, 2765, 1625, 1560, 1433, 1128, 1073, 935, 872, 695 cm^–1^. ^1^H NMR (500 MHz, CDCl_3_): δ 7.54–7.50 (m, 2H), 7.39–7.36 (m, 3H), 4.80 (ddddd, *J* = 49.1, 9.3, 6.9, 5.7, 2.5 Hz, 1H), 4.14 (ddd, *J* = 27.6, 12.4, 2.5 Hz, 1H), 4.03 (ddd, *J* = 20.7, 12.4, 6.8 Hz, 1H), 1.32 (ddd, *J* = 14.8, 12.4, 9.2 Hz, 1H), 1.21 (s, 9H), 1.14 (ddd, *J* = 36.5, 14.6, 5.7 Hz, 1H), 0.38 (d, *J* = 0.9 Hz, 3H), 0.36 (d, *J* = 0.9 Hz, 3H). ^13^C{^1^H} NMR (126 MHz, CDCl_3_): δ 178.2, 137.9, 133.5, 129.3, 128, 90.1 (d, *J* = 170.9 Hz), 67.4 (d, *J* = 22.9 Hz), 38.8, 27.2, 19.6 (d, *J* = 23.6 Hz), −2.2, −2.7. ^19^F NMR (470 MHz, CDCl_3_): δ −173.78 (ddddd, *J* = 48.7, 33.3, 27.7, 20.6, 12.4 Hz). HRMS (ESI+) calcd for C_16_H_25_FNaO_2_Si (M + Na), 319.1506; found, 315.1512.

##### 2-Fluoro-3-hydroxypropyl Pivalate (**25**)

To a solution of **4-piv** (81 mg, 0.273 mmol), KBr (49 mg, 0.41 mmol), and NaOAc (68 mg, 0.82 mmol) in acetic acid (1.3 mL) at 0 °C was added NaOAc (179 mg, 2.17 mmol) followed by peracetic acid (0.96 mL, 4.7 mmol) and the mixture stirred for 15 min before adding additional peracetic acid (2.2 mL, 9.8 mmol). The flask was removed from the ice bath and allowed to warm while stirring for 1 h. The reaction was carefully quenched by slowly adding aq Na_2_S_2_O_3_ (6 mL). Additional aq NaHCO_3_ was added until effervescence ceased (∼40 mL). The reaction mixture was extracted with DCM (3 × 20 mL), and the combined organic extracts were dried over MgSO_4_, filtered, and concentrated on a rotary evaporator. The crude product was purified by column chromatography on silica (4:1 hexanes/ethyl acetate, *R*_f_ ∼ 0.17) to yield alcohol **25** as a colorless liquid (15 mg, 0.086 mmol, 32%).

IR (ATR): 3315, 3021, 2987, 2856, 1610, 1543, 1415, 1311, 1274, 1118, 1073, 900, 867 cm^–1^. ^1^H NMR (500 MHz, CDCl_3_): δ 4.74 (dqd, *J* = 47.9, 4.9, 4.1 Hz, 1H), 4.37–4.25 (m, 2H), 3.79 (dd, *J* = 21.4, 4.8 Hz, 2H), 1.22 (s, 9H). ^13^C{^1^H} NMR (126 MHz, CDCl_3_): δ 178.5, 91.2 (d, *J* = 173.5 Hz), 62.5 (d, *J* = 24.0 Hz), 61.8 (d, *J* = 23.2 Hz), 38.9, 27.1. ^19^F NMR (470 MHz, CDCl_3_): δ −197.46 (dq, *J* = 48.3, 21.6 Hz). HRMS (ESI+) calcd for C_8_H_16_FO_3_ (M + H), 179.1083; found, 179.1075.

#### Conformational Analysis by DFT

Geometries of gas-phase (isolated) molecules were optimized within DFT using the ORCA 4.1 software package. Calculations used the PBE functional (a generalized gradient approximation) and def2-TZVP basis set. For select calculations, PBE results were compared to calculations using the B3LYP hybrid functional and def2-TZVP basis set, and we observed no significant differences in geometry or relative structural energy. To ensure that minimum-energy structures were identified, molecules were computed using a variety of initial geometries, with OCCF, SiCCF, and CCOH dihedral angles near their relative energy minima (60, 180, and 300°) and (in the case of the triphenyl molecule) the phenyl groups arranged in two orientations. In coordinate scans, the SiCCF dihedral angle was fixed in increments of 10° while other parameters were allowed to relax.

## Data Availability

The data underlying this study are available in the published article and its Supporting Information.

## Supporting Information Available

Copies of ^1^H NMR and ^13^C{^1^H} spectra, data from changing HF·Et_3_N equivalents for epoxide opening, procedures with data for Mosher ester analysis of enantiomerically enriched fluorohydrin products, and crystallographic data for compound **2** (PDF)
