# Supplementary material for: Regioselective Fluorohydrin Synthesis from Allylsilanes and Evidence for a Silicon–Fluorine Gauche Effect
Source: J Org Chem. 2024 Mar 8;89(7):4309–18. doi: 10.1021/acs.joc.3c02163 (PMC11002936; doi:10.1021/acs.joc.3c02163)
Supplement: Supplementary file 1 — jo3c02163_si_001.pdf [file jo3c02163_si_001.pdf]

Supporting Information for

Regioselective Fluorohydrin Synthesis from Allylsilanes and Evidence for a Silicon-Fluorine Gauche Effect

Alexie W. Clover,[a] Adam P. Jones,[a] Robert F. Berger,[a] Werner Kaminsky,[b] and Gregory W. O'Neil [a,]*
[a]Department of Chemistry, Western Washington University, Bellingham, WA 98229 (USA)
[b]Department of Chemistry, University of Washington, Seattle, WA 98195 (USA)
*Corresponding author. Email: oneilg@wwu.edu

Contents:

NMR Spectra for Compound:	Page
2-fluoro-3-(trimethylsilyl)propan-1-ol (**1**)	S2
2-fluoro-3-(triphenylsilyl)propan-1-ol (**2**)	S3
2-fluoro-3-(triisopropylsilyl)propan-1-ol (**3**)	S5
3-(dimethyl(phenyl)silyl)-2-fluoropropan-1-ol (**4**)	S6
3-(dimethyl(phenyl)silyl)-2-fluoropropyl pivalate (**4-piv**)	S8
3-(allyldiphenylsilyl)-2-fluoropropan-1-ol (**5**)	S9
3-(allyldimethylsilyl)-2-fluoropropan-1-ol (**6**)	S11
3-((bromomethyl)dimethylsilyl)-2-fluoropropan-1-ol (**7**)	S12
4-(dimethyl(phenyl)silyl)-3-fluorobutane-1,2-diol (**13**)	S14
4-(triphenyl)silyl)-3-fluorobutane-1,2-diol (**14**)	S15
triphenyl(oxiran-2-ylmethyl)silane (**18**)	S17
triisopropyl(oxiran-2-ylmethyl)silane (**19**)	S18
dimethylphenyl(oxiran-2-ylmethyl)silane (**20**)	S19
1-bromo-3-(triphenylsilyl)propan-2-yl (2*S*)-2-hydroxy-2-phenylacetate (**22**)	S20
1-bromo-3-(triisopropylsilyl)propan-2-yl (2*S*)-2-hydroxy-2-phenylacetate (**23**)	S21
1-bromo-3-(dimethylphenylsilyl)propan-2-yl (2*S*)-2-hydroxy-2-phenylacetate (**24**)	S22
2-fluoro-3-hydroxypropyl pivalate (**25**)	S23
Comparison of HF•Et_3N equiv. on reactions of epoxide **20**	S25
Mosher Ester Analysis of Enantioenriched Silylfluorohydrins **18**-**20**	S26
Crystallographic data for the structures of compound **2**.	S28

^{1}H NMR spectrum ($CDCl_3$, 500 MHz)

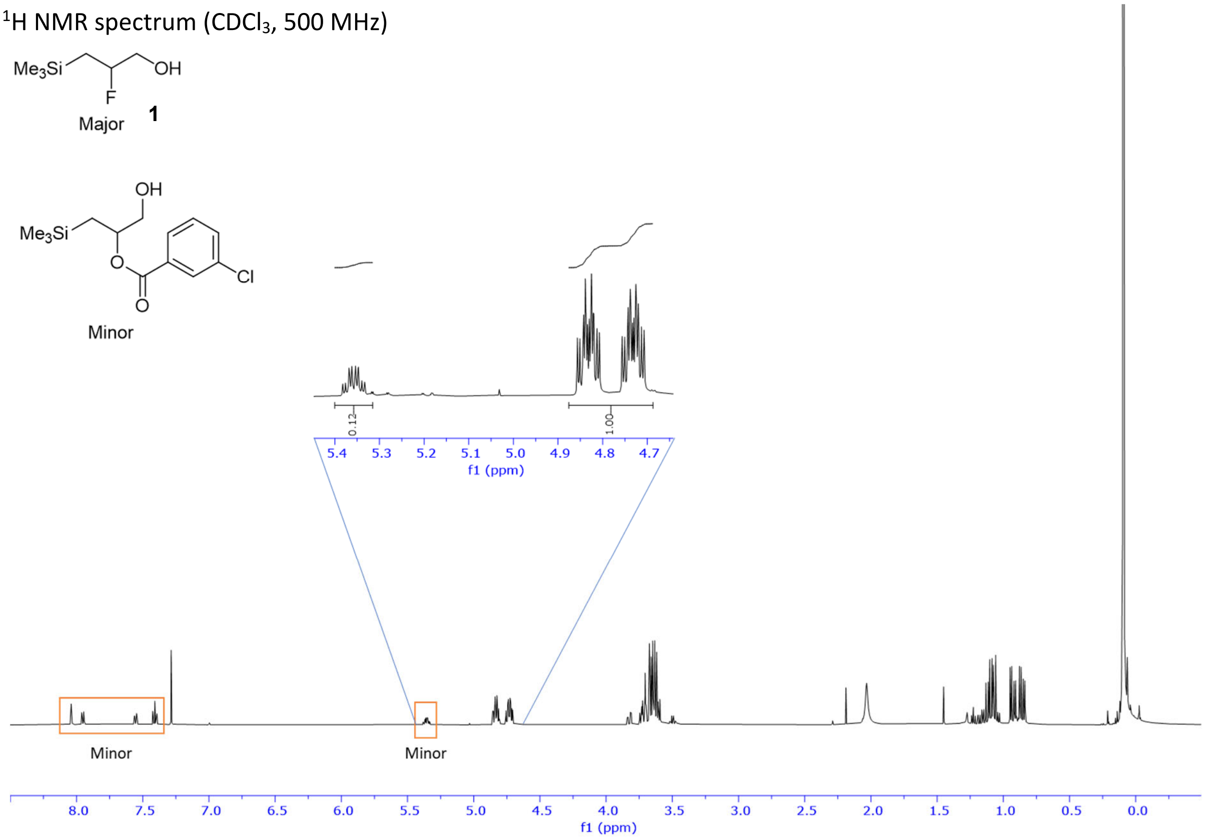

^{13}C{^{1}H} NMR spectrum ($CDCl_3$, 126 MHz)

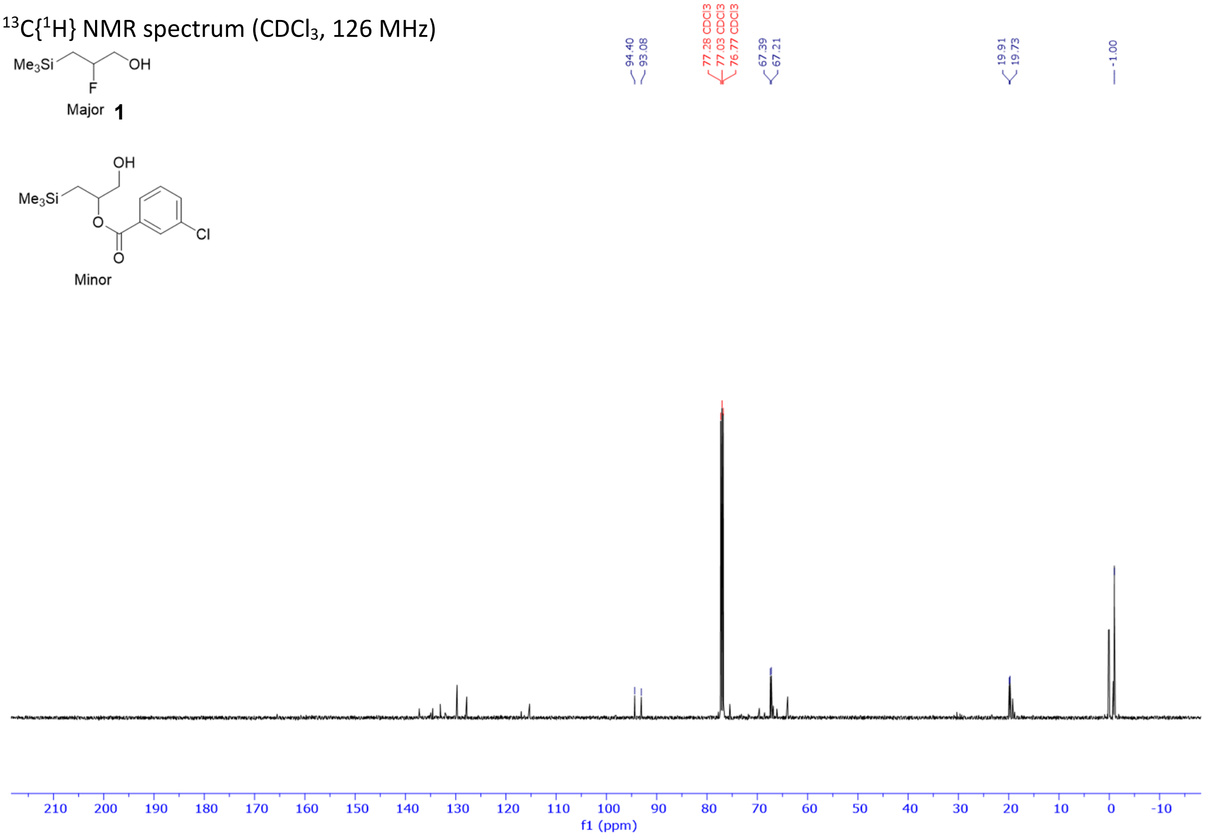

^{19}F NMR spectrum ($CDCl_3$, 470 MHz)

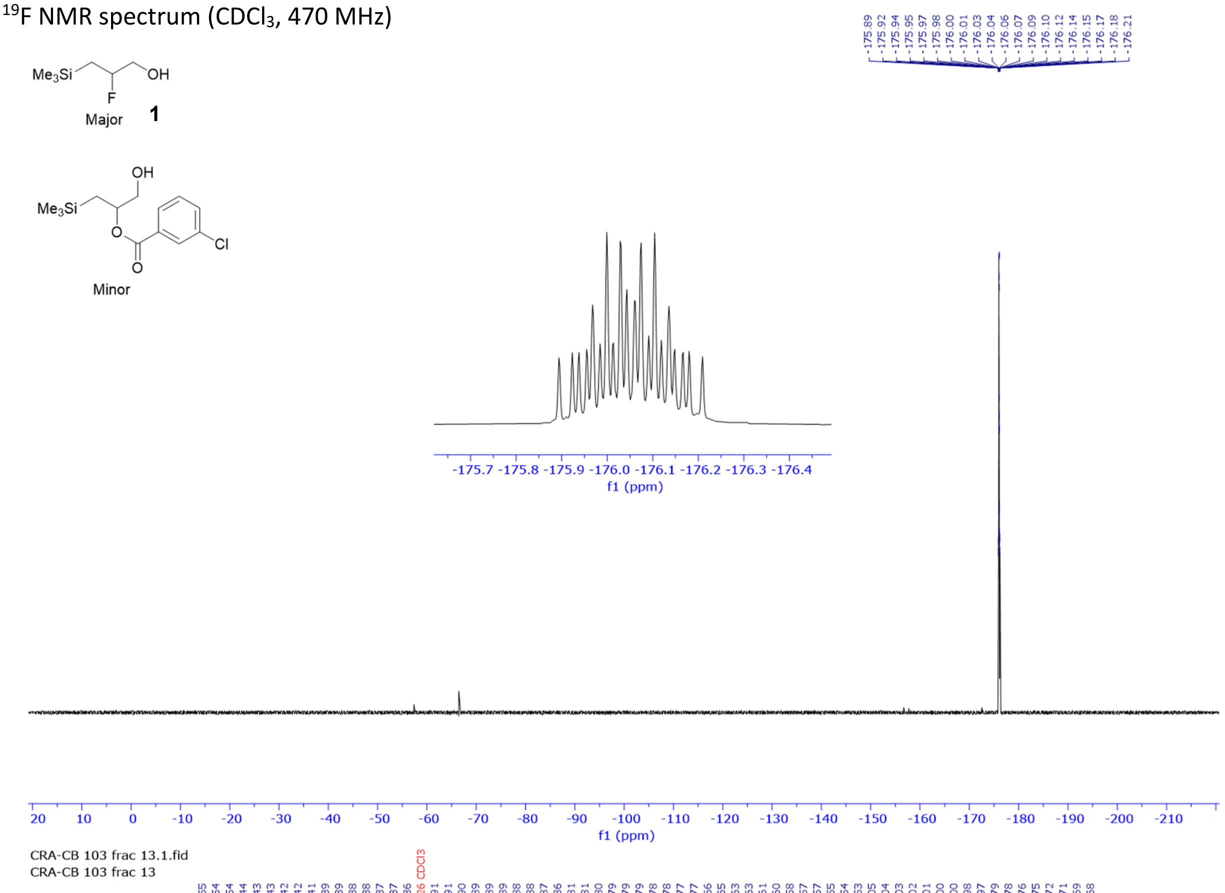

^{1}H NMR spectrum ($CDCl_3$, 500 MHz)

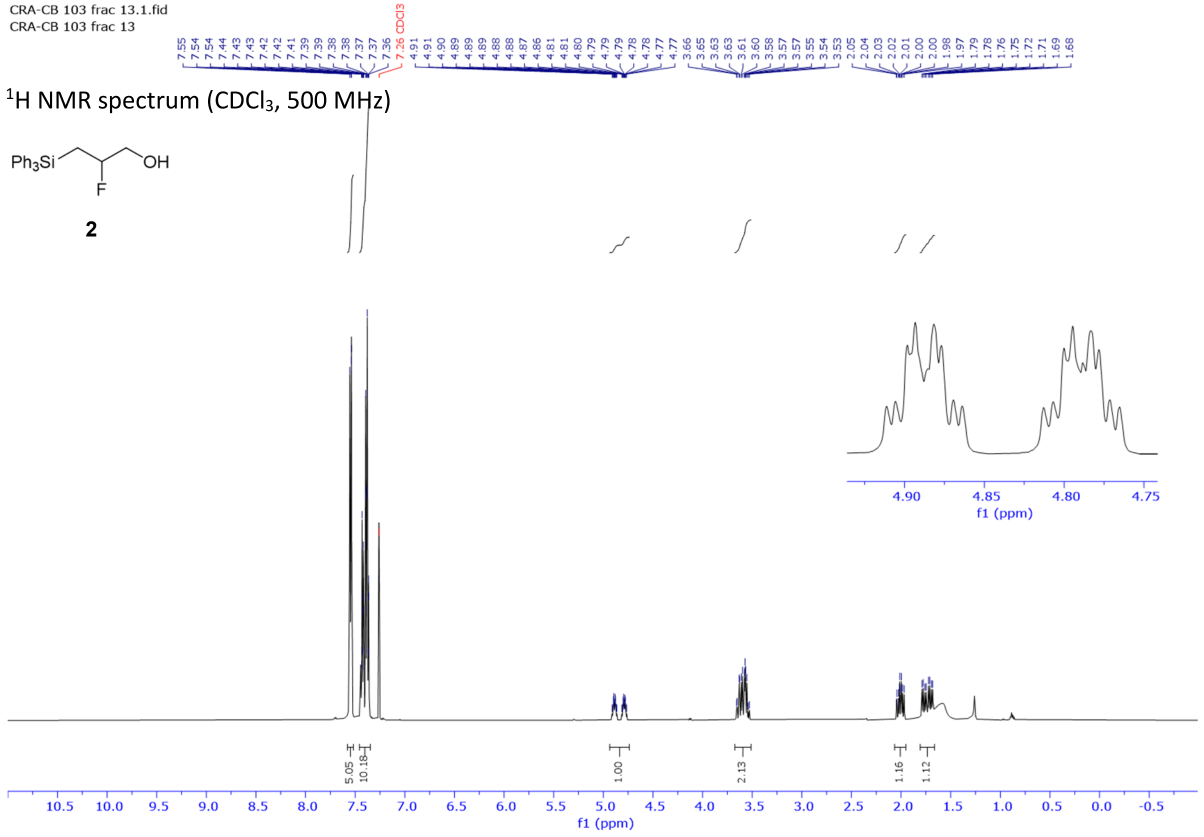

$^{13}C\{^{1}H\}$ NMR spectrum ($CDCl_3$, 126 MHz)

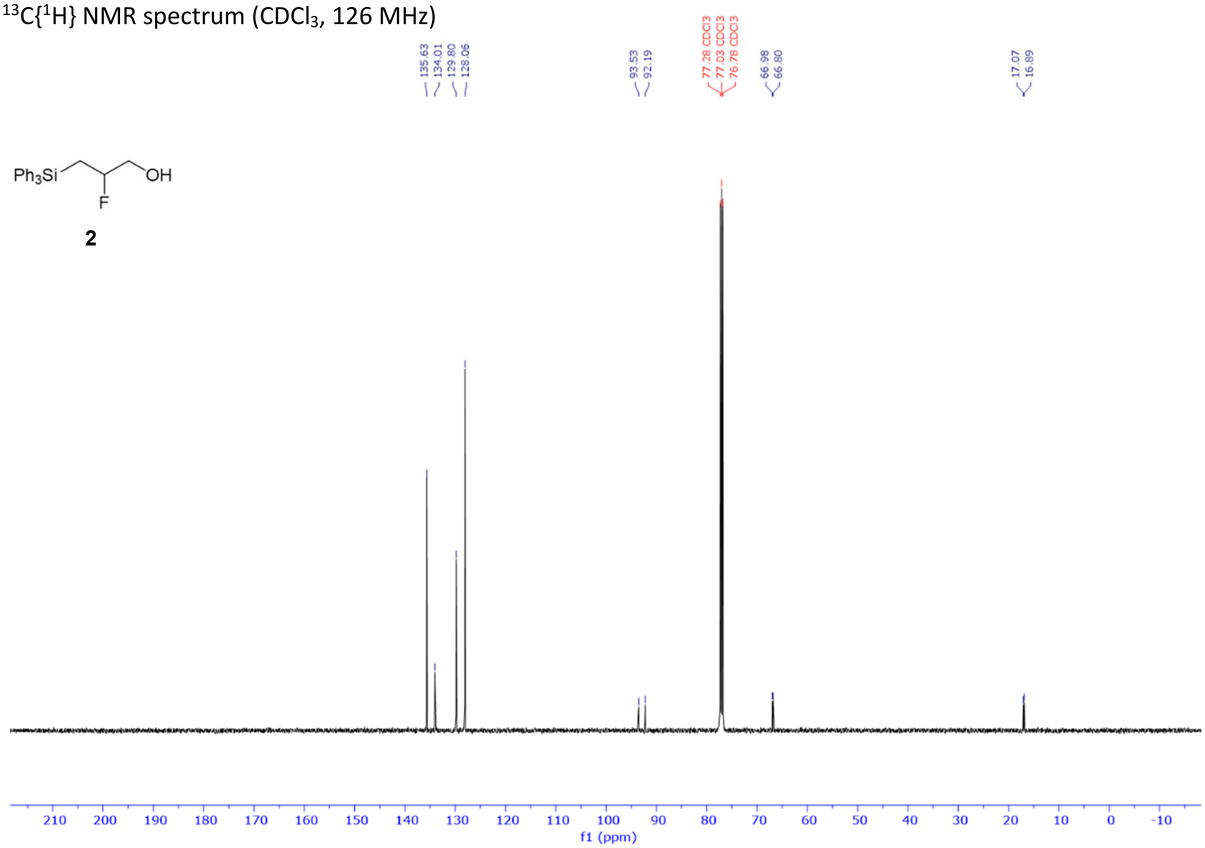

^{19}F NMR spectrum ($CDCl_3$, 470 MHz)

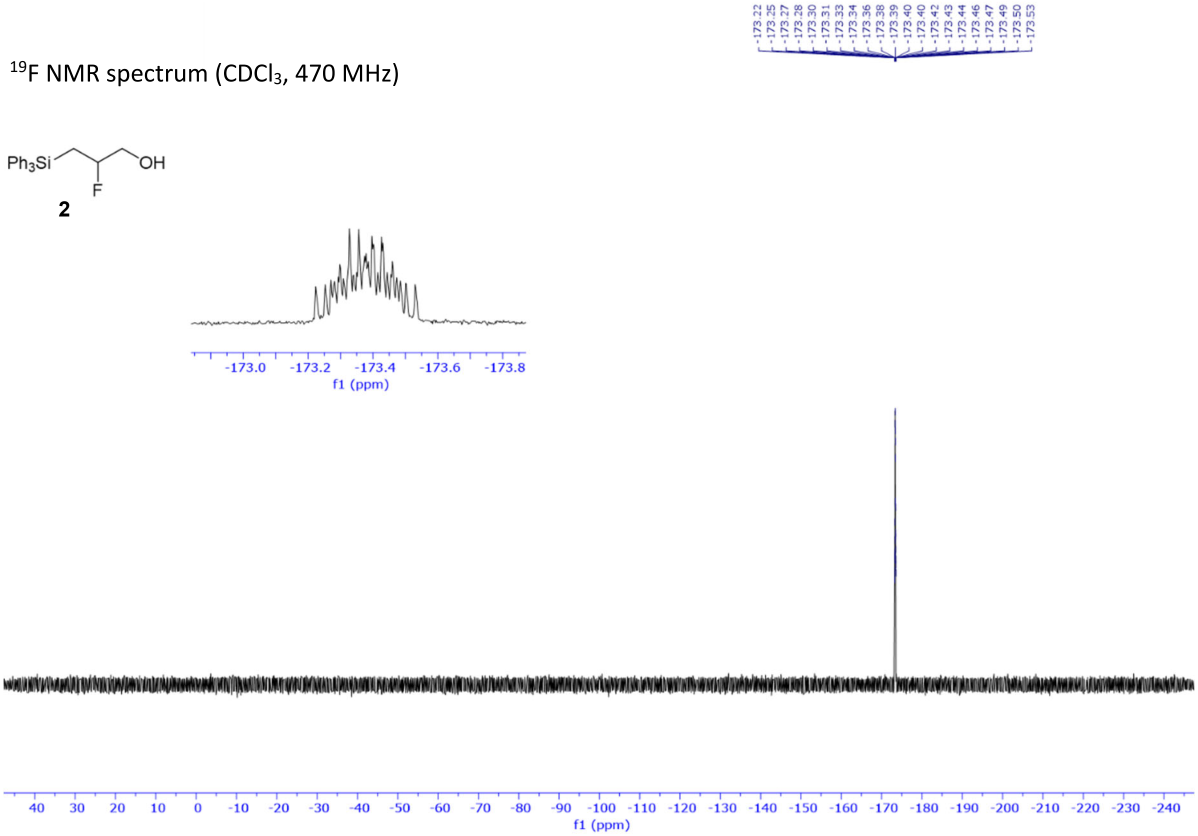

^{1}H NMR spectrum ($CDCl_3$, 500 MHz)

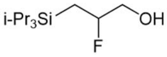

3

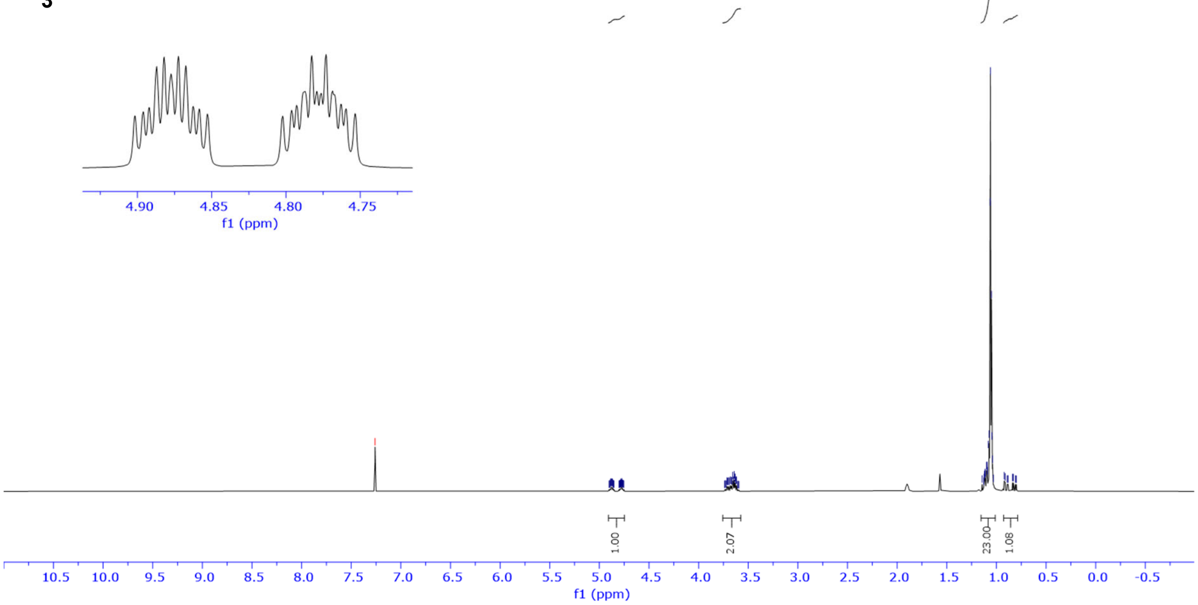

$^{13}C\{^{1}H\}$ NMR spectrum ($CDCl_3$, 126 MHz)

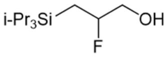

3

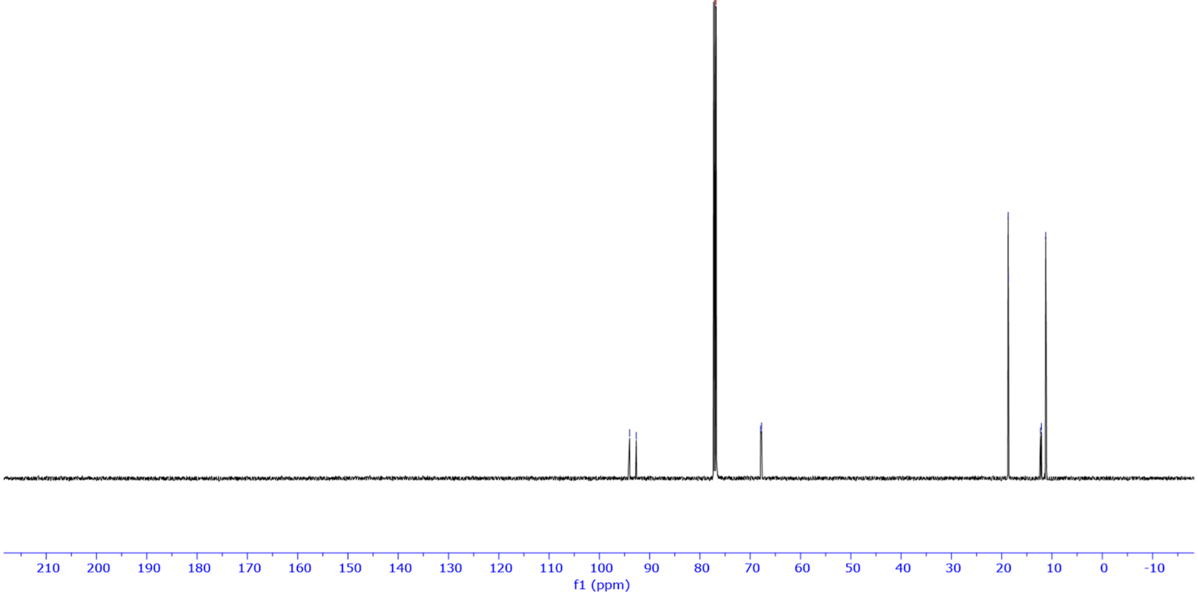

^{19}F NMR spectrum ($CDCl_3$, 470 MHz)

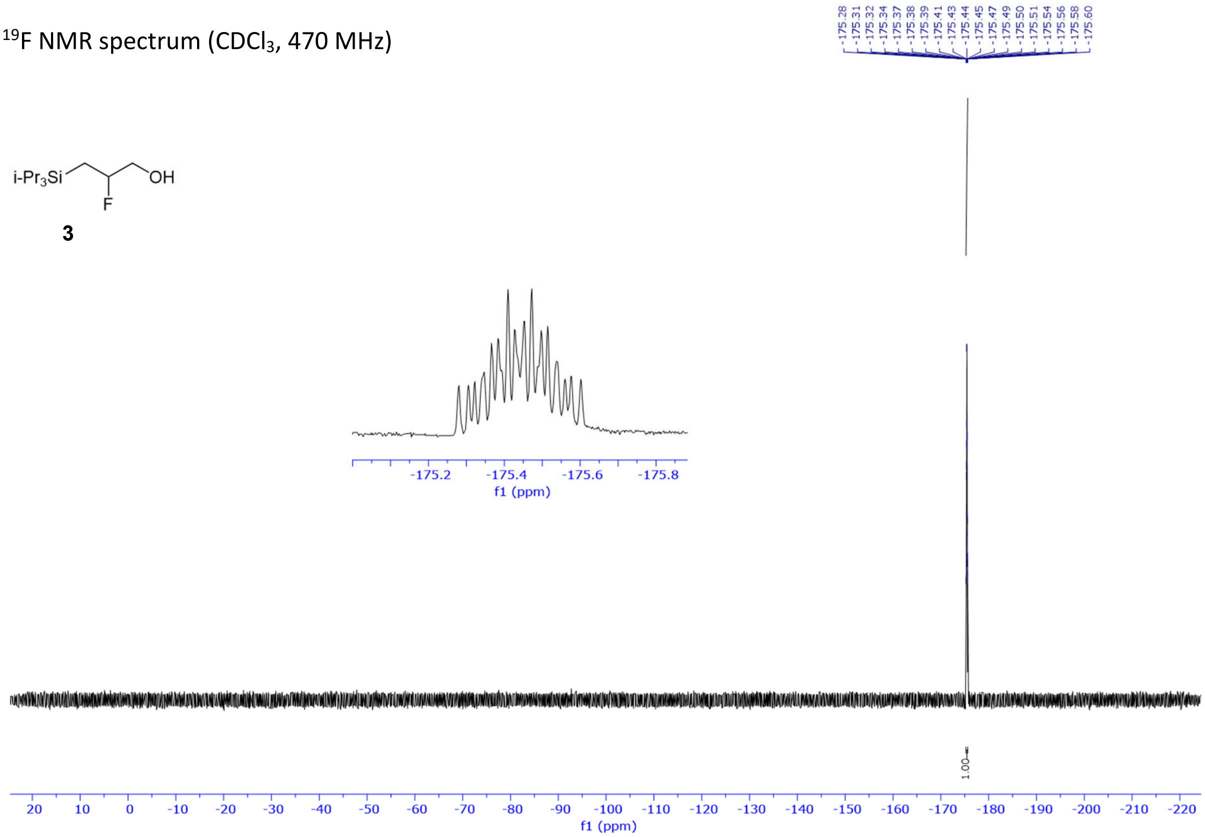

^{1}H NMR spectrum ($CDCl_3$, 500 MHz)

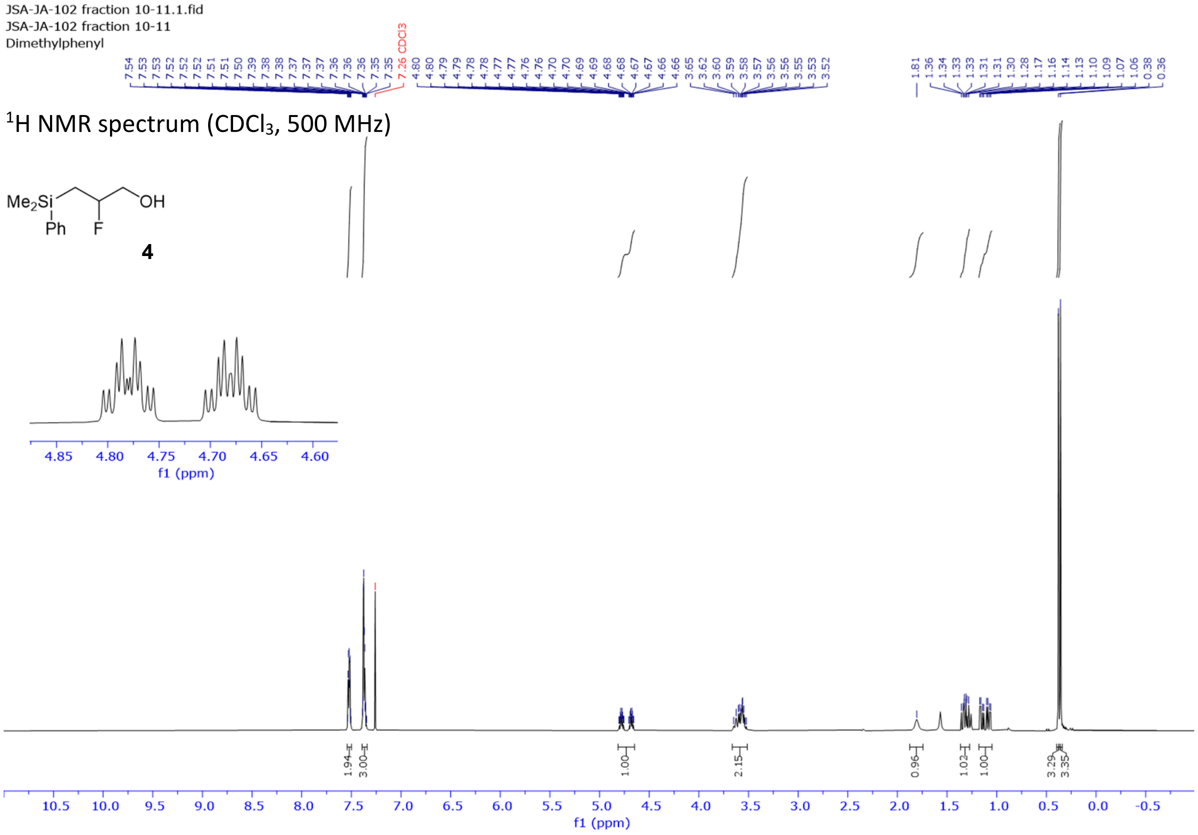

$^{13}C\{^{1}H\}$ NMR spectrum ($CDCl_3$, 126 MHz)

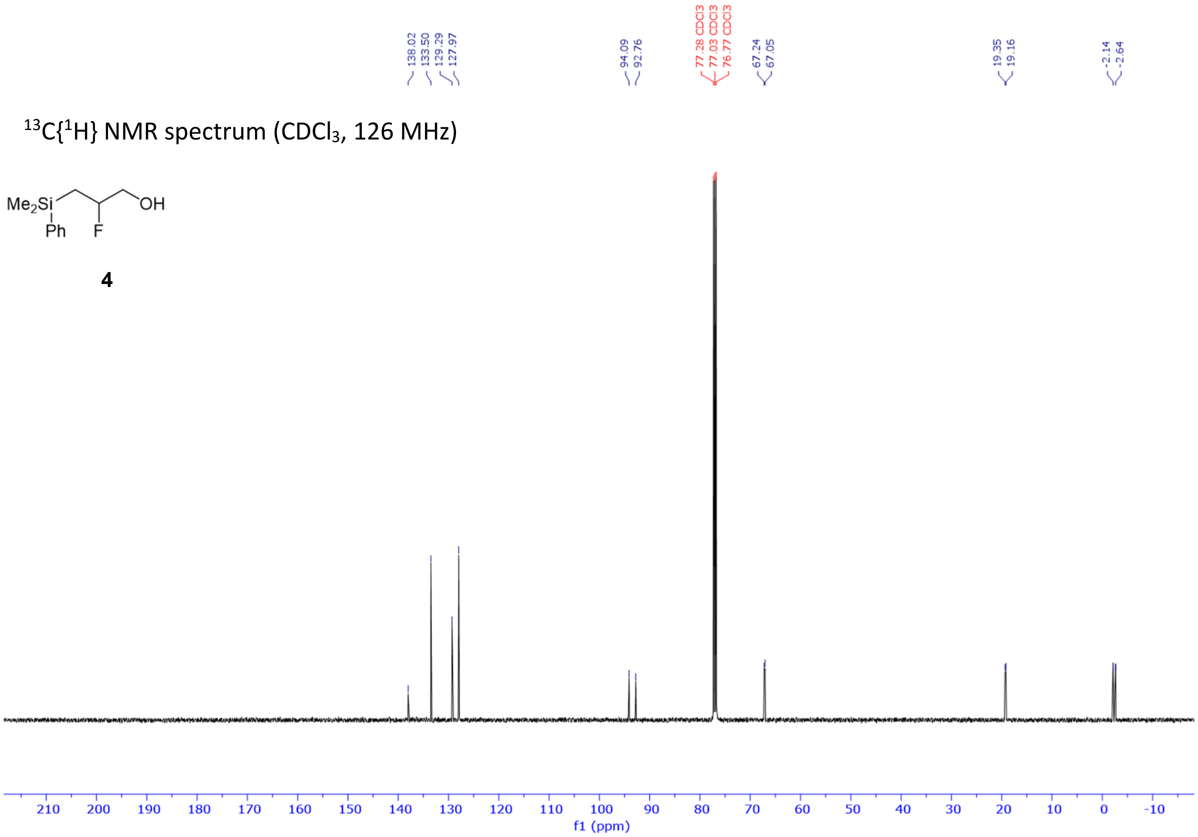

4

^{19}F NMR spectrum ($CDCl_3$, 470 MHz)

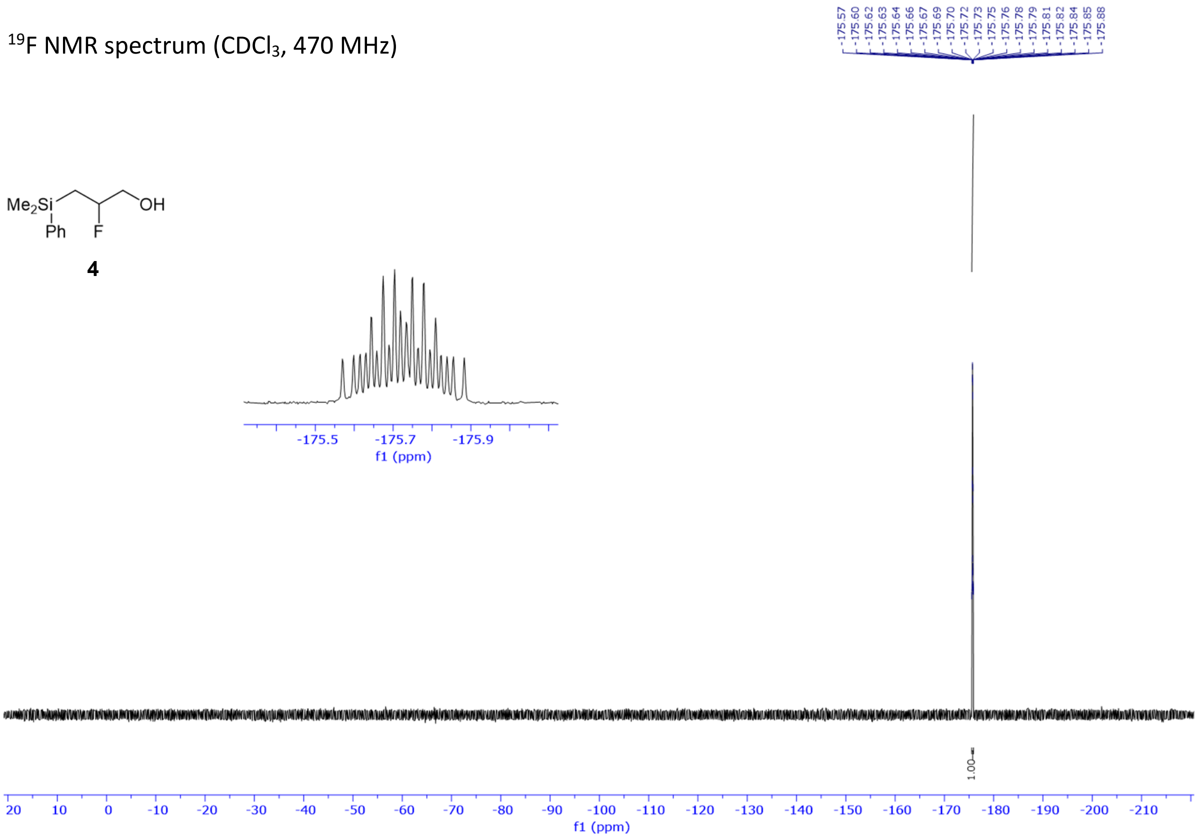

4

^{1}H NMR spectrum ($CDCl_3$, 500 MHz)

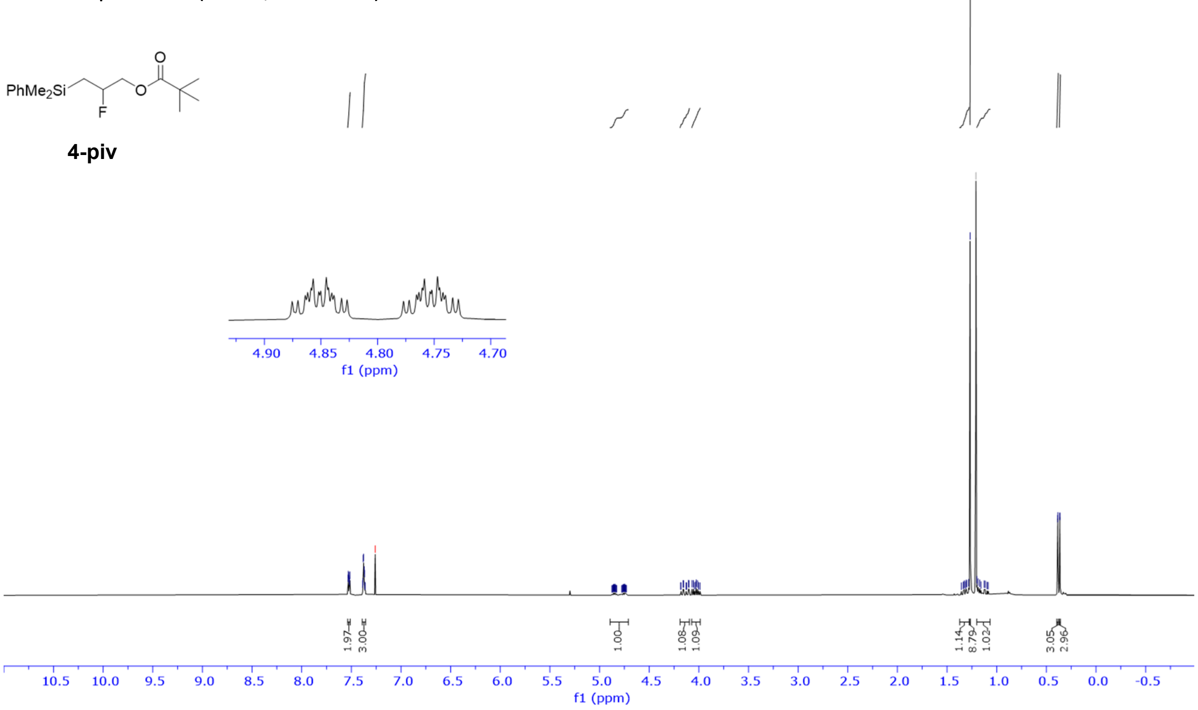

$^{13}C\{^{1}H\}$ NMR spectrum ($CDCl_3$, 126 MHz)

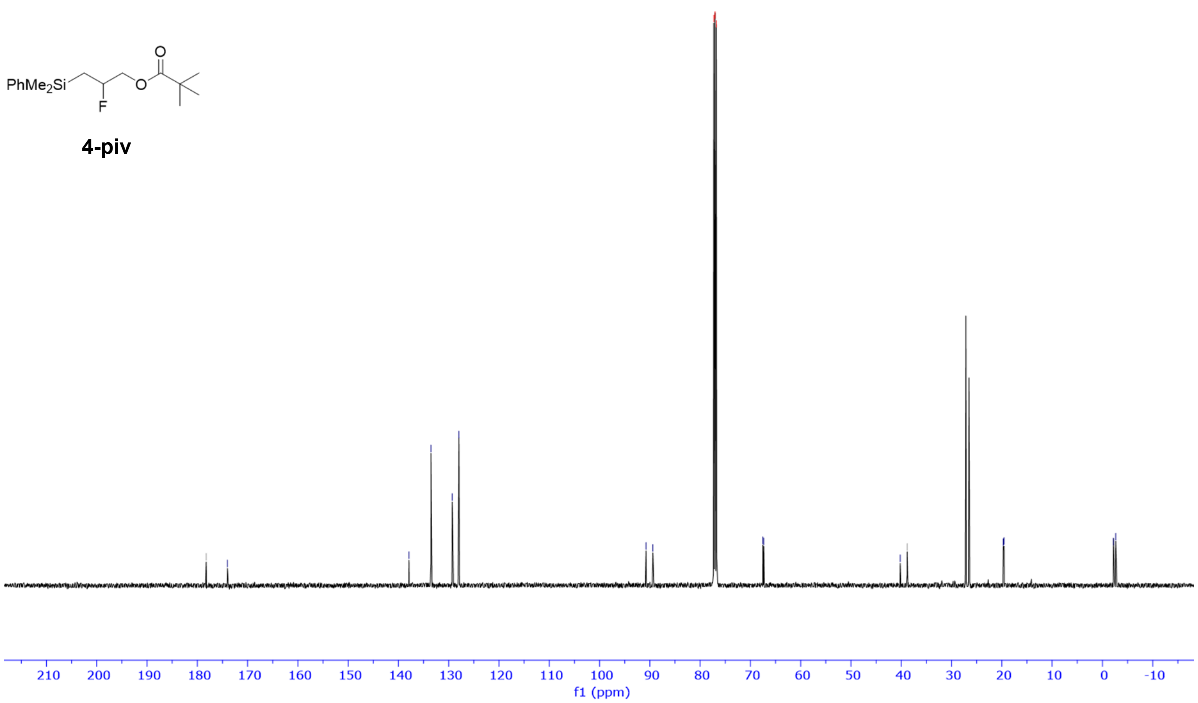

^{19}F NMR spectrum ($CDCl_3$, 470 MHz)

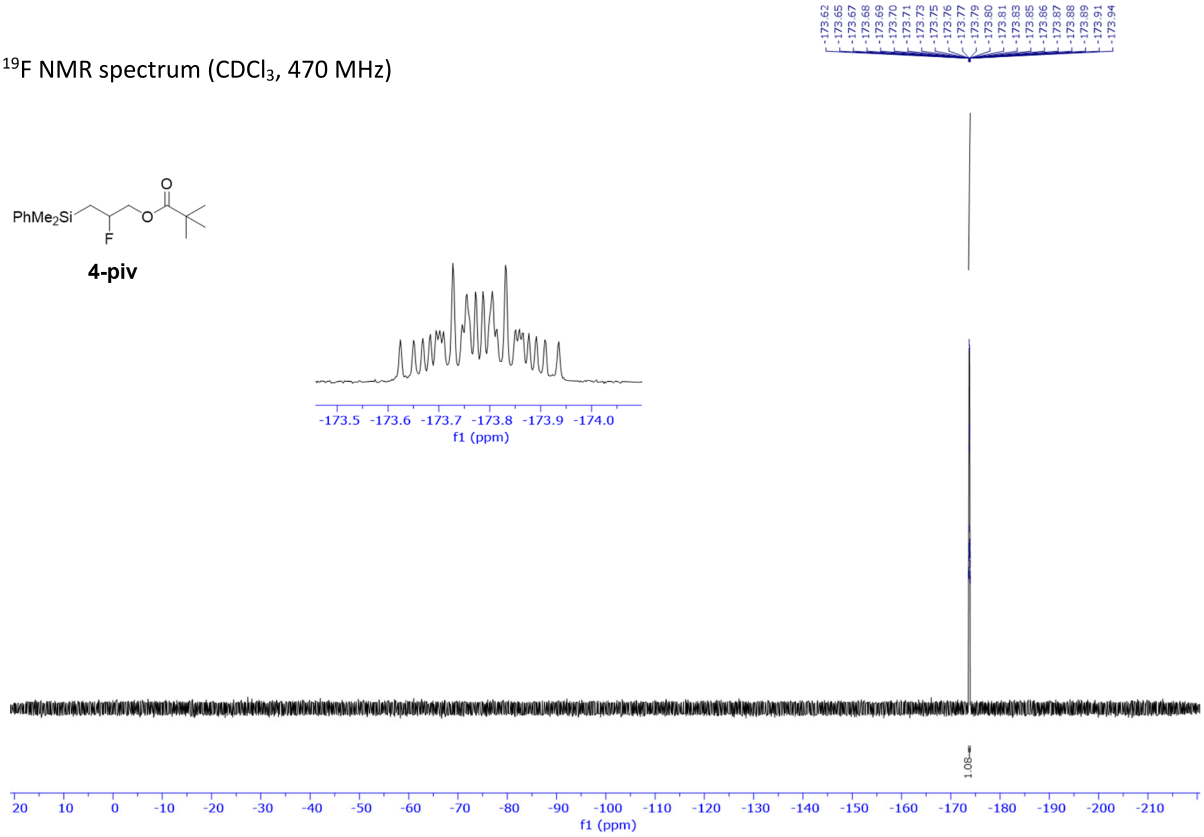

4-piv

^{1}H NMR spectrum ($CDCl_3$, 500 MHz)

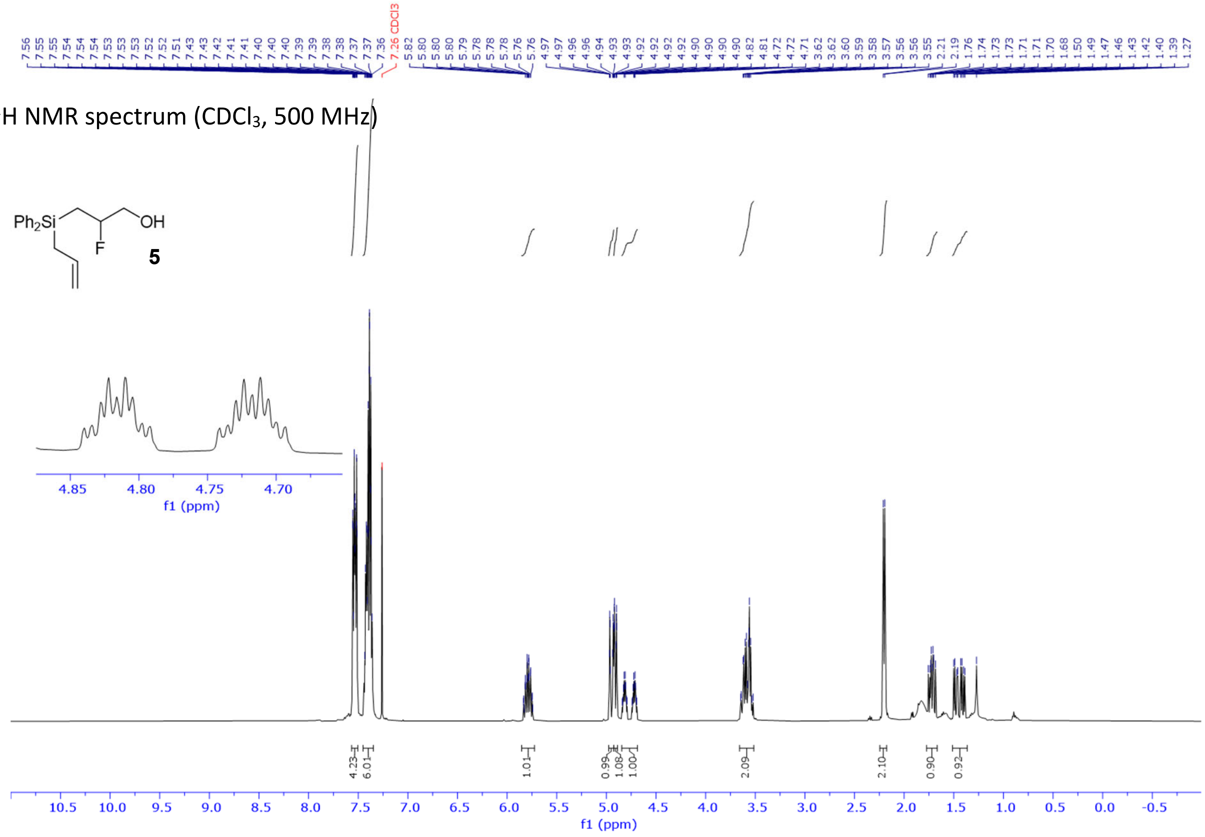

5

$^{13}C\{^1H\}$ NMR spectrum ($CDCl_3$, 126 MHz)

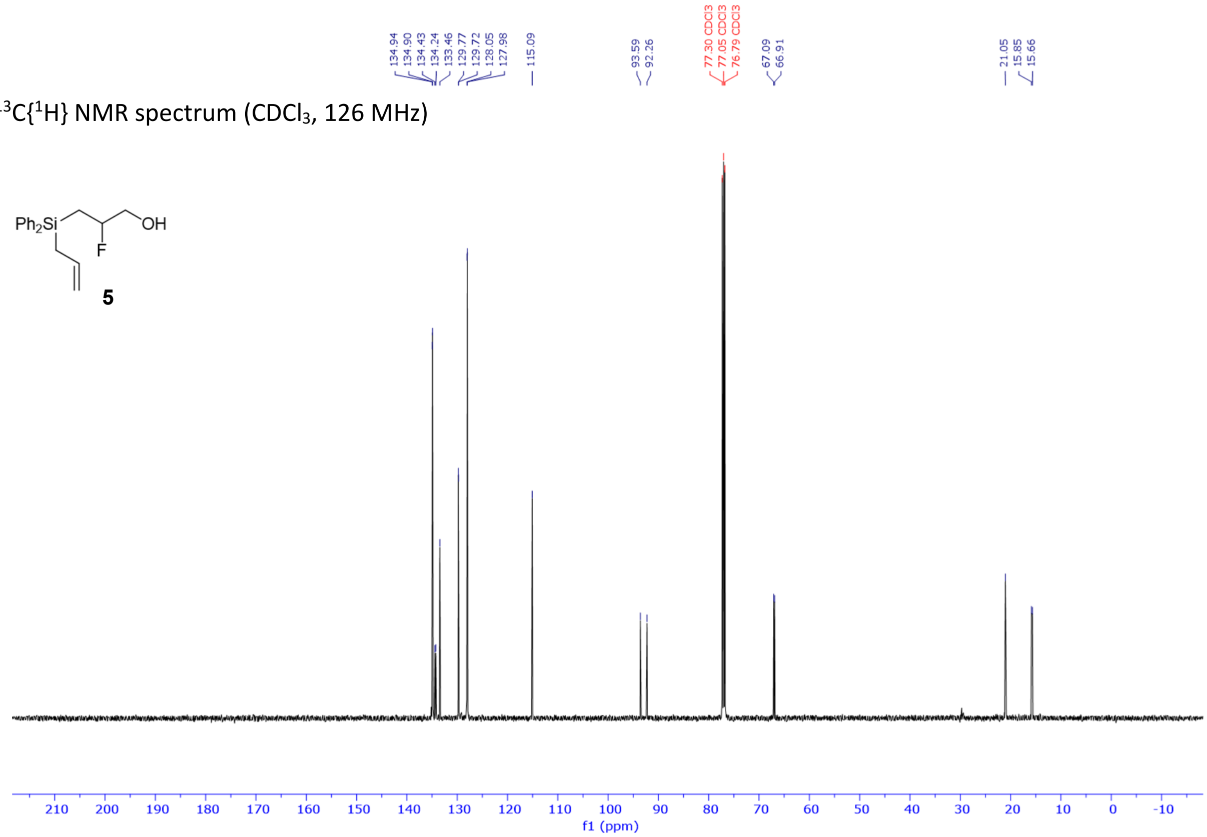

5

^{19}F NMR spectrum ($CDCl_3$, 470 MHz)

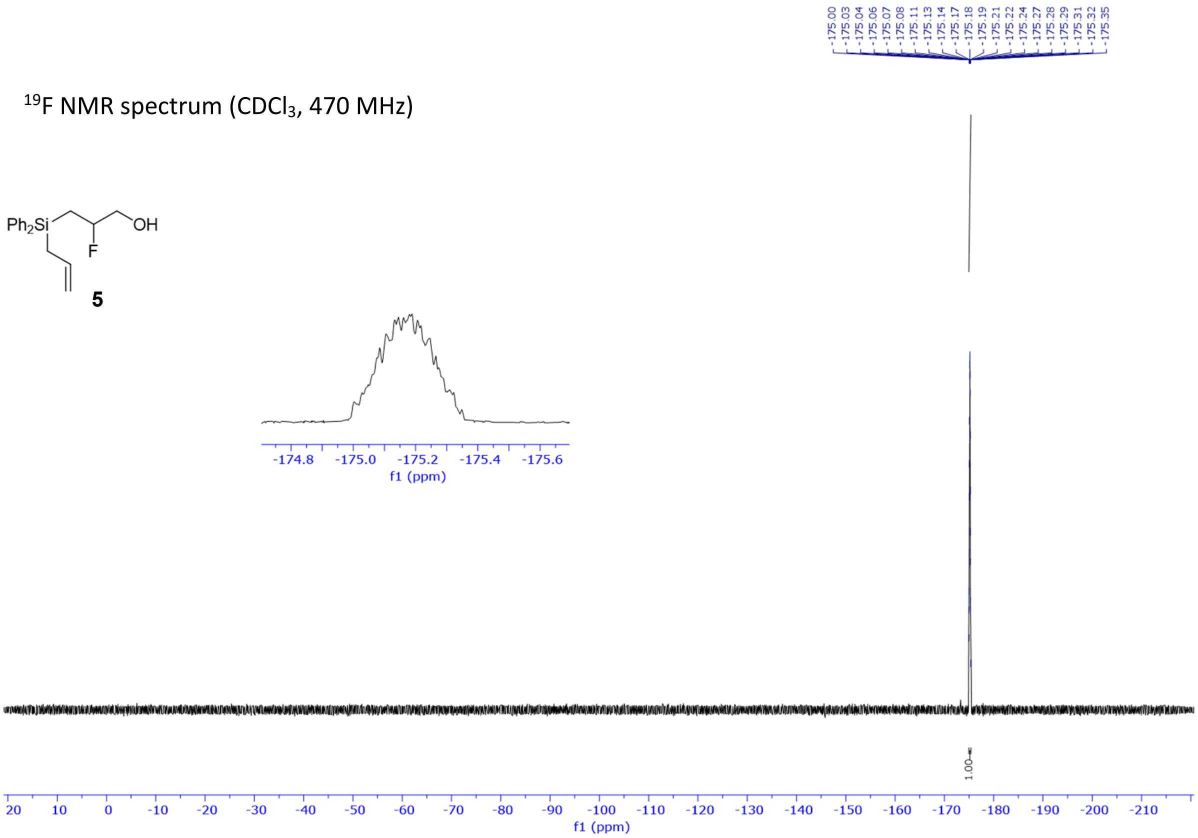

5

^{1}H NMR spectrum ($CDCl_3$, 500 MHz)

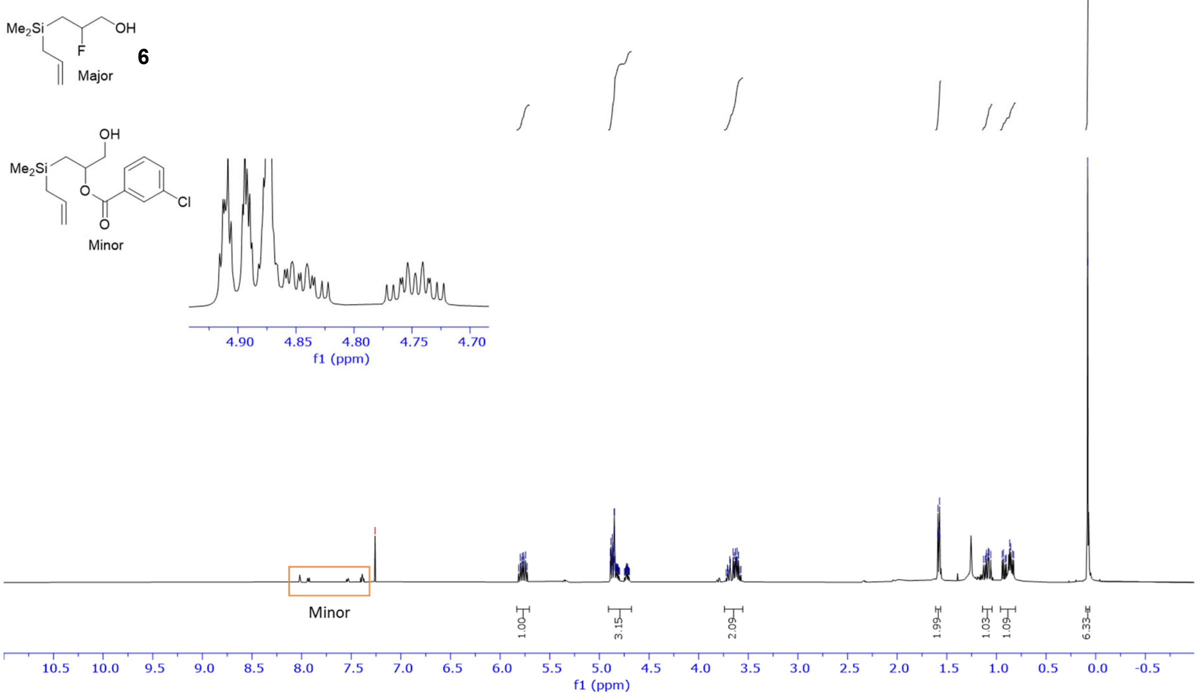

$^{13}C\{^{1}H\}$ NMR spectrum ($CDCl_3$, 126 MHz)

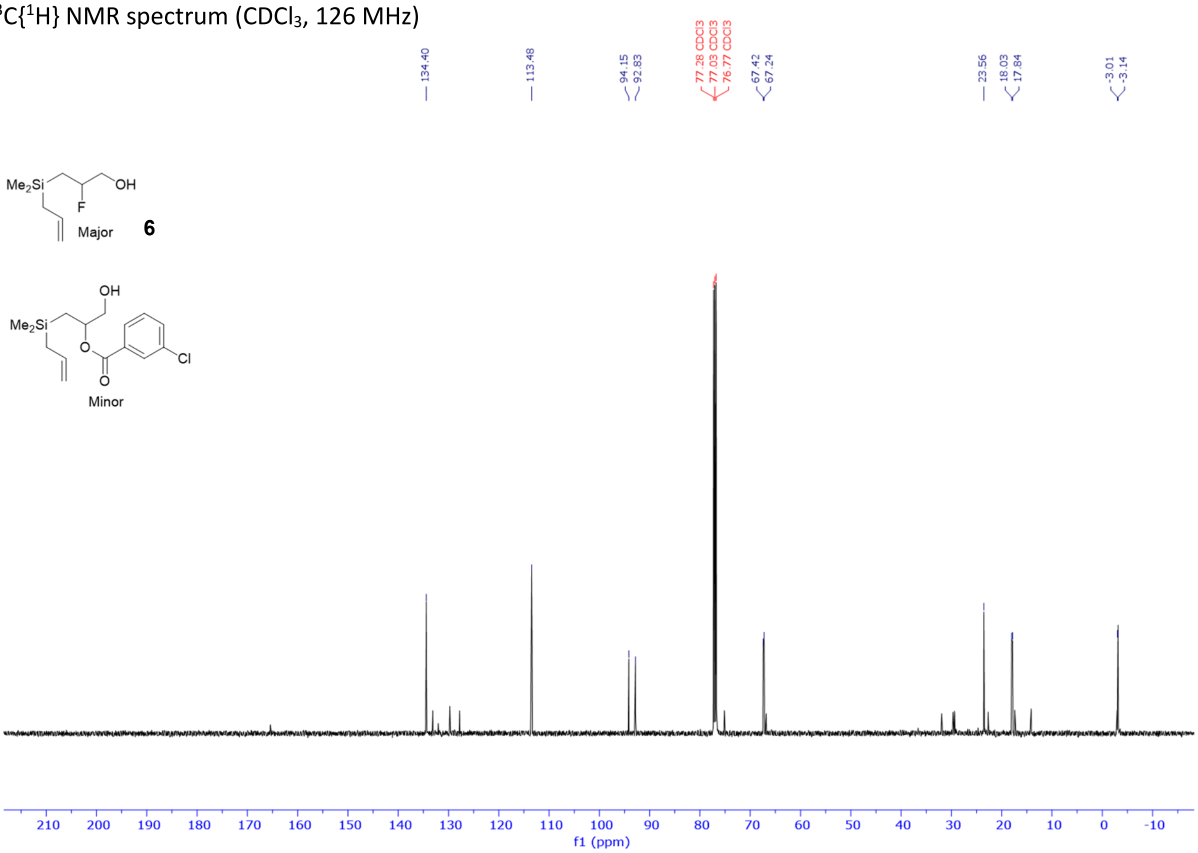

^{19}F NMR spectrum ($CDCl_3$, 470 MHz)

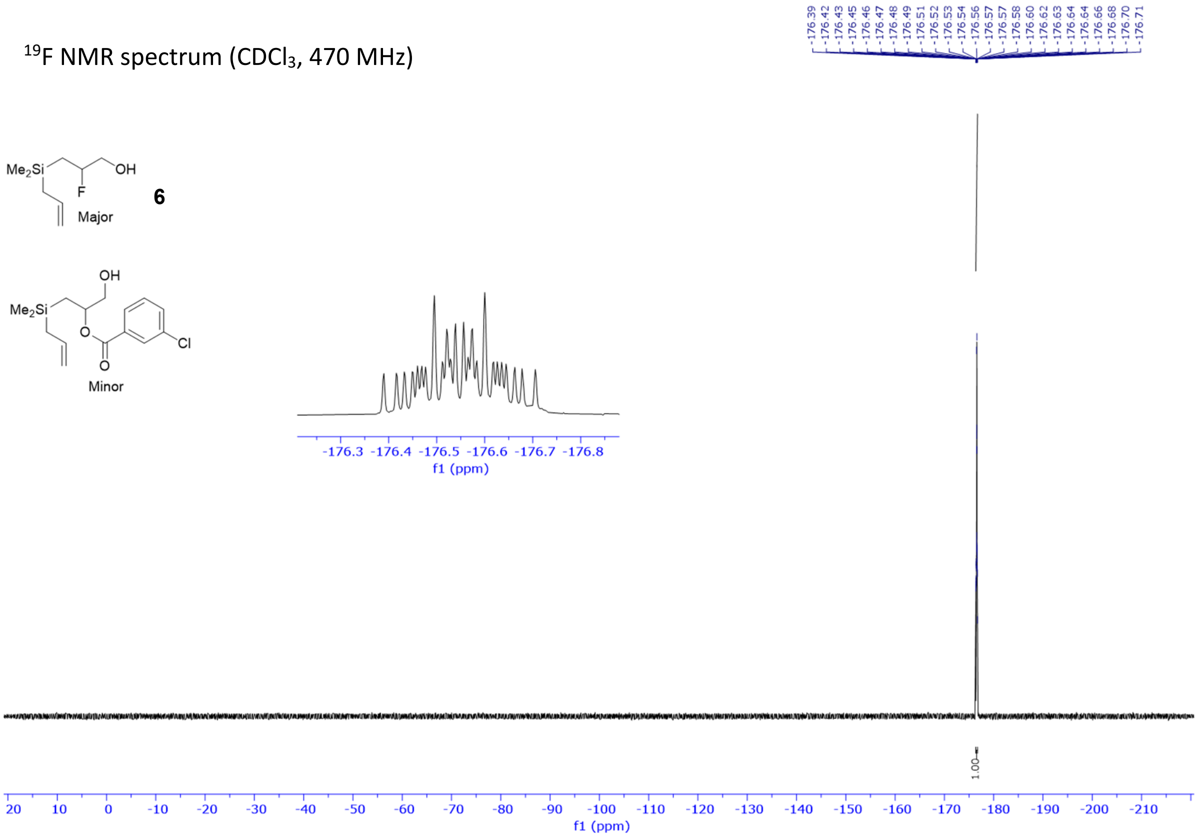

^{1}H NMR spectrum ($CDCl_3$, 500 MHz)

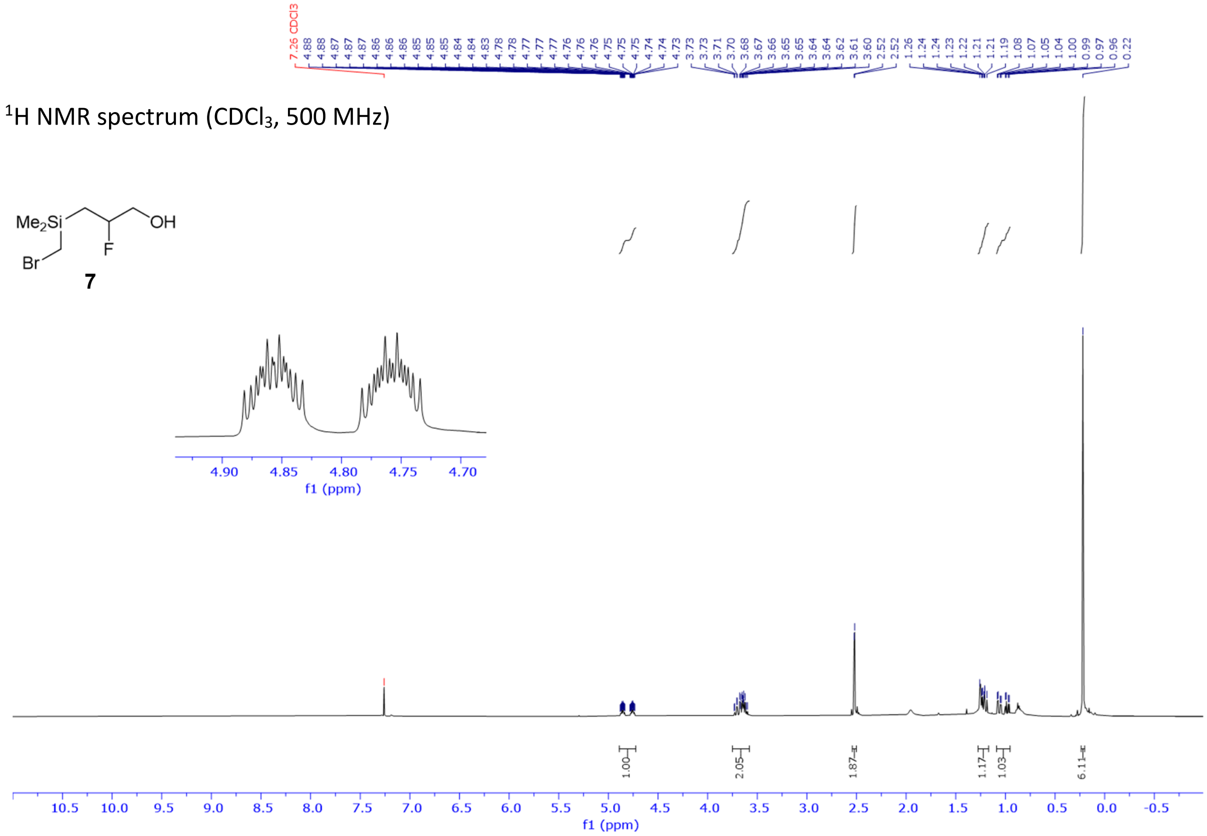

$^{13}C\{^1H\}$ NMR spectrum ($CDCl_3$, 126 MHz)

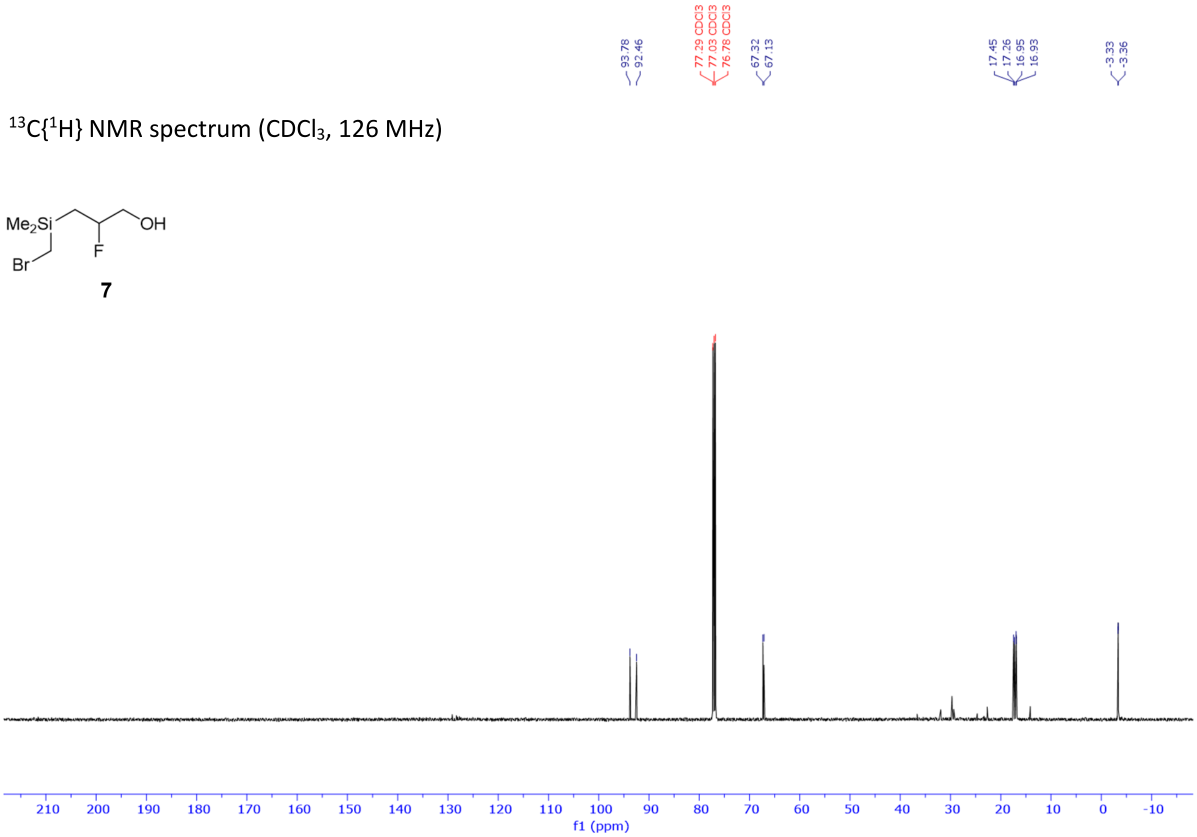

^{19}F NMR spectrum ($CDCl_3$, 470 MHz)

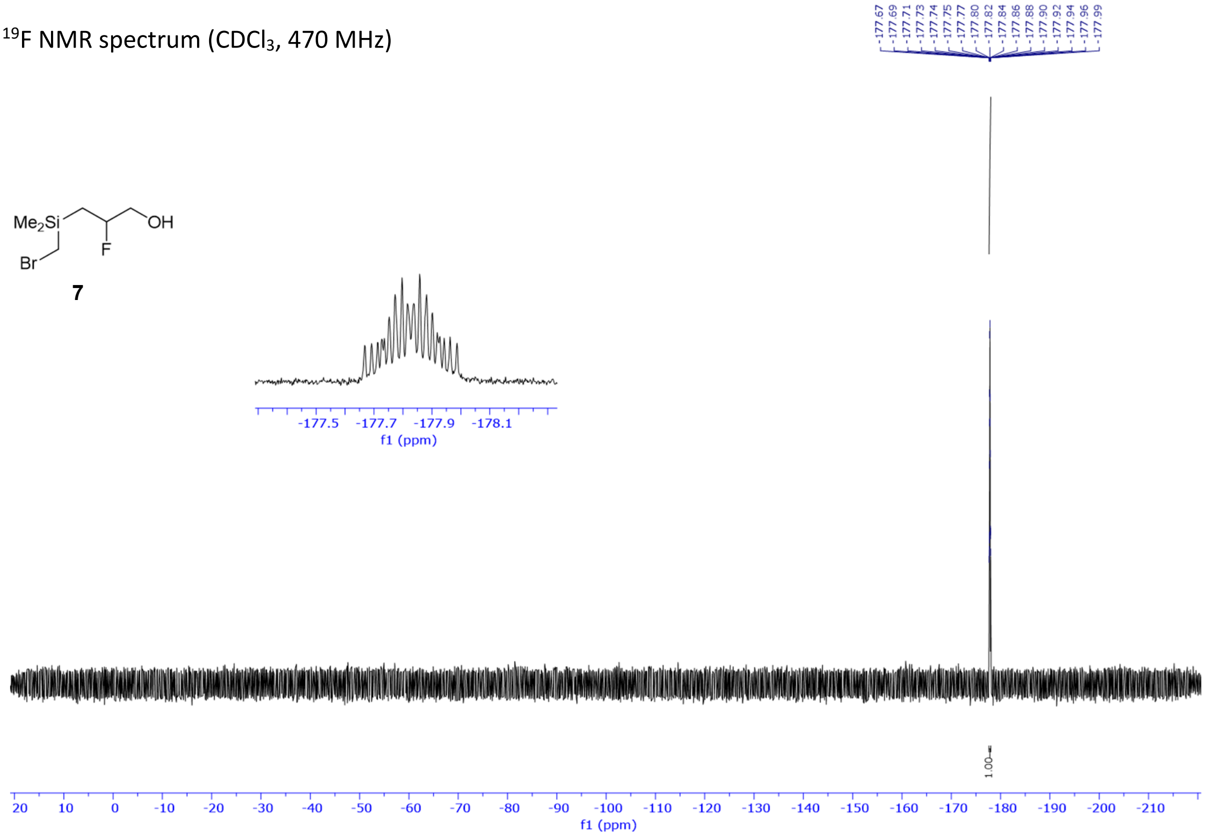

^{1}H NMR spectrum ($CDCl_3$, 500 MHz)

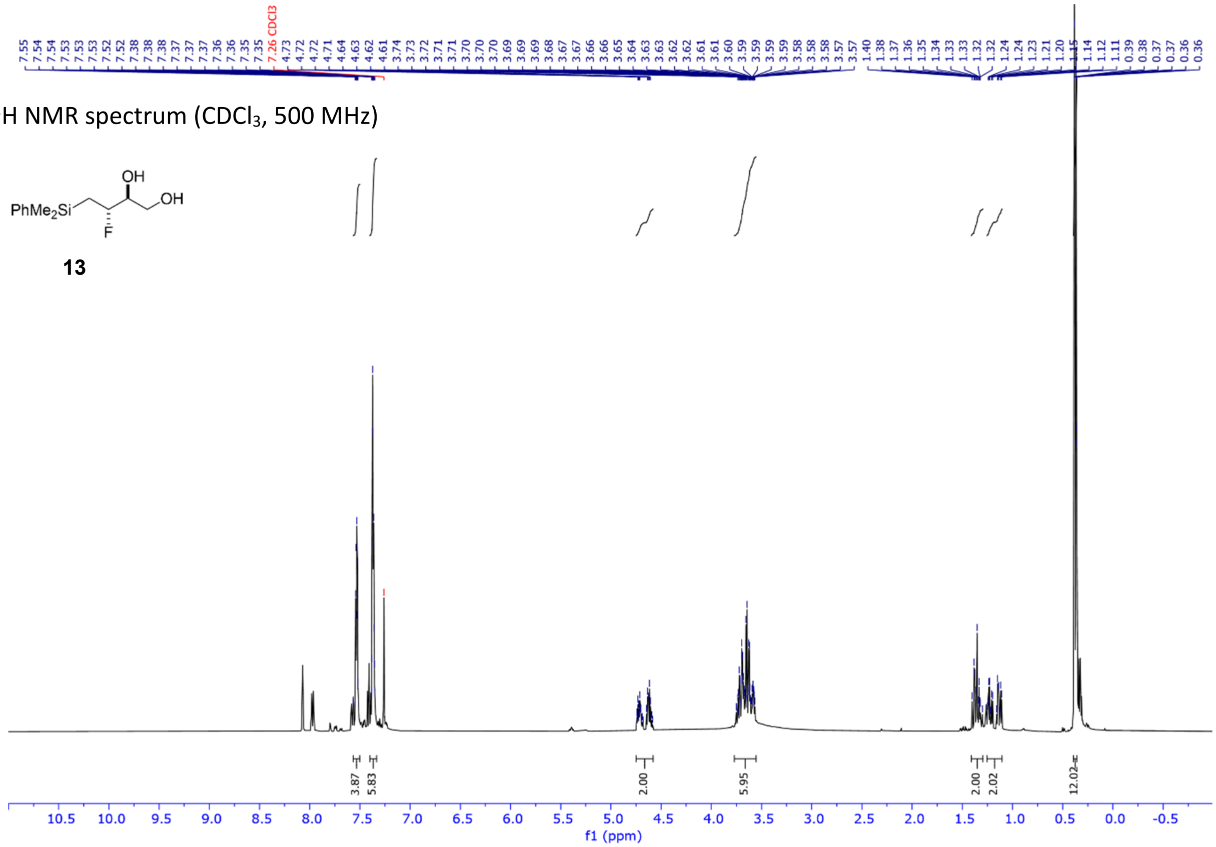

$^{13}C\{^{1}H\}$ NMR spectrum ($CDCl_3$, 126 MHz)

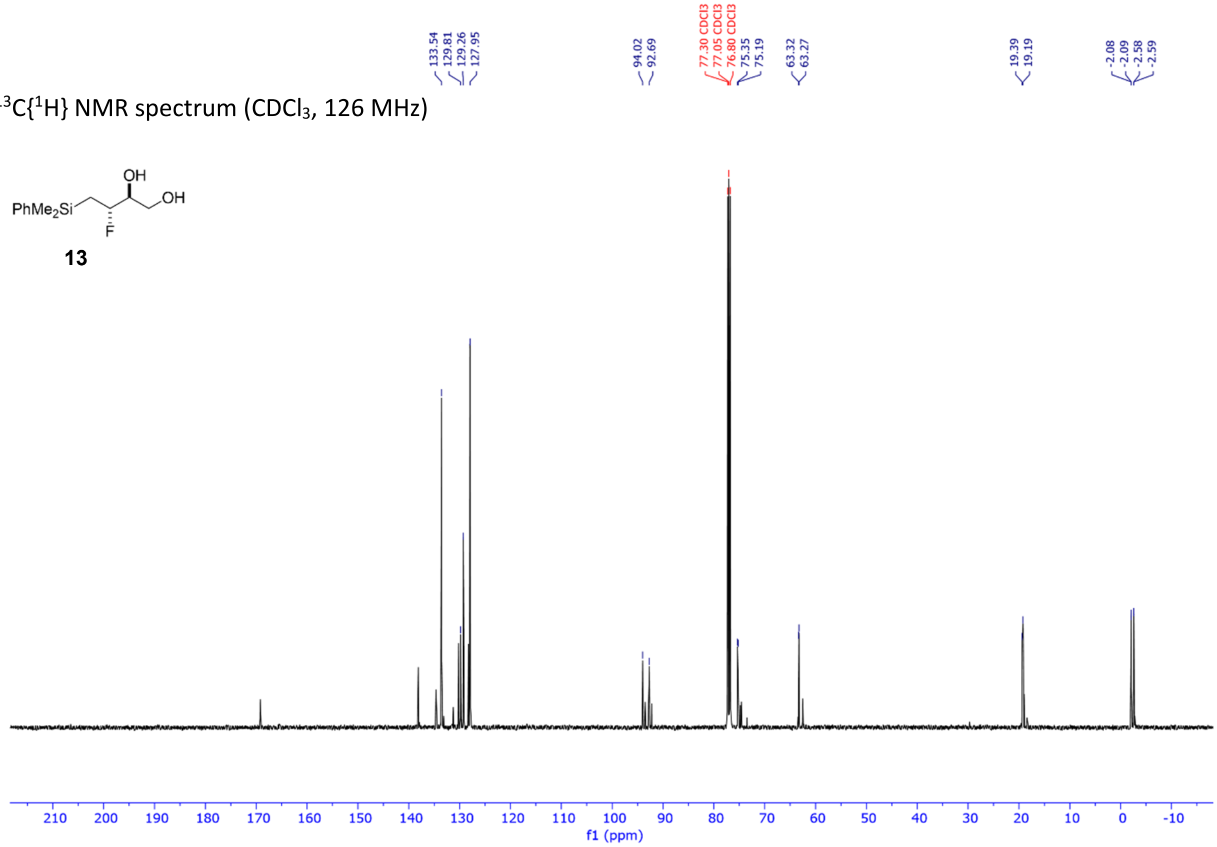

^{19}F NMR spectrum ($CDCl_3$, 470 MHz)

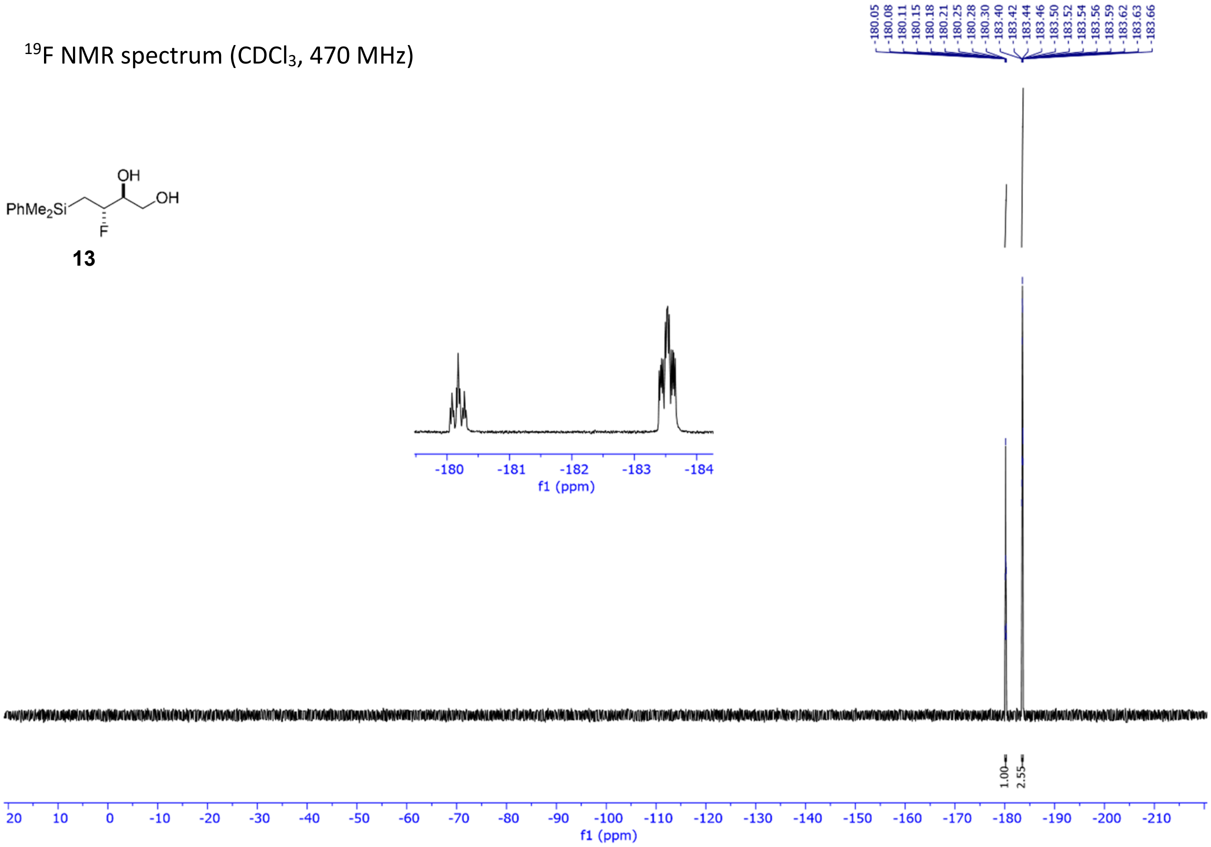

13

^{1}H NMR spectrum ($CDCl_3$, 500 MHz)

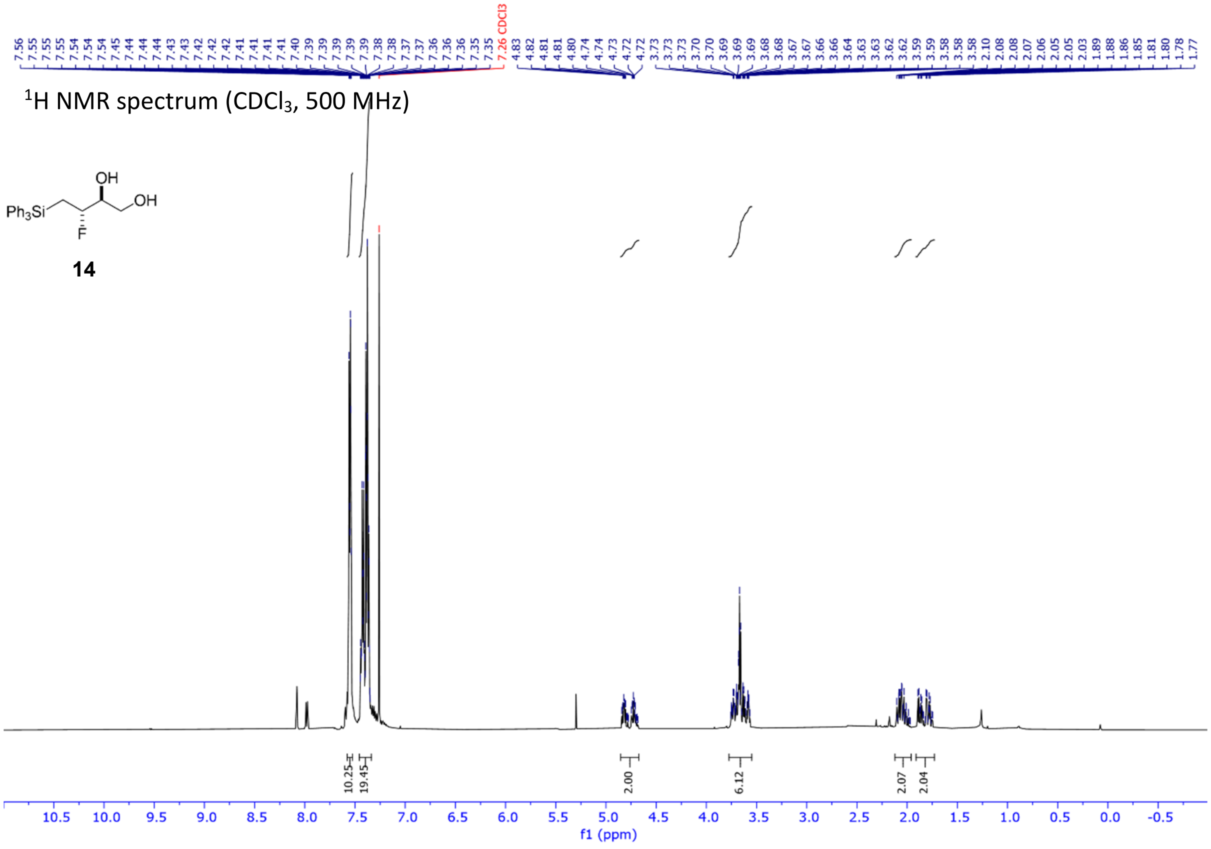

14

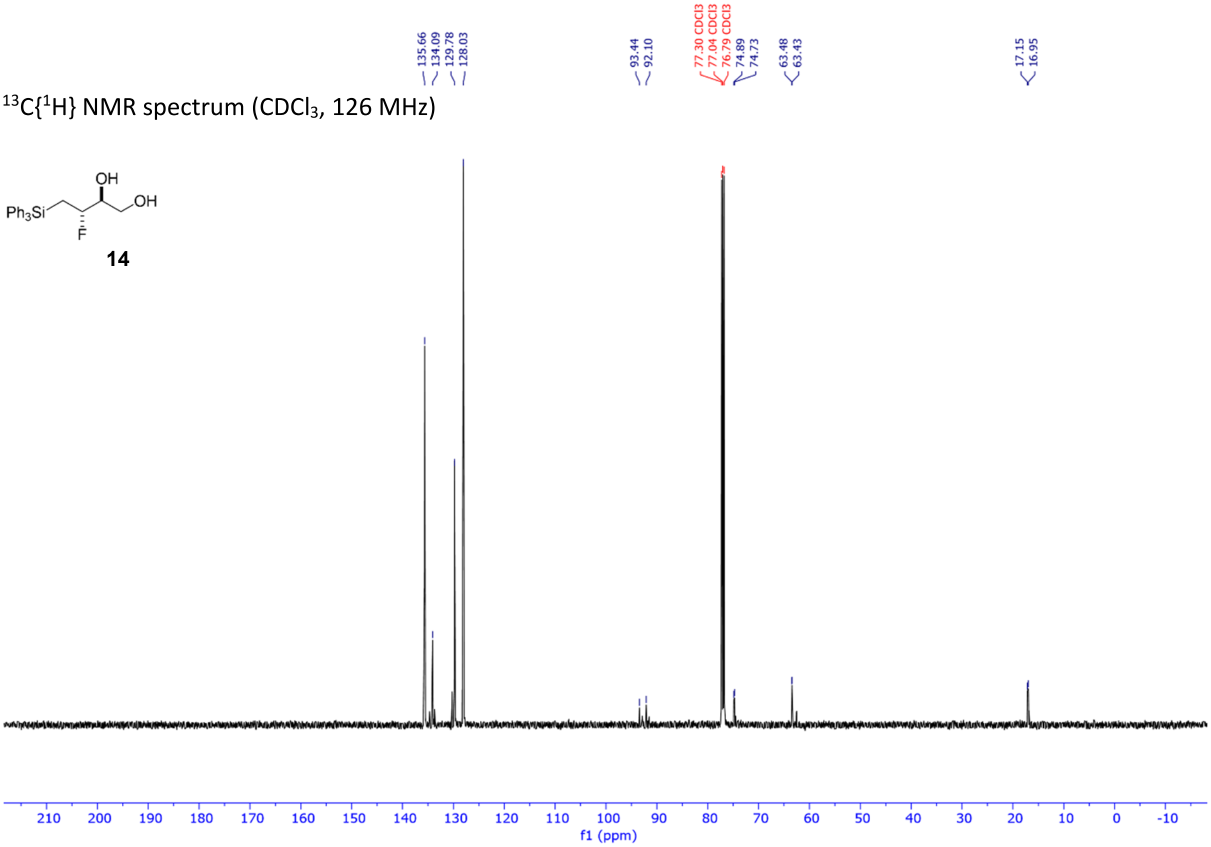
135.66
134.09
129.78
128.03
93.44
92.10
77.30 CDCl3
77.04 CDCl3
76.79 CDCl3
74.89
74.73
63.48
63.43
17.15
16.95
13C{1H} NMR spectrum (CDCl3, 126 MHz)
OH
Ph3Si
OH
F
14
f1 (ppm)

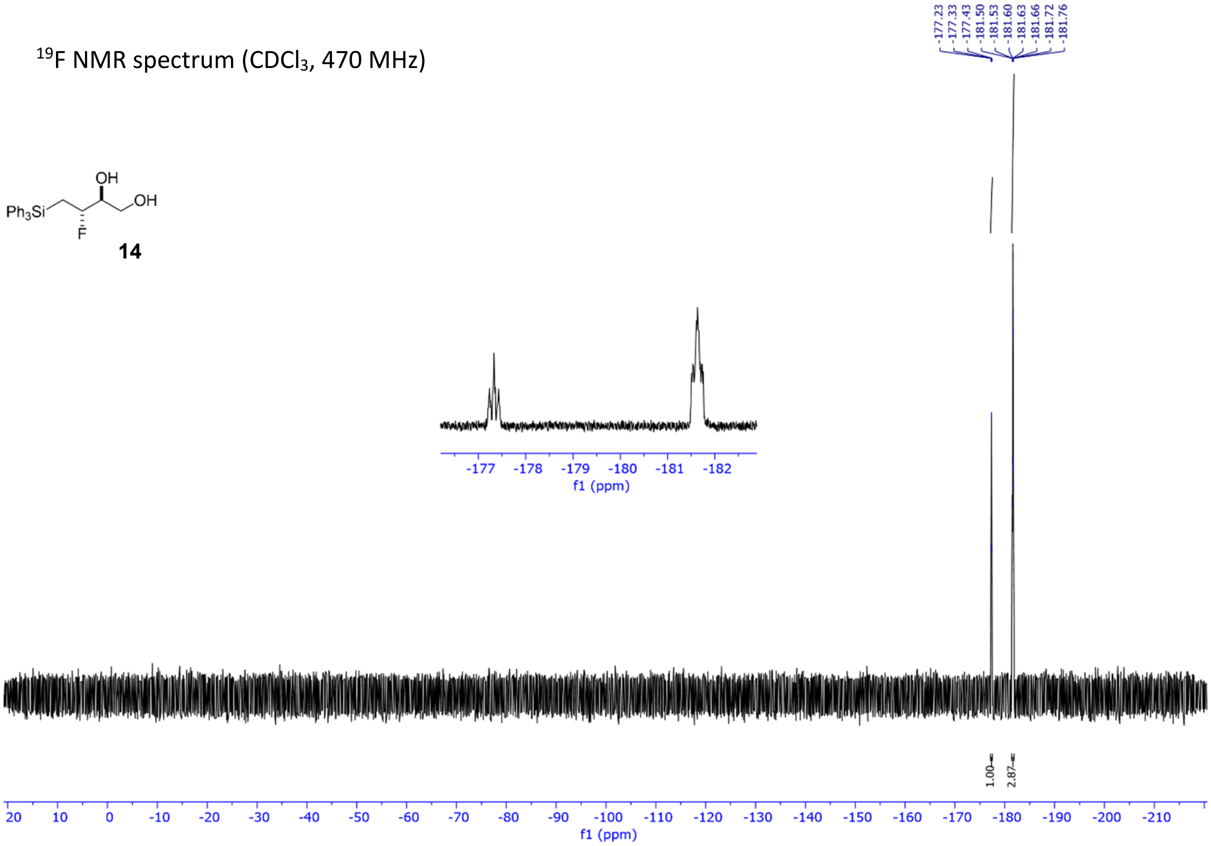
-177.23
-177.33
-177.43
-181.50
-181.53
-181.60
-181.63
-181.66
-181.72
-181.76
19F NMR spectrum (CDCl3, 470 MHz)
OH
Ph3Si
OH
F
14
f1 (ppm)
1.00
2.87
f1 (ppm)

^{1}H NMR spectrum ($CDCl_3$, 500 MHz)

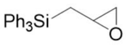

18

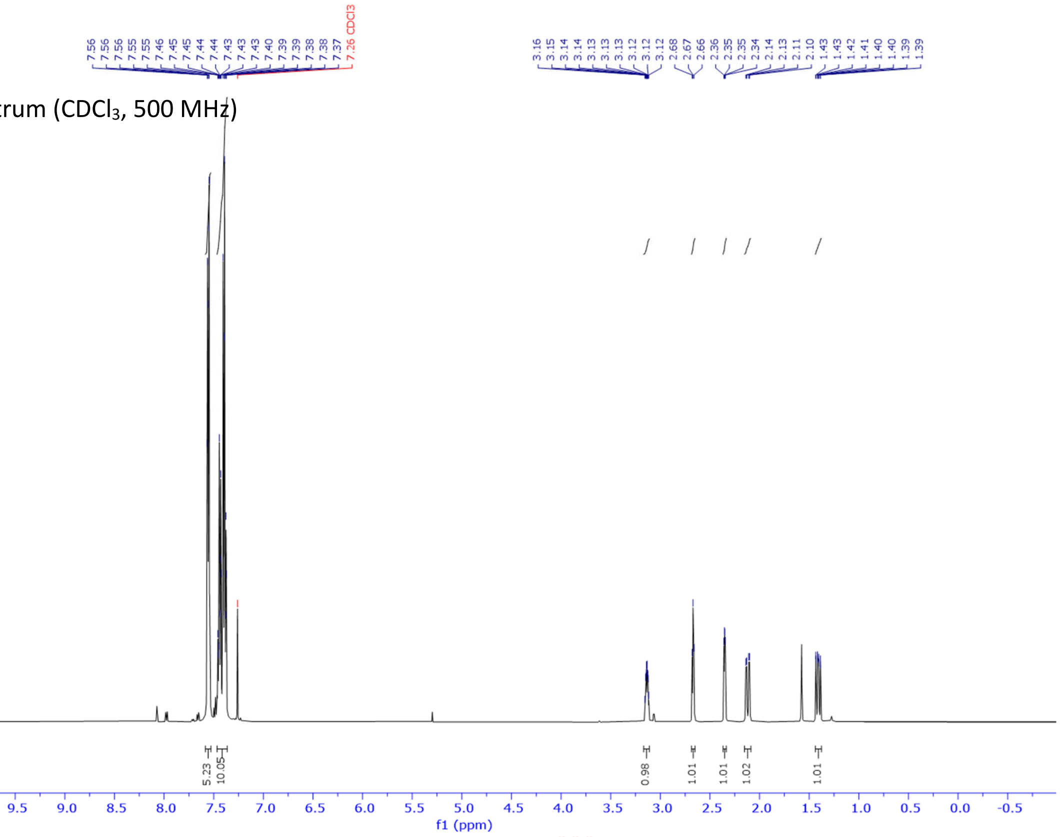

^{13}C{^{1}H} NMR spectrum ($CDCl_3$, 126 MHz)

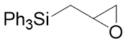

18

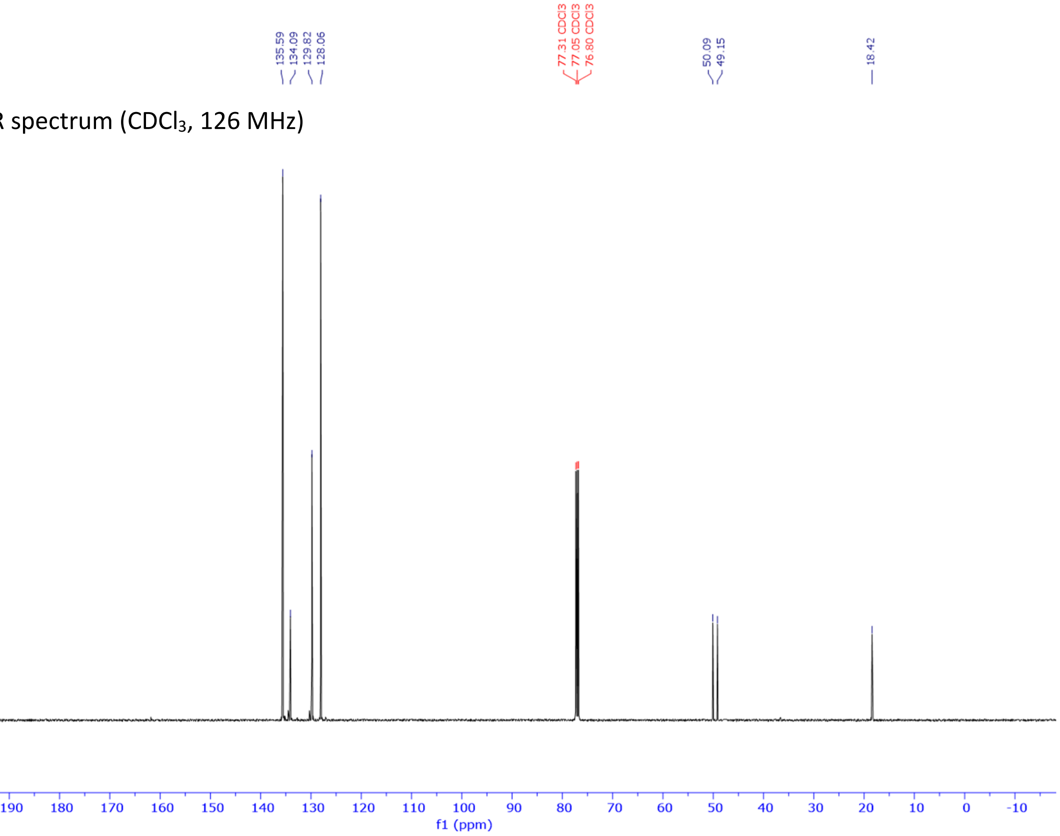

^{1}H NMR spectrum ($CDCl_3$, 500 MHz)

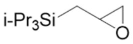

19

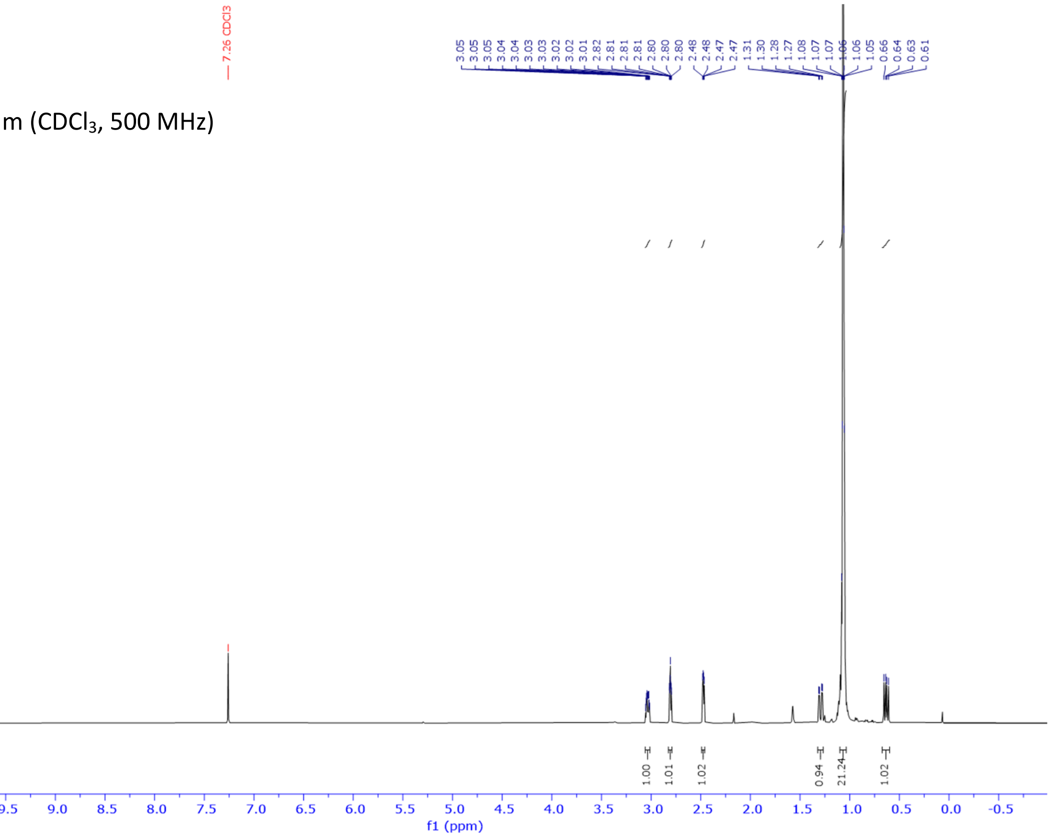

$^{13}C\{^1H\}$ NMR spectrum ($CDCl_3$, 126 MHz)

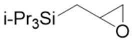

19

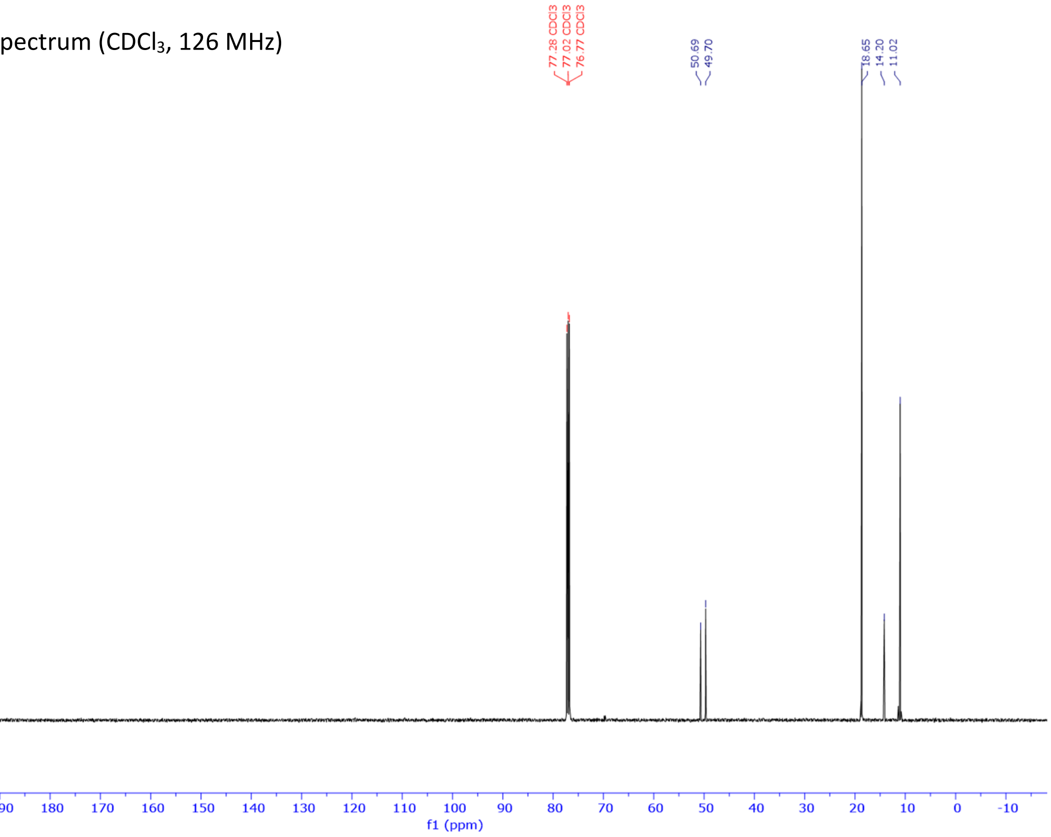

^{1}H NMR spectrum ($CDCl_3$, 500 MHz)

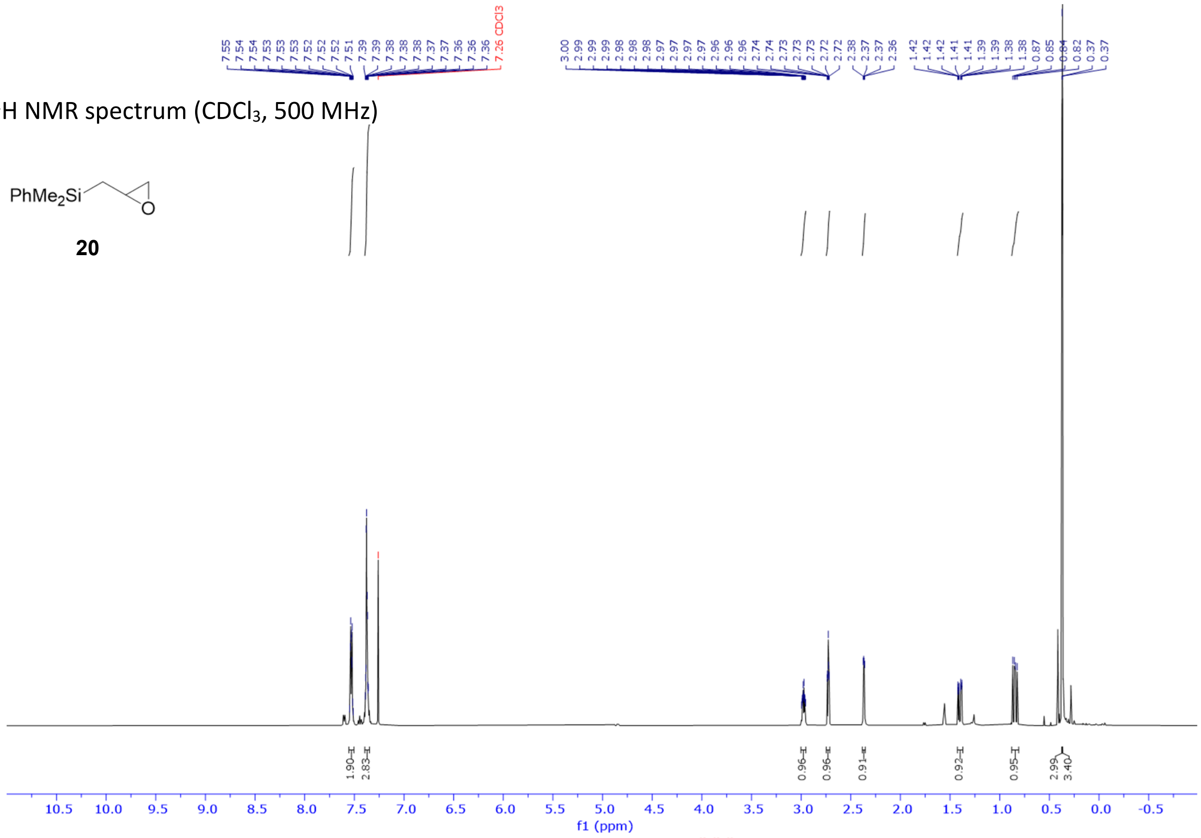

$^{13}C\{^{1}H\}$ NMR spectrum ($CDCl_3$, 126 MHz)

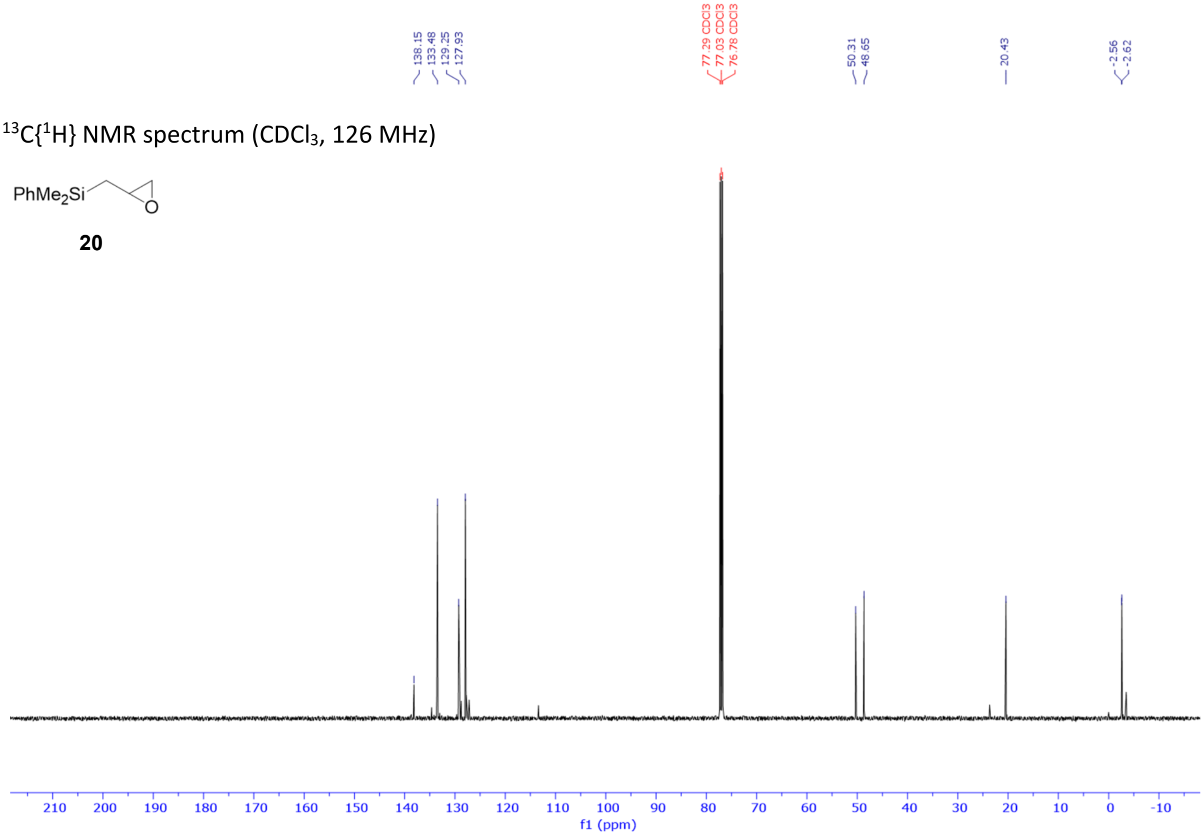

^{1}H NMR spectrum ($CDCl_3$, 500 MHz)

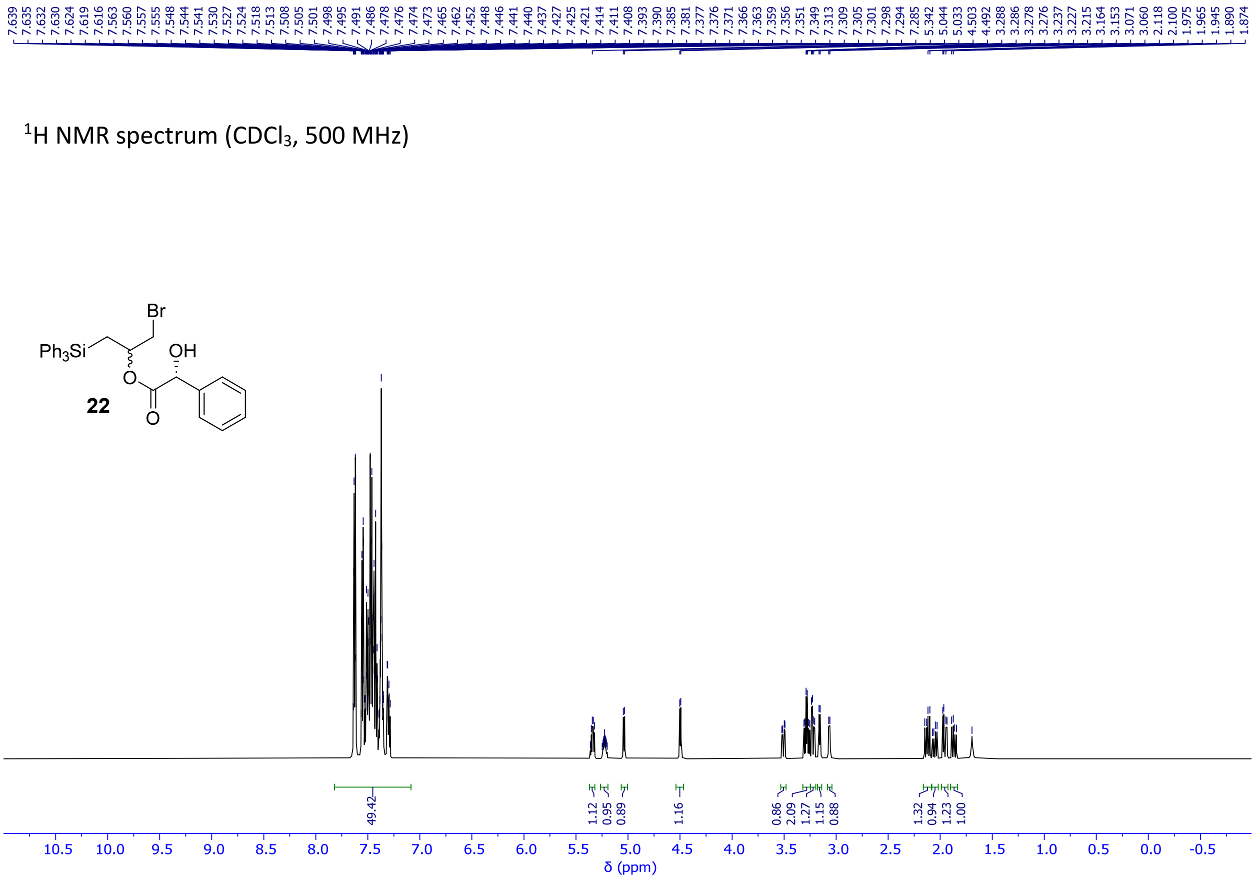

^{13}C{^{1}H} NMR spectrum ($CDCl_3$, 126 MHz)

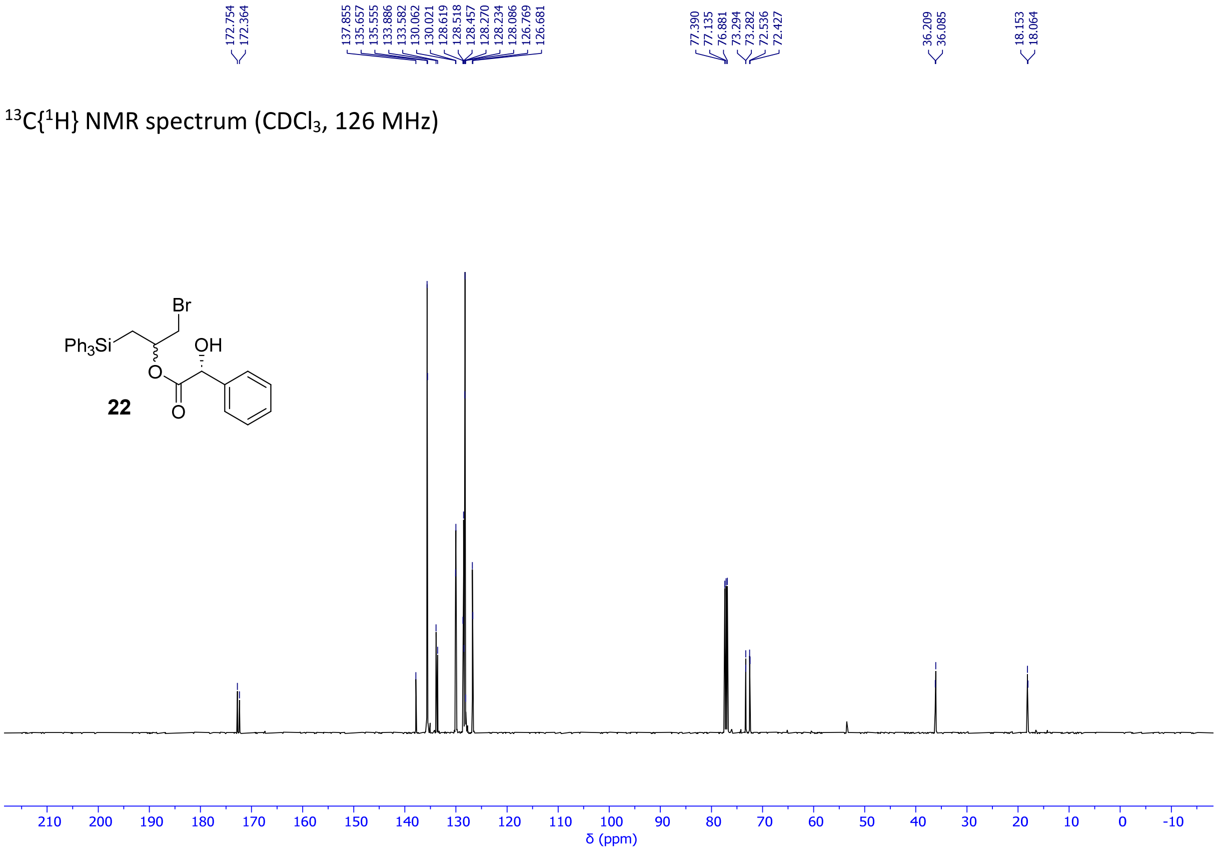

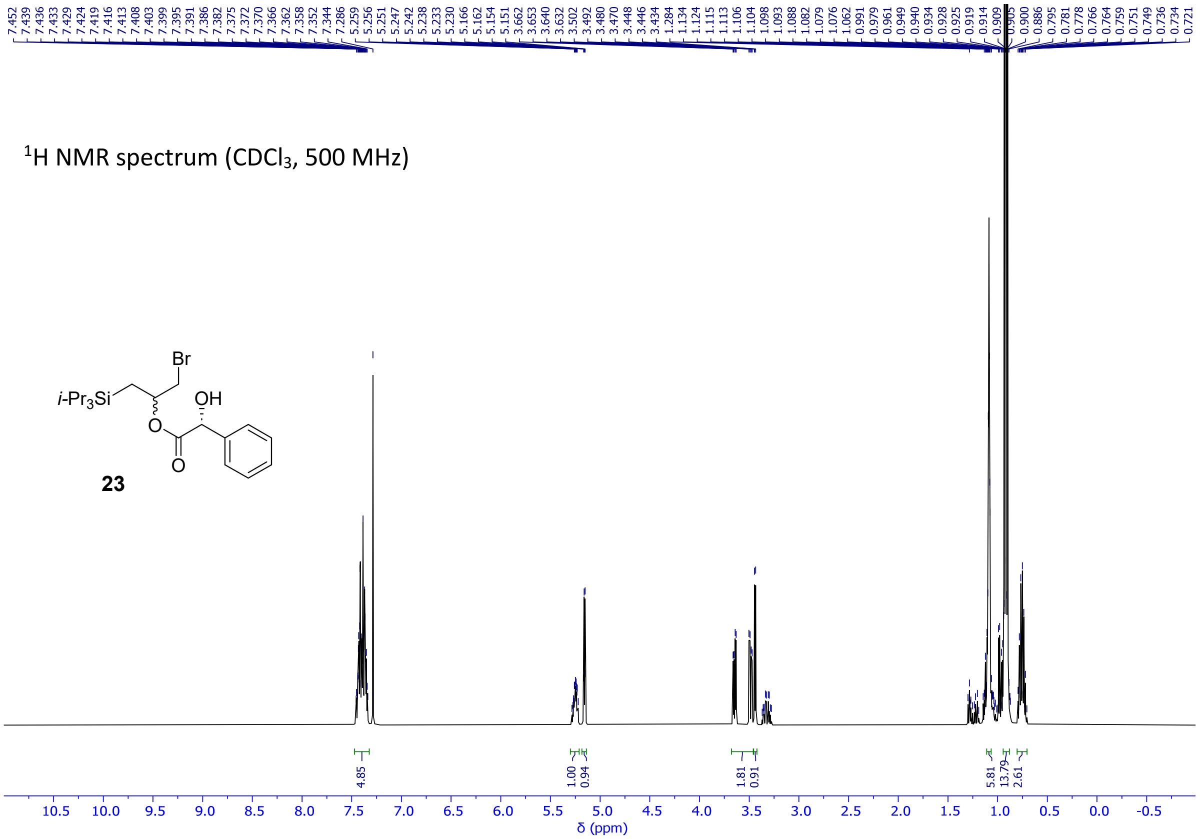
7.452 7.439 7.436 7.433 7.429 7.424 7.419 7.416 7.413 7.408 7.403 7.399 7.395 7.391 7.386 7.382 7.375 7.372 7.370 7.366 7.362 7.358 7.352 7.344 7.286 5.259 5.256 5.251 5.247 5.242 5.238 5.233 5.230 5.166 5.162 5.154 5.151 3.662 3.653 3.640 3.632 3.502 3.492 3.480 3.470 3.448 3.446 3.434 1.284 1.134 1.124 1.115 1.113 1.106 1.104 1.098 1.093 1.088 1.082 1.079 1.076 1.062 0.991 0.979 0.961 0.949 0.940 0.934 0.928 0.925 0.919 0.914 0.909 0.905 0.900 0.886 0.795 0.781 0.778 0.766 0.764 0.759 0.751 0.749 0.736 0.734 0.721
^{1}H NMR spectrum (CDCl$_3$, 500 MHz)
Br
i-Pr$_3$Si
OH
O
O
23
4.85
1.00
0.94
1.81
0.91
5.81
13.79
2.61
10.5 10.0 9.5 9.0 8.5 8.0 7.5 7.0 6.5 6.0 5.5 5.0 4.5 4.0 3.5 3.0 2.5 2.0 1.5 1.0 0.5 0.0 -0.5
δ (ppm)

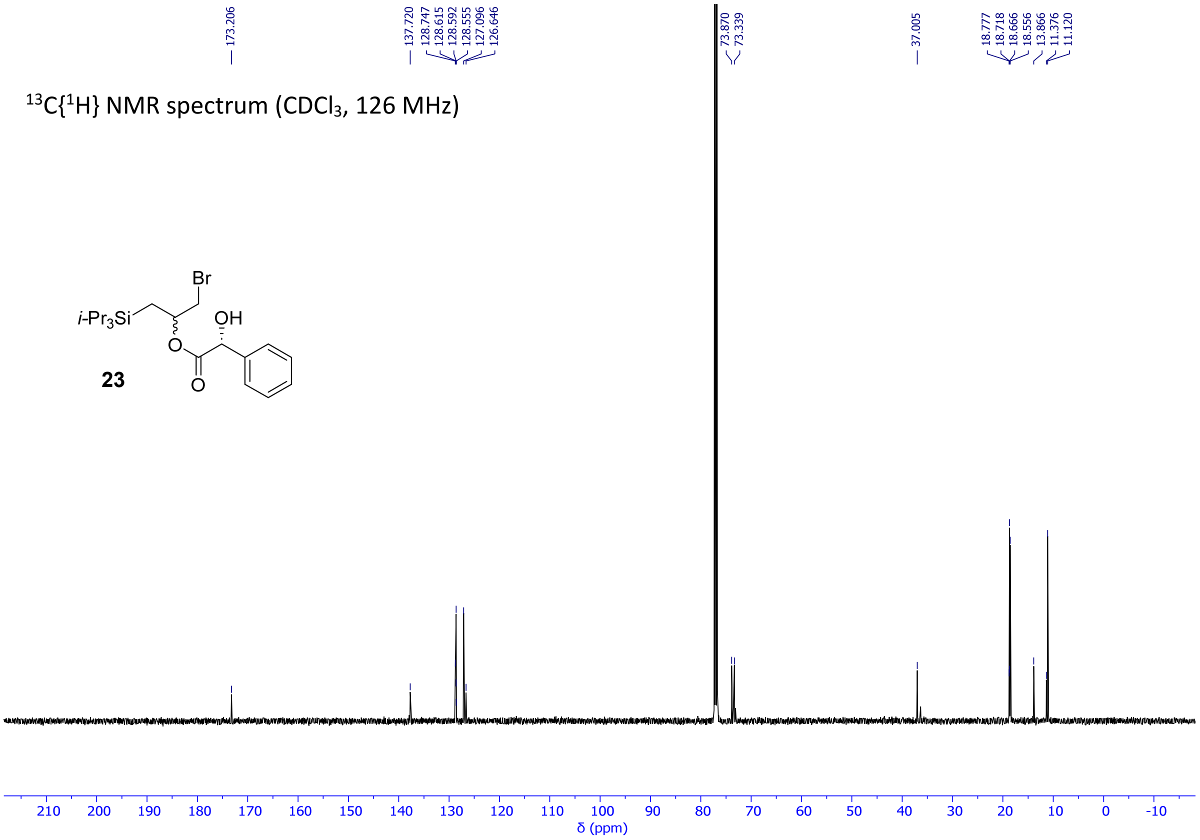
173.206
137.720 128.747 128.615 128.592 128.555 127.096 126.646
73.870 73.339
37.005
18.777 18.718 18.666 18.556 13.866 11.376 11.120
^{13}C{^{1}H} NMR spectrum (CDCl$_3$, 126 MHz)
Br
i-Pr$_3$Si
OH
O
O
23
210 200 190 180 170 160 150 140 130 120 110 100 90 80 70 60 50 40 30 20 10 0 -10
δ (ppm)

^{1}H NMR spectrum ($CDCl_3$, 500 MHz)

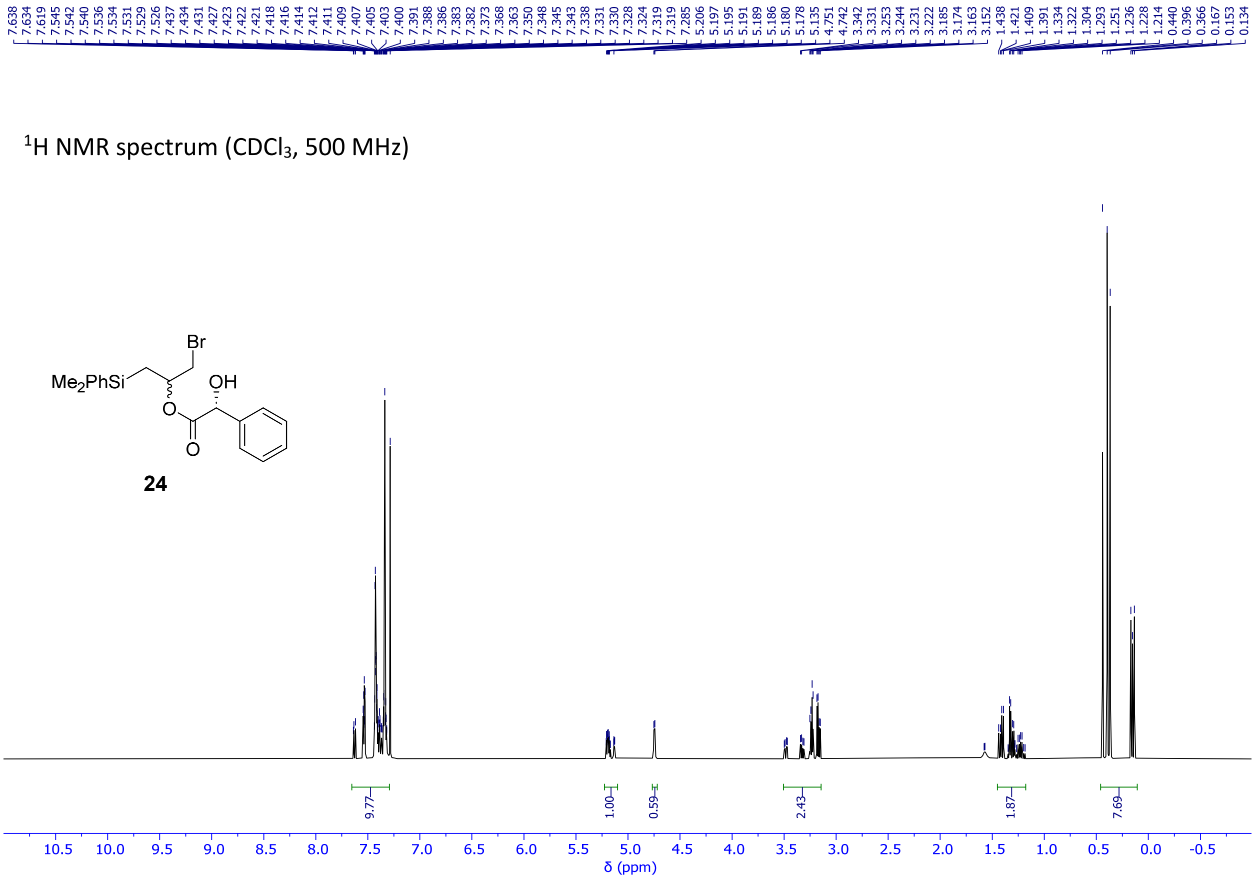

$^{13}C\{^{1}H\}$ NMR spectrum ($CDCl_3$, 126 MHz)

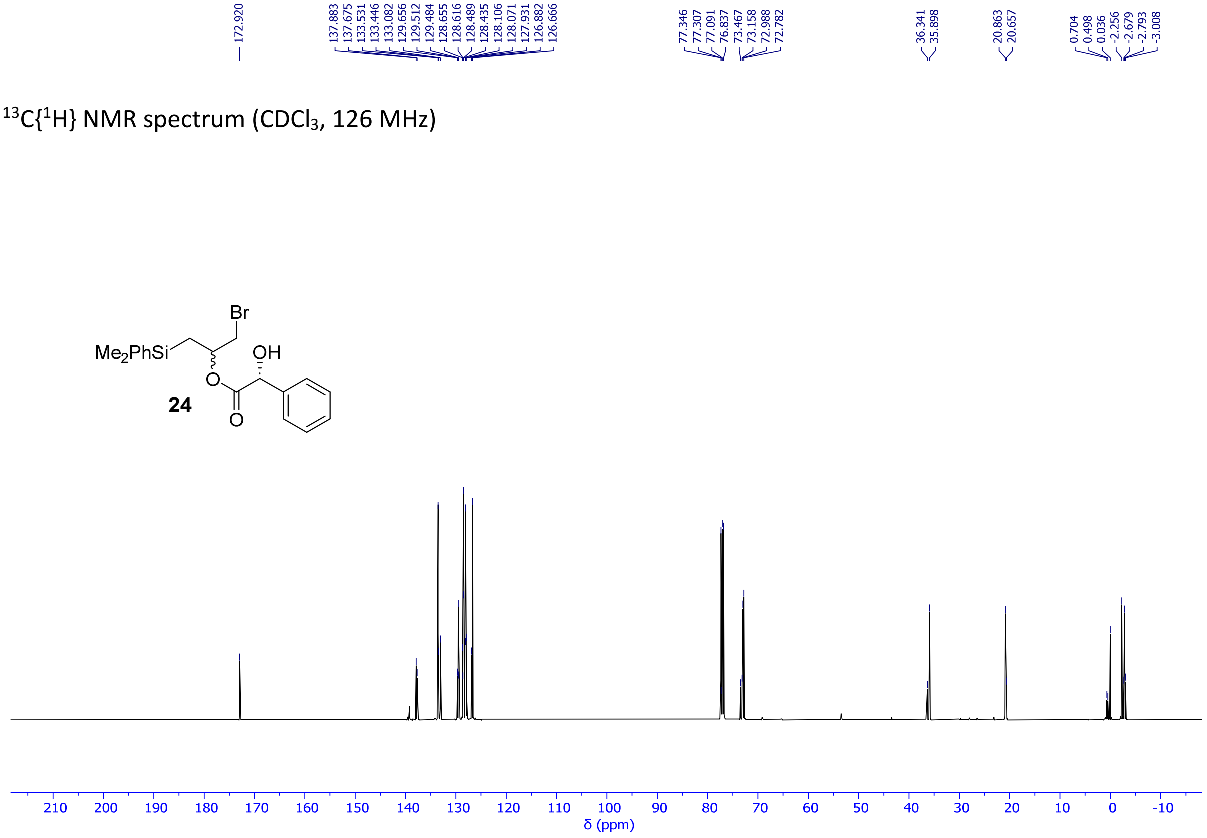

^{1}H NMR spectrum ($CDCl_3$, 500 MHz)

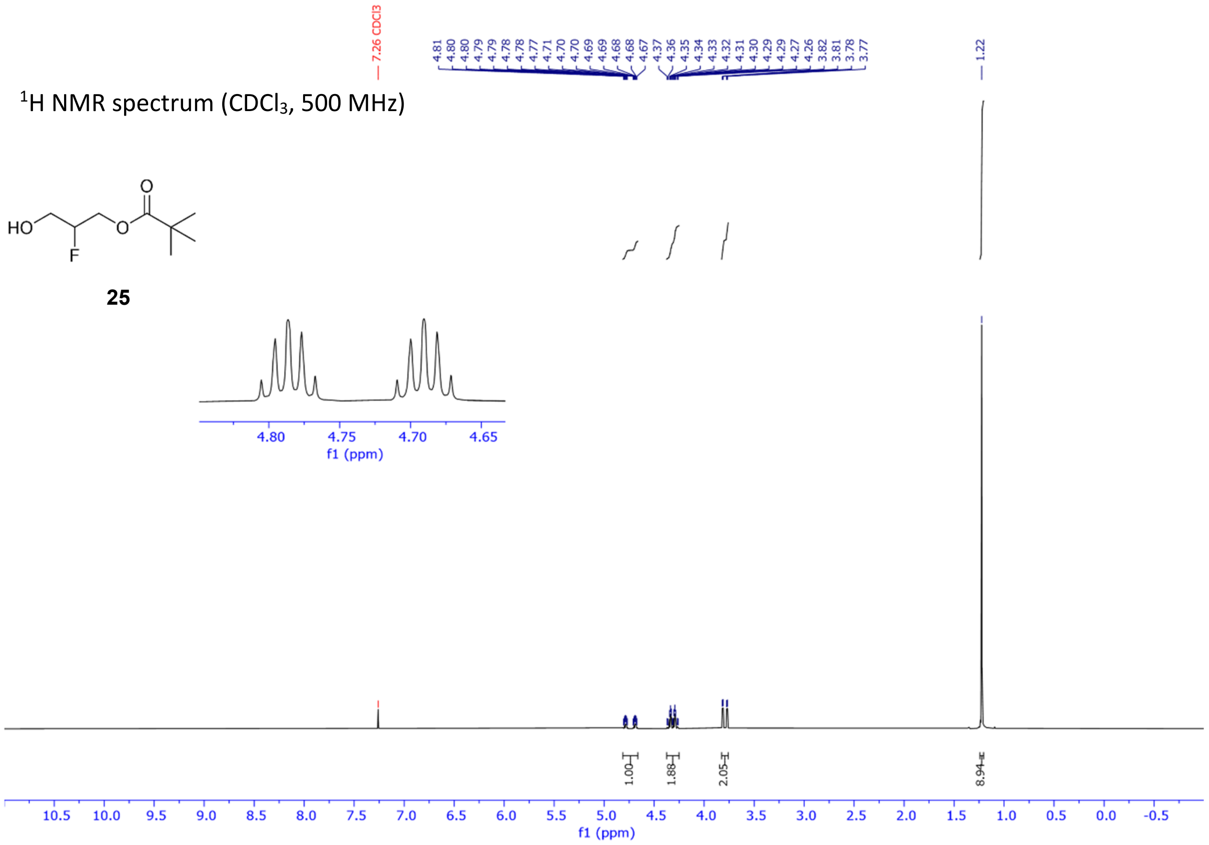

^{13}C{^{1}H} NMR spectrum ($CDCl_3$, 126 MHz)

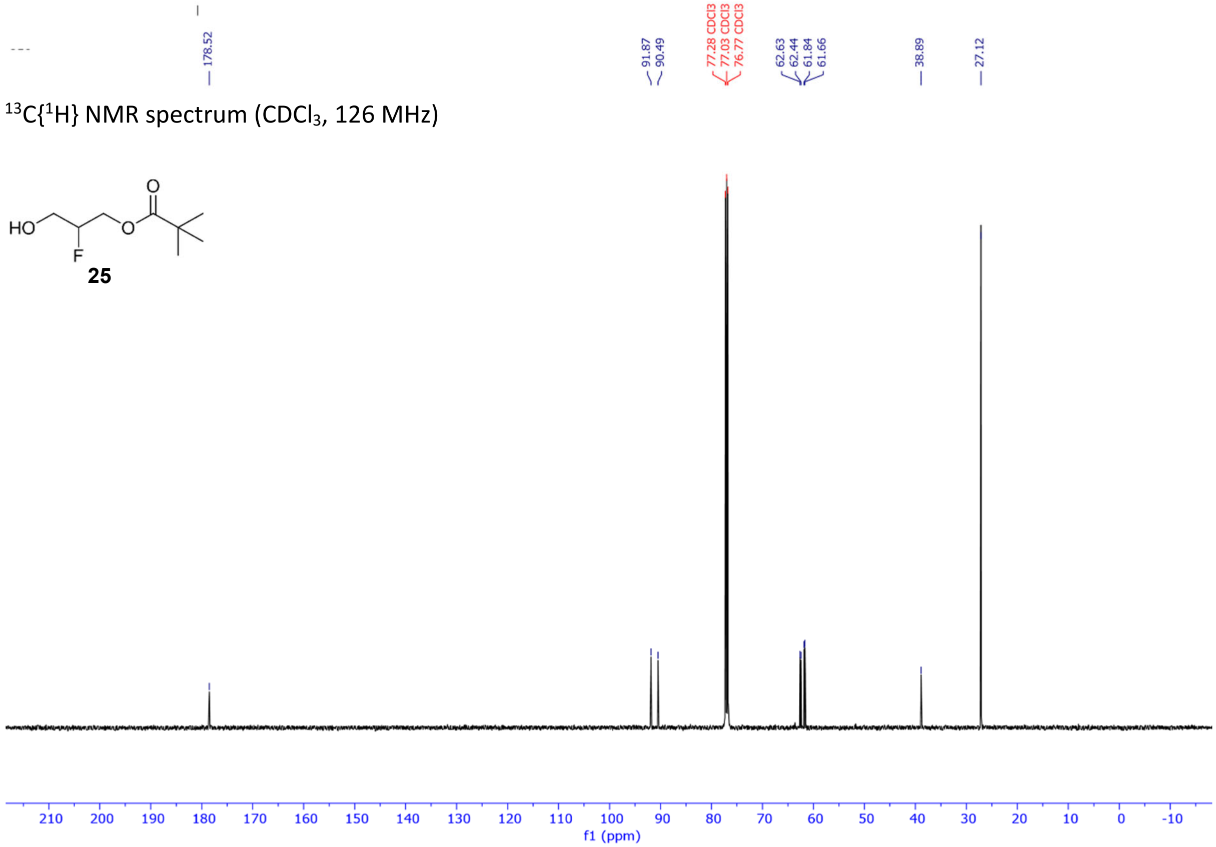

^{19}F NMR spectrum ($CDCl_3$, 470 MHz)

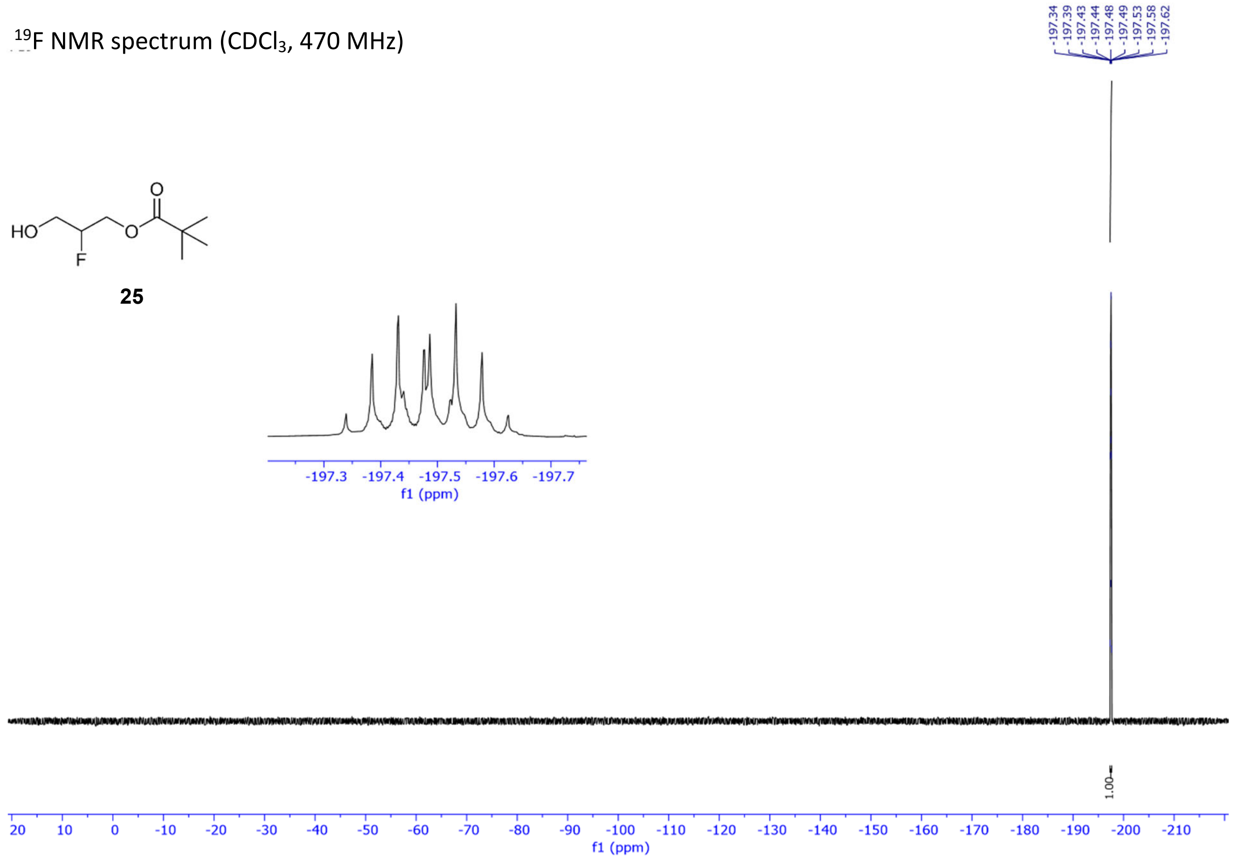

COSY spectrum ($CDCl_3$, 500 MHz)

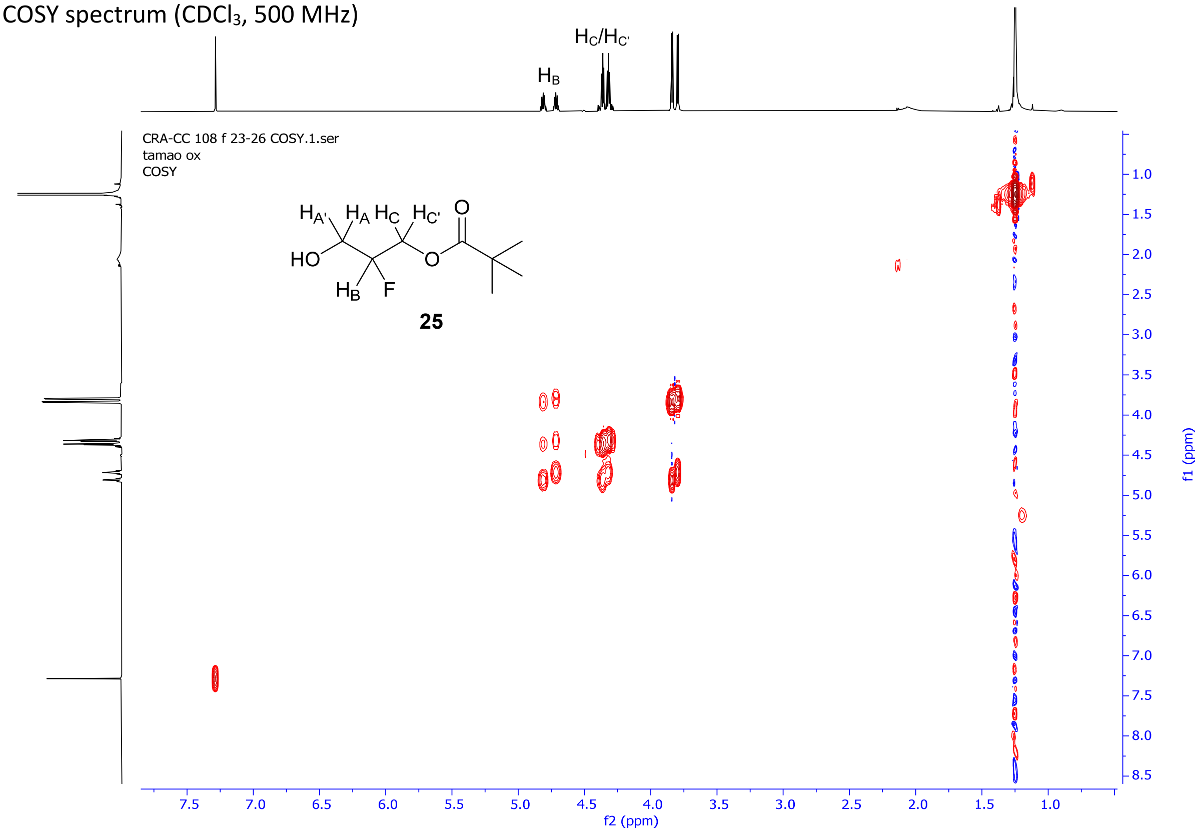

Table S1. Comparison of HF•Et_3N Equivalents on opening of epoxide **20**.

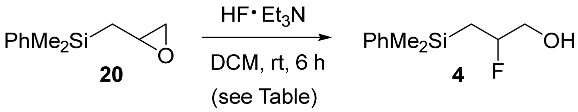

HF•Et_3N Equivalents[A]	% Conversion[B]
2.0	65.8
3.0	79.1
4.0	92.8
5.0	100

Notes for Table S1: [A]All reactions were performed by adding the indicated amount of HF•Et_3N to silyl epoxide **20** (0.5 mmol) in DCM (5 mL) and stirring for 6 h. [B]From ^{1}H NMR by comparing integration values for signals belonging to the fluorohydrin product (**4**) and starting epoxide (**20**).

Mosher Ester Analysis of Enantioenriched Silylfluorohydrins. Mosher esters of enantioenriched silylfluorohydrins were prepared according to the following general procedure: To a solution of the silylfluorohydrin (0.1 mmol) and (*S*)-methoxy-(trifluoromethyl)phenyl acetic acid (0.12 mmol) in toluene (5 mL) at -78 °C was added triethylamine (1.2 mmol) followed by DMAP (2.5 mmol) and 2,4,6-trichlorobenzoyl chloride (1.0 mmol) and the mixture was stirred while slowly warming to room temperature over 15 h. The reaction was quenched with aq. $NaHCO_3$ (25 mL), and extracted with EtOAc (25 mL). The organic phase was washed with aq. NaOH (1M, 25 mL), Brine (25 mL), dried over $MgSO_4$, filtered, and concentrated *in vacuo* before analysis by NMR (see Tables and accompanying Figures below).

Table S2. Results from Mosher ester analysis of enantioenriched silylfluorohydrins prepared by Shi epoxidation.

Silane	Mosher Ester d.r (CF_3)[A]	Mosher Ester d.r (OMe)[B]	Avg.
Ph_3	1.00 : 1.67	ND	--
iPr_3	1.00 : 1.52	1.00 : 1.60	1.00 : 1.56
$PhMe_2$	1.00 : 1.20	1.00 : 1.02	1.00 : 1.10

Notes for Table S1: (A) ^{19}F NMR integration values for the CF_3 signals of the Mosher ester2.39. (b) ^{1}H NMR integration values for the OCH_3 signals of the Mosher ester. ND = overlapping signals prevented accurate d.r. determination. Avg.: Average d.r. from those determined by ^{19}F and ^{1}H NMR.

Table S3. Results from Mosher ester analysis of enantioenriched silylfluorohydrins prepared by allylsilane bromomandelation.

**Silane**	**Bromandelation d.r**[A]	**Mosher Ester d.r (CF_3)**[B]	**Mosher Ester d.r (OMe)**[C]
Ph_3	1.00 : 3.85	1.00 : 2.61	1.00 : 2.41
iPr_3	1.00 : 4.04	1.00 : 3.80	1.00 : 4.17
$PhMe_2$	1.00 : 4.49	1.00 : 2.39	1.00 : 2.11

Notes for Table S2: (A) According to ^{1}H NMR analysis of the bromomandelate adduct. (B) ^{19}F NMR integration values for the CF_3 signals of the Mosher ester. (C) ^{1}H NMR integration values for the OCH_3 signals of the Mosher ester. ND = overlapping signals prevented accurate d.r. determination.

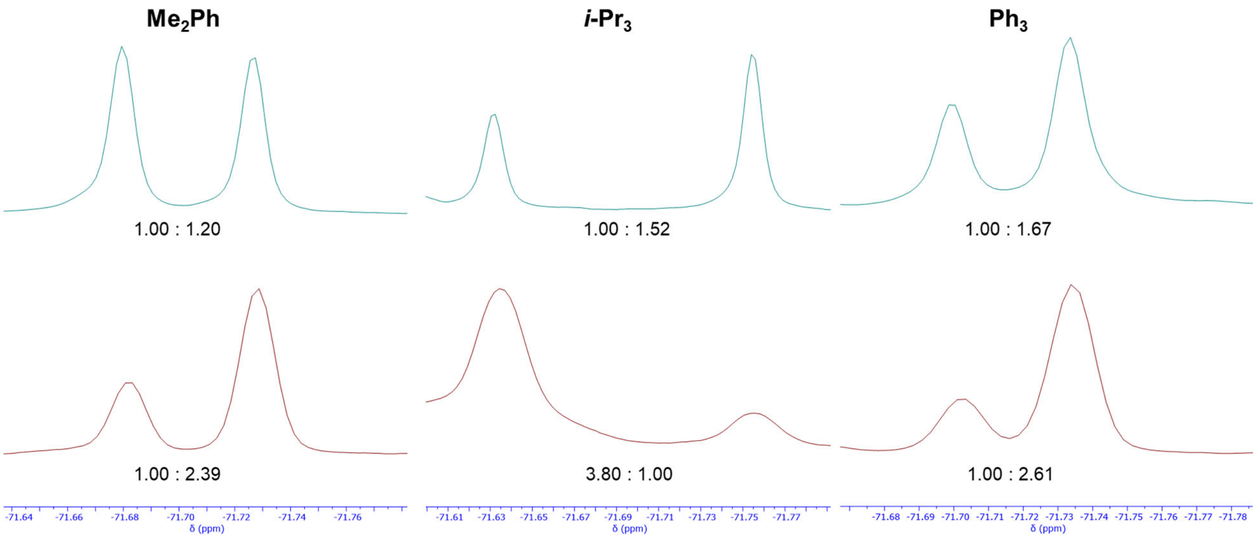

Figure S1. Comparison of ^{19}F NMR spectra for Mosher ester derivatives of enantioenriched silylfluorohydrins with the silicon substitution indicated, prepared by Shi epoxidation (top, blue traces) and bromomandelation (bottom, red traces). Values listed are integrations for the CF_3 signals from the two different diastereomers within each sample.

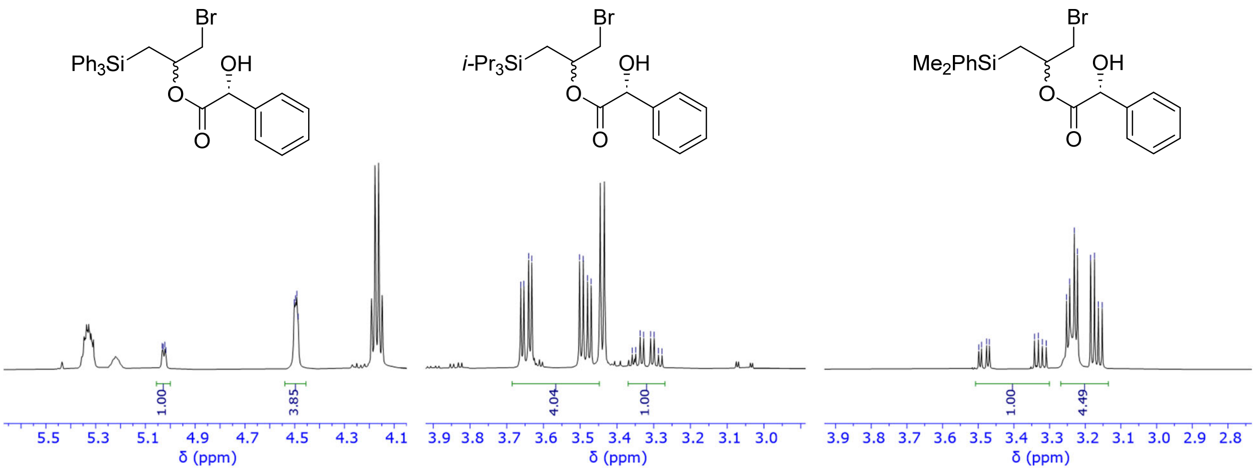

Figure S2. ^{1}H NMR spectra of bromomandelation products after partial separation of diastereomers by chromatography on silica, where the integration values shown correspond to the d.r. for the different samples (see Table S2).

Table S4: Crystallographic data for structures of compound **2**.

Empirical formula	C21 H21 F O Si	
Formula weight	336.47	
Temperature	100(2) K	
Wavelength	0.71073 Å	
Crystal system	Monoclinic	
Space group	C 2	
Unit cell dimensions	a = 32.472(4) Å	α= 90°.
	b = 7.0912(8) Å	β= 105.826(7)°.
	c = 15.961(2) Å	γ = 90°.
Volume	3536.0(7) Å^3	
Z	8	
Density (calculated)	1.264 Mg/m^3	
Absorption coefficient	0.147 mm^{-1}	
F(000)	1424	
Crystal size	0.375 x 0.100 x 0.080 mm^3	
Theta range for data collection	1.304 to 25.148°.	
Index ranges	-38<=h<=37, -8<=k<=8, -18<=l<=19	
Reflections collected	14857	
Independent reflections	6584 [R(int) = 0.0858]	
Completeness to theta = 25.148°	100.0 %	
Refinement method	Full-matrix least-squares on F^2	
Data / restraints / parameters	6584 / 849 / 442	
Goodness-of-fit on F^2	1.038	
Final R indices [I>2sigma(I)]	R1 = 0.0695, wR2 = 0.1668	
R indices (all data)	R1 = 0.1020, wR2 = 0.1943	
Absolute structure parameter	-0.21(9)	
Largest diff. peak and hole	0.282 and -0.240 e.Å^{-3}	
